# Integrated GlycoProteome Analyzer (I-GPA) for Automated Identification and Quantitation of Site-Specific N-Glycosylation

**DOI:** 10.1038/srep21175

**Published:** 2016-02-17

**Authors:** Gun Wook Park, Jin Young Kim, Heeyoun Hwang, Ju Yeon Lee, Young Hee Ahn, Hyun Kyoung Lee, Eun Sun Ji, Kwang Hoe Kim, Hoi Keun Jeong, Ki Na Yun, Yong-Sam Kim, Jeong-Heon Ko, Hyun Joo An, Jae Han Kim, Young-Ki Paik, Jong Shin Yoo

**Affiliations:** 1 Department of Mass Spectrometry, Korea Basic Science Institute, Ochang, Republic of Korea; 2 Graduate School of Analytical Science and Technology, Chungnam National University, Daejeon, Republic of Korea; 3 Department of Biomedical Science, Cheongju University, Cheongju, Republic of Korea; 4 Department of Chemistry, Hannam University, Daejeon, Republic of Korea; 5 Department of Chemistry, Sogang University, Seoul, Republic of Korea; 6 Cancer Biomarkers Development Research Center, Korea Research Institute of Bioscience and Biotechnology, Daejeon, Republic of Korea; 7 Department of Food Nutrition, Chungnam National University, Daejeon, Republic of Korea; 8 Yonsei Proteome Research Center and Department of Integrated OMICS for Biomedical Science, and Department of Biochemistry, College of Life Science and Biotechnology, Yonsei University, Seoul, Republic of Korea

## Abstract

Human glycoproteins exhibit enormous heterogeneity at each N-glycosite, but few studies have attempted to globally characterize the site-specific structural features. We have developed Integrated GlycoProteome Analyzer (I-GPA) including mapping system for complex N-glycoproteomes, which combines methods for tandem mass spectrometry with a database search and algorithmic suite. Using an *N-*glycopeptide database that we constructed, we created novel scoring algorithms with decoy glycopeptides, where 95 *N-*glycopeptides from standard α1-acid glycoprotein were identified with 0% false positives, giving the same results as manual validation. Additionally automated label-free quantitation method was first developed that utilizes the combined intensity of top three isotope peaks at three highest MS spectral points. The efficiency of I-GPA was demonstrated by automatically identifying 619 site-specific *N-*glycopeptides with FDR ≤ 1%, and simultaneously quantifying 598 *N-*glycopeptides, from human plasma samples that are known to contain highly glycosylated proteins. Thus, I-GPA platform could make a major breakthrough in high-throughput mapping of complex N-glycoproteomes, which can be applied to biomarker discovery and ongoing global human proteome project.

Protein N-glycosylation, one of the most prevalent post-translational modifications (PTMs) in proteins, plays important roles in biological systems via its influence on various processes, including adhesion, signaling through cellular recognition, and response to abnormal biological states. Because each N-glycosite on a glycoprotein consists of a mixture of numerous glycoforms, each protein glycoform is generally present at low concentrations (i.e., sub-stoichiometric). Alterations in the distribution of protein glycoforms, as well as the presence of aberrant glycoforms, are closely associated with a variety of illnesses, including cancer^1^ and neurodegenerative diseases^2^. The ability to identify of aberrant protein glycoforms and monitor changes in protein glycoform distribution in biological and clinical samples would facilitate a deeper understanding of glycoprotein structure–function relationships and would also aid in discovery of biomarkers associated with aberrant glycosylation.

Glycoproteomics has attracted a great deal of attention in recent decades. Mass Spectrometry (MS) technology, a powerful tool in proteomics in general, is also a core tool in glycoproteomics^3^. Nonetheless, efficient high-throughput global mapping of complex glycoproteomes is very difficult, mainly due to the exceptional complexity of the chemical and physical features of glycoprotein. To overcome these problems, various approaches have been explored, including glycan composition profiling focusing on glycan moieties released from glycoprotein^4^,^5^, glycosite profiling focusing on deglycosylated sites following endoglycosidase treatment^6^,^7^,^8^, and differential quantitation of protein glycoforms fractionated by glycan structure^9^,^10^. However, because each of these approaches can provide only limited information, they must be used in combination in order to obtain a comprehensive picture^11^,^12^.

By contrast, because glycopeptides encompass intact glycan and peptide moieties together within the same molecule, a glycoproteomic approach that profiles *N-*glycopeptides could provide comprehensive information regarding protein N-glycosylation^13^. The concomitant presence of glycan and peptide moieties with different physical and chemical properties makes the full structural characterization of *N-*glycopeptide extraordinarily difficult. However, previous works have shown that various powerful tandem MS fragmentation techniques enable direct identification of intact *N-*glycopeptides and monitoring of the site-specific glycoform distributions of pure glycoproteins isolated from clinical specimens such as organ tissue and plasma in which most of proteins are heterogeneously glycosylated in complex mixtures^14^,^15^,^16^. Recently, large-scale site-specific *N-*glycopeptide identification has been attempted using complex glycoproteome samples^17^,^18^,^19^. However, high-throughput global mapping of site-specific glycopeptides of *N-*glycoproteins in blood samples is much more challenging, due to the extremely high degree of sample complexity, wide dynamic ranges of the abundance of analytes, and current lack of automatic search algorithms capable of confidently identifying *N-*glycopeptides from large amounts of tandem MS data. There are some search algorithms such as GlycoFragwork^19^, GP Finder^20^, Sweet-Heart^21^, GPS^22^, Byonic^23^, and MAGIC^24^ to identify glycopeptides with false discovery rate (FDR) less than 5%. Even using the best algorithms, an FDR of 1% does not actually give you 1% false positives. For example, S. W. Wu *et al.*^25^ reported more than 37% false positives in the analysis of a protein despite of a claim of zero FDR. Frequently, we need manual validation of individual tandem mass spectra, which is tedious and takes long time.

To address these issues, in this study we attempted to develop a fast search engine termed Integrated GlycoProteome Analyzer (I-GPA), which is capable of identification of site-specific *N-*glycopeptides without manual validation and automated label-free quantitation of large capacity of glycoproteins. We then newly developed platform to high-throughput comparative mapping and quantifying glycoproteomes present in the plasma of liver cancer patients (hepatocellular carcinoma: HCC) and healthy donor controls, with the goal of revealing potential novel *N-*glycopeptide biomarkers. Here we show that I-GPA is a new automated N-glycoproteome analyzer which facilitates high-speed mapping and quantifying glycoproteins, suitable for ongoing chromosome-centric human proteome project (C-HPP)^26^.

## Results

**Figure 1:**
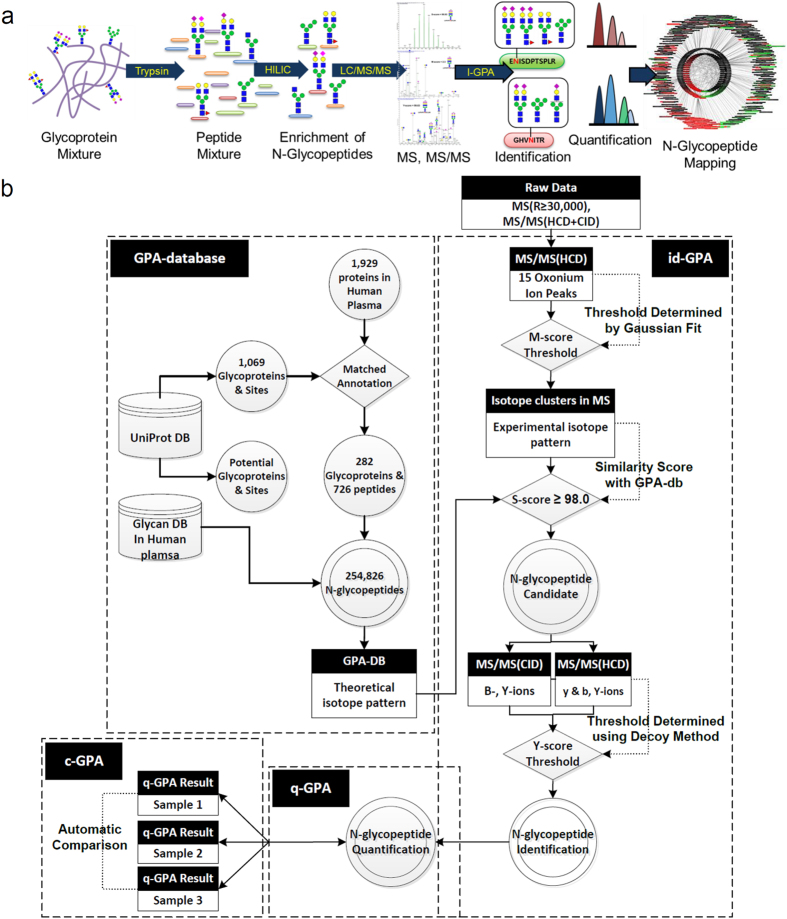
Structural and functional components of the Integrated *N-*glycoproteome analyzer (I-GPA). (**a**) Schematic workflow for site-specific glycoform analysis of intact *N-*glycopeptides by I-GPA*. N*-glycopeptides enriched from tryptic digests of glycoproteome samples using HILIC were analyzed by high-resolution MS with a combination of HCD and CID fragmentation, followed by analysis using the automated search engine I-GPA. (**b**) Schematic algorithm of site-specific glycoform analysis of *N*-glycopeptides by I-GPA. I-GPA consists of a GPA-DB (e.g., 254,826 *N*-glycopeptides in human plasma), an identification component (id-GPA), a quantitation component (q-GPA), and comparative GPA (c-GPA). The *N-*glycopeptide database for I-GPA was automatically constructed using the program GPA-DB-Builder. The id-GPA search algorithm was used for *N*-glycopeptide identification from the MS and MS/MS (HCD/CID) data using M-, S-, and Y-scores as criteria for *N-*glycopeptide identification. The identified *N-*glycopeptides were then quantitated by q-GPA by summation of the top three-isotope peaks from the MS1 spectral points. c-GPA can compare the abundance of an *N*-glycopeptide between glycoproteome samples by the top three-isotopes quantitation (3TIQ).

Given that most of proteins present in human plasma are glycoproteins we chose plasma sample for construction of plasma glycoprotein DB and analysis of glycoproteins in this study. We wanted to design a concept of I-GPA which may facilitate the high-throughput analysis of the N-glycoproteins with tandem MS and previously built in-house glycoproteome DBs. To this end, we first analyzed the HILIC-enriched site-specific *N-*glycopeptides of human plasma by nano-reversed-phase liquid chromatography (nRPLC) coupled to MS with both HCD and CID-MS/MS fragmentation. The resultant data were then computationally analyzed using specific algorithms suite within the I-GPA platform: *N-*glycopeptides were identified against the GPA-DB (id-GPA), quantitated (q-GPA), and finally compared between multiple samples (c-GPA) as outlined in Fig. 1a,b. All resultant data as well as mass spectrometry raw data have been deposited to the ProteomeXchange Consortium via the MassIVE repository with the dataset identifier PXD003369 or MSV000079426, respectively. The RAW files are available for download at ftp://massive.ucsd.edu/MSV000079426/.

### Construction and evaluation of composite GPA-DBs containing GPA-DB-AGP, GPA-DB-Mixture, and GPA-DB-Human Plasma

GPA-DBs were constructed for each sample, using the software GPA-DB-Builder, by combining possible tryptic peptides and 351 N-linked glycans, where 331 retrosynthetic glycans came from references of Kronewitter, S.R. *et al.*^27^ and 20 glycans from penta and hexa polylactosamine series of Ozohanics, O. *et al.*^28^ (Supplementary Excel 1). The GPA-DB includes isotope pattern information for masses and relative intensities of intact *N-*glycopeptides. GPA-DB-AGP (n = 4,212), GPA-DB-Mixture (n = 6,318), and GPA-DB-HumanPlasma (n = 254,826)^29^ were used for the analysis of α1-acid glycoprotein (AGP), three standard protein mixtures, and depleted (or non-depleted) human plasma, respectively (see Supplementary Note 1, Supplementary Fig. 1, Supplementary Table 1, and Supplementary Excels 2, 3).

### Automated identification of *N-*glycopeptides using id-GPA algorithms

id-GPA was designed to automatically identify site-specific *N-*glycopeptides using converted to MS (.txt) and MS/MS (.mgf) format (see Supplementary Note 2). Scoring entailed three steps: 1) Selection of *N-*glycopeptide from 15 glycan-specific oxonium ions using HCD-MS/MS spectra; (M-score); 2) Selection of candidates by matching the isotope pattern to intact *N-*glycopeptides in the GPA-DB (S-score); and 3) Identification of *N-*glycopeptide from CID and HCD-MS/MS fragment ions (Y-score) with FDR ≤ 1%.

### Selection of *N-*glycopeptide spectra using M-score

**Figure 2:**
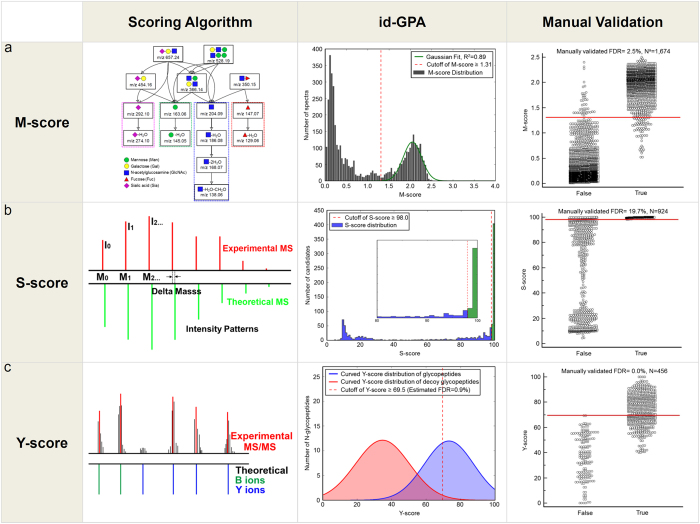
Computational algorithm of id-GPA for identification of standard α1-acid glycoprotein (AGP). id-GPA was designed for identification of *N*-glycopeptides in high-throughput analysis using three scoring systems (M-, S-, and Y-scores). (**a**) M-score for *N-*glycopeptide selection based on 15 glycan-specific oxonium ions from the total HCD-MS/MS spectra; N = 1,674, number of selected *N*-glycopeptide spectra for subsequent analysis with M-score > 1.3 from a total of 5,818 HCD spectra, at a false discovery rate (FDR) of 2.5% (as determined by manual validation). (**b**) S-score for the selection of *N*-glycopeptide candidates by matching the isotope distribution of *N*-glycopeptides in the database; n = 924, number of selected precursor ions of *N-*glycopeptide candidates with an S-score > 98.0 at an FDR of 19.7% (determined by manual validation). (**c**) Y-score for identification of *N*-glycopeptides by matching fragment ions in the CID and HCD spectra with the original 924 *N*-glycopeptide candidates. Ultimately, N = 456 *N*-glycopeptide spectra were identified with Y-score > 69.5 at an FDR of 0.0% (as determined by manual validation).

We noted that glycan oxonium ions, singly protonated mono- and oligo-saccharide ions resulting from fragmentation of glycans and glycopeptides, are highly sensitive markers of glycopeptide fragmentation in HCD-MS/MS spectra^30^. Generally previous studies have used only 3–5 oxonium ions in HCD spectra just to manually “flag” glycopeptide data without knowledge of their statistical distribution in MS/MS spectra. On the other hand, we use total 15 oxonium ions (Fig. 2a, left) differently weighted according to their frequency of appearance in HCD spectra (for example m/z 204, 186, 168, 138 series, etc.), and then determined how often they appeared in HCD-MS/MS spectra using the M-score calculated using Equation 1 (Supplementary Note 3).


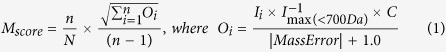


where N is the number of expected oxonium ion, n is the number of matched oxonium ion, I_i_ is the peak intensity of matched oxonium ion, I_max(<700Da)_ is the highest intensity of peak less than 700Da, MassError is absolute difference of the mass of matched peak from the theoretical mass of the oxonium ion, and C is the theoretical frequency of appearance in HCD spectra. MassError +1.0 was considered not to make the O_i_ infinity. If the mass error is zero, O_i_ represents the weighted relative intensity of matched oxonium ion. The M-score allows us to select only those MS/MS spectra that contain markers of *N*-glycopeptides from the large number of spectra obtained during an LC-MS run. We analyzed tryptic digests of a standard AGP, which has glycoforms of complex types. Fig. 2a (middle) shows the M-score distribution of the HCD-MS/MS spectra. Most MS/MS spectra had an M-score of <0.5, but higher M-scores were also present. After Gaussian fitting, we can automatically select 1,674 MS/MS spectra with M-score ≥ 1.3 from a total of 5,818 spectra. We manually confirmed those spectra contained markers of 15 oxonium ions with an FDR of 2.5% (Fig. 2a, right). The selection of *N-*glycopeptides by M-score was usefully presented in the analysis of same sample with enrichment by HILIC. Its distribution (Supplementary Fig. 2, Supplementary Table 2) revealed most *N-*glycopeptides with an M-score ≥ 1.6 by HILIC enrichment.

### Selection of *N-*glycopeptide candidates using S-score

Once the MS/MS spectra were selected by M-score, we obtained isotope patterns of their precursor ions, and then searched against the previously constructed GPA-DB for the best match. We compared the isotope patterns of molecular ions between experimental and theoretical data (Fig. 2b, left) and calculated the similarity (S-score) to select *N-*glycopeptide candidates according to Equation 2.


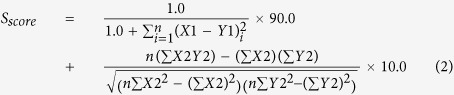


where X1 is the m/z of theoretical isotope peak, X2 is the intensity of theoretical isotope peak, Y1 is the m/z of experimental isotope peak, Y2 is the relative intensity of experimental isotope peak. When calculating the S-score, we considered mass accuracy and relative intensity at a ratio of 9:1, because the best AUC (Area Under ROC Curves) value for true *N-*glycopeptide matching in the analysis of AGP was close to this value (i.e., 0.899) (Supplementary Fig. 3). Here, we assumed that the *N-*glycopeptides matched against GPA-DB-AGP represented true assignments if they were present in the reference list of Ozohanics *et al.*^28^ Fig. 2b (middle) shows the distribution of S-scores among 1,674 precursor ions with M-score ≥ 1.3 in the analysis described above. We manually confirmed those spectra contained 924 precursor ions of *N-*glycopeptide candidates with S-score ≥ 98 (FDR of 19.7%, Fig. 2b, right). Of these, 195 were found in the reference list of AGP (Estimated FDR of 14.7%).

### Identification of *N-*glycopeptides using Y-score with FDR

CID-MS/MS spectra of *N-*glycopeptides exhibit specific spectral characteristics: Y-ions, intact peptide ions with partially fragmented glycan moiety attached, and B-ions (multi-mono-saccharide fragments of non-reducing end of the attached glycan (Fig. 2c, left). For example, the precursor of ENGTISR_5402 (+3) glycopeptide from AGP are fragmented into the Y-ions (+2), Y-ions (+1) and B-ions (+1). (Here, the string of digits following the amino-acid sequence of the peptide denotes the composition of the attached glycan: for example, the glycoform with 5 Hex, 4 HexNAc, 0 Fucose, and 2 NeuAc, in that order, was designated 5402). HCD-MS/MS spectra of *N-*glycopeptides exhibit only Y-ions (+1) regardless of precursor ion charge, and y- and b-ions, which reveal the amino-acid sequences of peptides, as well as oxonium ions. We compared experimental CID- and HCD-MS/MS spectra to the theoretical ones expected from the *N-*glycopeptide candidates selected by S-scoring. Then CID_match_ and HCD_match_, which represent the matches between the experimental and theoretical CID and HCD fragment peaks of *N-*glycopeptides, respectively, were calculated according to Equation 3 (Supplementary Note 3 and 4).


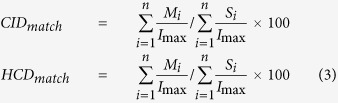


where n is the number of peaks, I_max_ is the intensity of the highest peak in the spectrum, M_i_ is the matched peak intensity, and S_i_ is the individual peak intensity in the spectrum. We combined CID_match_ and HCD_match_ to select as many true identifications of *N-*glycopeptide as possible. Eventually, the CID_match_:HCD_match_ ratio of 7:3 yielded the highest AUC value (Supplementary Fig. 4). Therefore we defined the Y-score according to Equation 4.


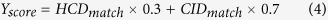


In order to estimate the number of false positive identifications, we calculated the FDR using a decoy method: After S-scoring, we obtained *N-*glycopeptide candidates, including their glycoforms and peptides and then made decoy B-and Y-ions candidates by changing the numbers of hexosamine (Hex), N-acetyl hexosamine (HexNAc), Fucose (Fuc), and N-acetylneuraminic acid (NeuAc) for glycoforms and amino-acid sequences of peptides. Based on these information, we constructed a decoy MS/MS database by exchanging the numbers of 1) Hex into HexNAc and HexNAc into Hex 2) Fuc into NeuAc and NeuAc into Fuc, and reverse 3) the amino acid sequence. As an example, we listed up the calculated B-, Y-ions, and their corresponding decoy ions from *N-*glycopeptide of ENGTISR_5402 (+3). (Supplementary Table 3).

Using this decoy MS/MS database, we obtained a Y-score distribution that enabled us to distinguish between false and true identifications (Fig. 2c, middle). According to this distribution, the Y-score ≥69.5 was determined for the selection of true identifications at an estimated FDR of 0.9%. Eventually we manually confirmed those 456 *N*-glycopeptide spectra from 95 unique *N-*glycopeptides at Y-score
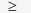
69.5 with 0% false positives in the analysis of the AGP standard sample (Fig. 2c, right, Supplementary Table 8, Supplementary Excel 4, and Supplementary PDF 1). We then validated the identification of *N-*glycopeptides by id-GPA with GPA DBs of various sizes from the standard AGP to mixture samples. (Supplementary Note 5, Supplementary Fig. 5, Supplementary Tables 4–6, and Supplementary Excels 5,6–7).

### High-speed label-free quantitation using q-GPA algorithm

**Figure 3:**
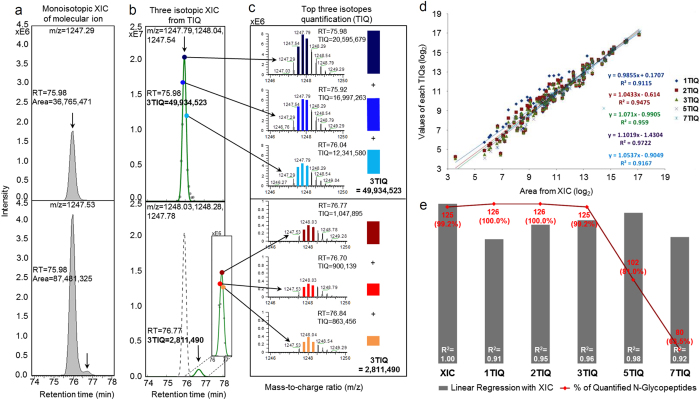
q-GPA algorithm for label-free quantitation of standard α1-acid glycoprotein (AGP). (**a**) The identified *N-*glycopeptides of AGP were quantitated based on the combined intensity of top three isotope peaks. For example, if we suppose to have two *N-*glycopeptides (#1 = CANLVPVPITNATLDQITGK_6503(+4) and #2 = CANLVPVPITNATLDQITGK_6522(+4)) with monoisotope mass of 1247.288 and 1247.533 Da, respectively, they might be distinguished in Fig. 3a (top) by MS due to their mass difference over 150 ppm. However, *N-*glycopeptide #2 in XIC (m/z = 1247.533) of Fig. 3a (bottom) can be interferenced by second isotope (m/z = 1247.538) of *N-*glycopeptide #1 due to their mass difference of 3–4 ppm. For correct quantification of *N-*glycopeptide #2, we introduced TIQ (Top Three Isotopes Quantification) method as shown in Fig. 3b,c, where 3TIQ uses the combined intensity of top three isotope peaks at three highest MS spectral points (Fig. 3b,c). Since we are evaluating the spectral pattern of selected MS spectra with S-score more than 98.0, it is possible to remove signal interference effectively from co-eluted peaks from similar m/z ions as shown in Fig. 3b, even though it has only mass difference of 3–4 ppm between the monoisotope ion of *N-*glycopeptide #2 and second isotope ion of *N-*glycopeptide #1. (**d**) Number of MS spectral points (1, 2, 3, 5, and 7) needed for TIQ, compared with the XIC manually extracted for quantitation. N is the number of selected *N*-glycopeptides used for quantitation of the AGP standard. (**e**) Gray bars indicate linear regression with XIC quantitation. Red line indicates the percentage of identified *N*-glycopeptides that were quantitated. The highest number of *N*-glycopeptides was quantitated by 3TIQ, which yielded the best linear regression (R^2^ = 0.959) with XIC.

For automated label-free quantitation of the identified *N-*glycopeptides, we developed q-GPA using a new algorithm, named top three-isotopes quantification (TIQ). 3TIQ uses the combined intensity of top three isotope peaks at three highest MS spectral points (Fig. 3). This approach has several advantages. First, because it requires no peak area generation from the extracted ion chromatogram (XIC), it allows high-speed quantitation. Second, as in the case of evaluating the isotope pattern by S-scoring, we effectively remove signal interference from co-eluted ions of similar m/z values. Third, considering top three isotope peaks provides more sensitive results with better S/N ratios, because for *N-*glycopeptides ( > 4,000 Da) the M + 1, M + 2 and M + 3 isotope peaks are generally more intense than the M peak. To determine how many MS data points are needed for 3TIQ based quantitation, we quantitated *N-*glycopeptides according to the number of MS spectral points and compared the results (Fig. 3d, Supplementary Excel 8). Considering the R^2^ ≥ 0.95 with XIC and % of quantitated *N-*glycopeptides ≥ 99.0%, we found that three data points with the highest values gave the best results (Fig. 3e). Overall, 99.2% of the identified *N-*glycopeptides were quantitated with a correlation of R^2^ = 0.959 against the XIC. Label free quantification by 3TIQ was validated with standard RNase B spiked at different concentrations in AGP standard solution and calibration curves of all *N-*glycopeptides exhibited good linearity (R^2^ ≥ 0.99). (Supplementary Note 5, Supplementary Fig. 6, and Supplementary Table 7).

### Comparative analysis of multiple samples using c-GPA algorithm

**Figure 4:**
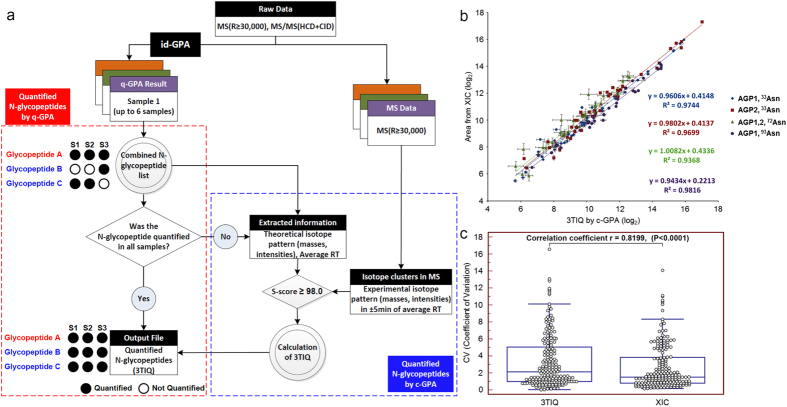
c-GPA algorithm for quantitation of three different HILIC-enriched batches of standard α1-acid glycoprotein (AGP). (**a**) Schematic algorithm of c-GPA for quantitation of *N-*glycopeptides from multiple samples. (**b**) Correlation coefficient of linear regressions (R^2^) between areas of manual 3TIQ and XIC were above 0.93 for several *N-*glycopeptides from an AGP standard sample enriched by HILIC. (**c**) The Pearson correlation coefficient (r) of a scatter graph of the coefficients of variation (CVs) was the value r = 0.8199, indicating the similarity between the results obtained by 3TIQ and XIC. The correlation coefficient of the two methods had a p-value below 0.0001.

After quantitation of *N-*glycopeptides in each sample using q-GPA, we performed comparative analysis using c-GPA. First, we combined all q-GPA data from multiple samples and compiled a total *N-*glycopeptide list that included information about isotope pattern, retention time, and abundance. As shown in Fig. 4a, if *N-*glycopeptide A were quantitated in all samples, we used their abundances obtained by q-GPA without additional processing. However, if *N-*glycopeptide B and C were not identified in some samples, as determined from the isotope pattern and retention time in the total *N-*glycopeptide list, we obtained the abundances of the corresponding *N-*glycopeptides using the 3TIQ method. Here, the similarity of the isotope pattern should be reflected by an S-score ≥ 98, with a retention time within 5 min of the previously observed one. In comparison with the abundances obtained from the XIC (Fig. 4b, Supplementary Excel 9), all *N-*glycopeptides at four N-glycosylation sites exhibited a good correlation (R^2^ > 0.93). The Pearson correlation coefficients on the coefficients of variation (CVs) (r = 0.8199, P < 0.0001) also demonstrated the similarity between the quantitative results obtained by c-GPA and conventional XIC (Fig. 4c). We evaluated the reproducibility in biological and technical replicates with benchmark datasets of standard glycoproteins spiked into HeLa cell lysates. (Supplementary Note 6 and Supplementary Fig. 7).

### Application of I-GPA to analysis of *N-*glycopeptides in the HCC plasma

To analyze *N-*glycopeptides that might be differentially expressed in HCC plasma using I-GPA platform, ten plasma samples from normal individuals, from which six major plasma proteins were either depleted or not depleted, were pooled (Supplementary Note 7). Twelve nano-LC/MS runs were analyzed by I-GPA (Supplementary Fig. 8). Table 1 presents all the results, including the numbers of *N-*glycopeptide spectra selected by M-score, candidates selected by S-score, and *N-*glycopeptides identified by Y-score at an FDR of ≤1%. More *N-*glycopeptide related data were obtained from a comparative analysis of depleted and non-depleted plasma. All analyses exhibited similar changes in the numbers of spectra and peptides: ~50% of *N-*glycopeptide candidates selected by M-score were filtered out by S-score, ~20% of *N-*glycopeptide spectra were identified as *N-*glycopeptides, and half of those were ultimately characterized as unique site-specific *N-*glycopeptides.

**Figure 5:**
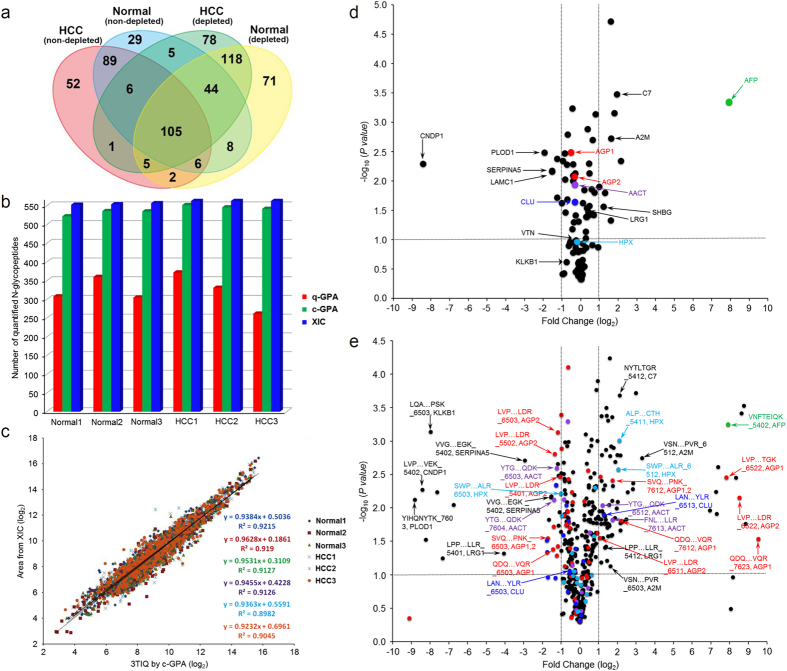
Analysis of *N-*glycopeptides in normal and hepatocellular carcinoma (HCC) plasma samples by I-GPA. (**a**) Venn diagrams of the number of unique *N-*glycopeptides identified in depleted or non-depleted samples of human plasma. (**b**) Results of label-free quantitative analysis of *N-*glycopeptides, using c-GPA on a depleted human plasma sample. (**c**) Comparison of label-free quantitation using 3TIQ on depleted human plasma after *N-*glycopeptides with coefficients of variation >30% were filtered out. (**d**,**e**) Volcano plot showing log (fold change) versus log (P-value) of differentially expressed glycoproteins (**d**) or *N-*glycopeptides (**e**) differentially expressed *N-*glycopeptides from AGP, AACT, HPX, CLU and AFP in red, purple, cyan, blue and green circle, respectively.

Our method identified 123 N-glycoproteins present in plasma at concentrations spanning five orders of magnitude, ranging from highly abundant proteins such as immunoglobulin G (IgG, ~1 mg/ml) to low-abundance proteins such as α-Fetoprotein (AFP, ~10 ng/ml). We also found a total of 619 unique *N-*glycopeptides (Fig. 5a): 449 and 352 *N-*glycopeptides, respectively, in normal and HCC plasmas from which six proteins were or were not depleted (Supplementary Fig. 9,10, Supplementary Excels 10,11).

For quantitative analysis, the abundance of *N-*glycopeptides obtained by the 3TIQ method was globally normalized and compared using c-GPA. Among 566 *N-*glycopeptides identified from six experiments with depleted plasma, the average 549 XICs and 529 c-GPAs were compared (Fig. 5b, Supplementary Excels 12,13–14). Among 440 *N-*glycopeptides identified from six experiments with non-depleted plasma, the average 423 XICs and 404 c-GPA were compared (Supplementary Fig. 11, Supplementary Excels 15,16–17). Figure 5c shows that the abundances obtained by conventional XIC and c-GPA based on the 3TIQ method were very similar. In c-GPA, the abundances of *N-*glycopeptides were averaged when they were quantitated two or more times by 3TIQ in three experiments. Nineteen *N-*glycopeptides with CV ≥ 30% were excluded. In cases, where the same *N-*glycopeptides were yielded at different numbers of charged ions, the results were combined. A total of 435 and 342 unique site-specific *N-*glycopeptides from depleted and non-depleted plasma, respectively, were quantitatively compared. Collectively, we were able to automatically identify 619 site-specific *N-*glycopeptides with FDR ≤ 1% and simultaneously quantitate 598 *N-*glycopeptides from human plasma.

### Statistical analysis of the human plasma glycoproteome using a volcano plot

Based on a quantitative comparison of site-specific *N-*glycopeptides, we performed a statistical analysis of the human plasma glycoproteome using a volcano plot. Figure 5d shows the fold changes calculated by abundances of N-glycoproteins in normal and HCC plasma. The abundance of each N-glycoprotein was determined by summing the abundances of all site-specific *N-*glycopeptides used for identification of that N-glycoprotein. Fourteen N-glycoproteins, including AFP, exhibited >2-fold differences in abundance in HCC relative to normal plasma. We found that the *N-*glycopeptide VNFTEIQK_5402 from AFP (Supplementary Fig. 12). It is a well-known liver cancer marker currently in clinical use (green circle in Fig. 5d), was only represented in depleted HCC plasma.

Several differentially presented glycoproteins in HCC plasma that were previously reported as candidate cancer biomarkers exhibited the same tendency in our analysis: levels of α2-macroglobulin (A2M)^31^,^32^, sex hormone–binding globulin (SHBG)^31^,^33^, and complement component C7^34^,^35^ were elevated in HCC plasma, whereas levels of SERPINA5^35^ and laminin (LAMC1)^36^,^37^ were reduced. However, most N-glycoproteins including clusterin (CLU, blue circle in Fig. 5d) reported as HCC markers^38^ showed no significant fold changes.

As our approach can identify specific changes in each *N-*glycopeptide of a single glycoprotein, we performed the same statistical analysis on site-specific *N-*glycopeptides (Fig. 5e). The results revealed 110 site-specific *N-*glycopeptides exhibiting >2-fold differences in abundance in HCC versus normal plasma. This number was much larger than the analogous value obtained at the glycoprotein level (i.e., 32 and 78 *N-*glycopeptides present at high levels in normal and HCC plasma, respectively). Some *N-*glycopeptides exhibited different fold changes according to glycoforms attached at a given glycosylation site. In addition, 69% of the 78 *N-*glycopeptides that were elevated in HCC plasma contain more than one fucose.

Typically, N-glycoproteins exhibited significant changes not at the glycoprotein level but at the *N-*glycopeptide level, as in the cases of AGP (red circle in Fig. 5e and Supplementary PDF 2), α1-antichymotrypsin (AACT, purple circle in Fig. 5e) and Hemopexin (HPX, cyan circle in Fig. 5e) in depleted plasma, and IgG (Supplementary PDF 3) in non-depleted plasma. Due to averaging effects at the protein level, these specific differences could not be detected without site-specific *N-*glycopeptide analysis. We examined site-specific N-glycosylation microheterogeneity in detail for each individual N-glycoprotein. The relative abundances of all site-specific *N-*glycopeptides identified in a single N-glycoprotein (IgG, AGP, or AACT) were compared between normal and HCC plasma (Supplementary Note 8 and Supplementary Fig. 13). These observations suggested that HCC was closely associated with fucosylation on branched glycoforms such as tri-antennary glycoforms. It is also consistent with the previous reports, regarding AGP^39^ and Hemopexin^40^, where hyper fucosylation and increased branching appear in liver diseases^41^.

## Discussion

In this study, we describe the I-GPA platform for high-throughput glycoproteomics and demonstrate the efficacy of this approach in an analysis of the glycoproteome of human plasma. I-GPA has several advantages. First, using the GPA-DB we constructed, id-GPA can directly identify site-specific *N-*glycopeptides from complex N-glycoprotein mixtures in plasma. The GPA-DB consists of intact *N-*glycopeptides produced by in silico trypsin digestion, including information about isotope mass and intensity, and can be freely expanded as required according to the sample. Second, id-GPA can calculate the FDR based on a decoy method to ‘tune’ the method to detect true matches. Third, searching by id-GPA is fast, because it works with only qualified mass data; unsatisfactory mass data with low scores are filtered out before the subsequent search step. Fourth, q-GPA can easily quantitate *N-*glycopeptides by the 3TIQ method in a label-free manner, using the peak intensities of the major isotope ions rather than the peak area. In addition, it does not require generation of peak areas from the extracted ion chromatogram. Previous methods for comparative analysis were time-consuming and laborious because they were generally performed by chromatography of monoisotope ions, manually extracted from LC-MS/MS analyses within limited MS tolerances and retention time windows. By contrast, q-GPA enables rapid quantitative analysis. Finally, I-GPA supports a variety of high-resolution MS equipment (Rs ≥ 30,000) with MS/MS fragmentation, including LTQ-FT, Orbitrap, and Q-Tof. For example, the identification of *N-*glycopeptides from standard AGP using id-GPA search in Orbitrap and QTOF MS analysis gave almost similar results at estimated FDR ≤ 1% (Supplementary Fig. 14, Supplementary Note 9, Supplementary Excel 5 and 18, Supplementary PDF 4).

Taken together, I-GPA can serve as a new versatile search engine for automated analysis of complex standard glycoproteins as well as biological/clinical samples. In the comparison of I-GPA and commercial software (Byonic) for the analysis of standard AGP, Byonic gave 27.5% false positives at an FDR of 0% as the tool offered. On the other hand, I-GPA gave 95 site-specific *N-*glycopeptides from standard AGP sample were identified with 0% false positives at an estimated FDR ≤ 1% using GPA decoy method (Supplementary Note 10, Supplementary Figs 15–17, Supplementary Table 8, Supplementary Excel 4). For potential use of this I-GPA platform in the biomarker discovery field, we applied our strategy to the analysis of N-glycoproteins present in human plasma, a representative bio-fluid containing a mixture of various glycoproteins. An automatic spectrum annotation of identified 619 site-specific N-glycopeptides with estimated FDR ≤ 1% from 123 glycoproteins marks the largest number reported to date, spanning five orders of magnitude in concentration and simultaneously quantifying 598 N-glycopeptides, from human plasma sample that are known to contain highly glycosylated proteins (Supplementary Figs 18,19, Supplementary PDF 5).

Our mapping performance was proven to be superior to the work recently reported by Mayampurath *et al.*^19^,^42^, where only 103 *N-*glycopeptides (FDR < 5%) with manual quantification of 40 *N-*glycopeptides by label-free mass analysis of human sera. Furthermore, we confirmed the mapping performance of I-GPA for IgG molecule, a representative serological glycoprotein, can be quantified with a total of 46 site-specific *N-*glycopeptides that were from IgG glycoforms 1, 2, 3, and 4 in human plasma. However, a recently published study by Huffman, J.E. *et al.*^43^ identified only 16 *N-*glycopeptides from purified IgG glycoforms 1 and 2 (Supplementary Table 9). Remarkably, in the analysis of the site-specific *N-*glycopeptides, changes in fucosylation of highly branched glycoforms were frequently observed in HCC plasma proteins, making its potential utility in clinical diagnostic research.

Given a great deal of biological interest, many approaches for the analysis of glycoproteins to date shows some limitations: lack of integration of proteomics and glycomics, a relatively small set of targeted glycoproteins and insufficient glycoprotein DBs. However, I-GPA, a newly developed search engine, covers both areas (i.e., glycoproteomics) and allows direct analysis of site-specific *N-*glycopeptides from complex glycoprotein mixtures using the efficient glycoprotein DBs, where an analytical efficiency was similar to that currently available in proteomics with FDR ≤ 1%. By fulfilling an unmet need for an automated method for high-throughput glycoproteomic analysis of broad biological samples, I-GPA will also contribute to C-HPP which commonly carries out comprehensive in-depth studies on cells, tissues, organs, and biological fluids.

## Methods

### Materials

Glycoprotein standards (RNase B(source: bovine, Cat. No. R1153), α1-acid glycoprotein (source: human, Cat. No. G9885) and IgG (source: human, Cat. No. I4505), 1,4-dithiothreitol (DTT), iodoacetamide (IAA), trifluoroacetic acid (TFA) and formic acid (FA) were purchased from Sigma-Aldrich (St. Louis, MO). For glycopeptides enrichment, ZIC-HILIC kit (ProteoExtract® Glycopeptide Enrichment Kit) was from EMD Millipore (Cat. No. 72103-3). Trypsin Gold (mass Spectrometry grade, V5280) for protein digestion was obtained from Promega (Madison, WI). HPLC grade acetonitrile from J.T. Baker (Phillipsburg, NJ) and deionized water through Millipore (Milli-Q Advantage A10 System) system were used. Plasma samples collected with an appropriate concentration of K_2_EDTA were obtained from Yonsei University College of Medicine (Seoul, Korea) along with IRB guideline for informed consent and approval and stored at −80 °C until use.

### Enzyme digestion of standard and plasma samples

RNase B, AGP and IgG, each glycoprotein standard solution was prepared at concentrations of 50 μg/100 μL in 20 mM Tris-HCl, pH 8.00 was denatured at 95 °C for 5 min. The protein solution cooled at room temperature was reduced by adding 2.5 μL of 200 mM DTT at 60 °C for 45 min and alkylated by adding 10 μL of 200 mM IAA at room temperature for 45 min (in the dark). 5 μL of 200 mM DTT was added and incubated at room temperature during 30 min for alkylation quenching. This solution was incubated with trypsin (total protein:trypsin = 10:1, by weight) at 37 °C overnight for digestion. For validation of M-, S- and Y-score in GPA algorithm, glycopeptides from digested AGP were concentrated by AmiconUltra 3 K MWCO (molecular weight cutoff) filters (product UFC500396; Millipore Ireland Ltd). For the calibration curves for N-linked glycopeptides from RNase B, six different concentrations of RNase B digest was each spiked in same amount AGP digest (0.15ug). For optimization of tandem mass spectrometry condition (CID and HCD), same amount of RNase B, AGP and IgG digests were combined. Digested samples were diluted with 0.1% FA/99.9 H_2_O for UPLC/LTQ-Orbitrap Elite mass spectrometry analysis or dried in a SpeedVac for glycopeptides enrichment

Multiple affinity removal column (MARC; Agilent) with HP1100LC system (Agilent) was used for the depletion of the six (albumin, transferrin, IgG, IgA, haptoglobin, and α_1_-antitrypsin) most abundant proteins in plasma according to the manufacturer’s specifications. Flow-through fractions that are “Depleted plasma” were collected and stored at −20 °C until use. Depleted plasma samples or non-depleted plasma samples were desalted and concentrated by centrifugal filtration using 10,000-Da MWCO (molecular weight cutoff) filter (VIVASPIN 6: product No. VS0602, Sartorius Stedim Biotech GmbH, Göttingen, Germany). The human plasma samples were quantitatively analyzed by Bradford protein assay. Ten individual plasma samples were respectively pooled for non-depleted normal and HCC, and depleted normal and HCC samples. Pooled plasma samples were diluted with 20 mM Tris-HCl buffer (pH 8.00). Diluted plasma samples (100 μg) were reduced, alkylated and digested with respectively DTT, IAA and trypsin such as above glycoprotein standard digestion protocol. Digested samples were diluted with 0.1% FA/99.9 H_2_O for UPLC/LTQ-Orbitrap Elite mass spectrometry analysis or dried in a SpeedVac for glycopeptides enrichment

### Enrichment of N-glycopeptides

Glycopeptides with ZIC-HILIC kit(ProteoExtract® Glycopeptide Enrichment Kit) was enriched according as the manufacturer’s processes (EMD Millipore). This kit includes ZIC^®^ Glycocapture Resin, ZIC^®^ Binding Buffer, ZIC^®^ Wash Buffer and ZIC^®^ Elution Buffer. Briefly, 10*μl*  AGP digest or real plasma digest of 2–4*μg* /*μl* concentration was prepared. 10 *μl* digested sample was diluted by adding 50 *μl* ZIC^®^ Binding Buffer. ZIC^®^ Glycocapture Resin was mixed and then, 50 *μl* homogenous ZIC^®^ Glycocapture Resin was taken to a new microcentrifuge tube. The tube that contains 50 *μl* homogenous ZIC^®^ Glycocapture Resin was centrifuged for 1–2 min at 2,000–2,500 × g. The supernatant was thoroughly removed and discarded. The diluted digest sample was transferred to the ZIC^®^ Glycocapture Resin, mixed by pipetting up and down, and incubated at 1,200 rpm for 10–20 min. The tube was centrifuged for 1–2 min at 2,000–2,500 × g. The supernatant was thoroughly removed and discarded. The ZIC^®^ Glycocapture Resin was washed with 150 *μl* ZIC^®^ Wash Buffer, mixed by pipetting up and down, and incubated at 1,200 rpm for 5–10 min. The tube was centrifuged for 1–2 min at 2,000–2,500 × g. The supernatant in the tube was thoroughly removed and discarded. The ZIC^®^ Glycocapture Resin was totally washed three times. 75–100 *μl* ZIC^®^ Elution Buffer was added for glycopeptides elution. The tube was mixed by pipetting up and down, incubated at 1,200 rpm for 2–5 min and centrifuged for 1–2 min at 2,000–2,500 × g. The supernatant which contains glycopeptides was transferred in a new microcentrifuge tube. The new microcentrifuge tube including glycopeptides was again centrifuged for 2 min at 10,000 × g and transferred in a new microcentrifuge tube to avoid the transfer of resin particles. Elutions were dried in SpeedVac and redissolved in 0.1% FA/99.9 H_2_O for UPLC/LTQ-Orbitrap Elite mass spectrometry analysis.

### Nano-LC-ESI-MS/MS analysis

Resolved or diluted samples with 0.1% FA/99.9 H_2_O were separated a Nano Acquity UPLC system (Waters, USA) and measured by an LTQ Orbitrap Elite mass spectrometer (Thermo Scientific, USA) equipped with a nano-electrospray source. An autosampler was used to load Each 5-μL aliquot of the peptide solution was loaded into a C_18_ trap-column of i.d. 180 μm, length 20 mm, and particle size of 5 μm (Waters, USA) with an autosampler. The peptides were desalted and concentrated on the trap column for 10 min at a 5 μL/min flow rate. Then, the trapped peptides were back-flushed on a homemade microcapillary column (i.d. 100 μm and length 200 mm, C_18_ of 3 μm particle size −125Å) for separation. Mobile phase A and B were composed with 100% water contained 0.1% formic acid and 100% acetonitrile (ACN) (B) contained 0.1% formic acid respectively. The LC gradient :5% B maintained from 0 to 15 min. Then, mobile phase B was ramped to 15% for 5 min, to 50% B for 75 min and to 95% B for 1 min. 95% B was remained for 13 min. B was decreased to 5% B for 1 min. The column was finally re-equilibrated with 5% B for 10 min. For plasma sample analysis, the LC gradient time was extended until 180 min. The voltage applied to produce the electrospray was 2.2 kV. The LTQ Orbitrap Elite mass spectrometry was operated in a data-dependent mode during the liquid chromatography separation. The MS acquisition parameters: resolution of full scans was 120,000 in Orbitrap for each sample; five data-dependent MS/MS scans were acquired by collision induced dissociation (CID) or(and) higher energy collision dissociation(HCD) per one full scan; CID scans and HCD scans were acquired in linear trap quadrupole (LTQ) with 30 ms activation time and were acquired in Orbitrap at resolution 15,000 with 20 ms activation time for each sample respectively; 35% normalized collision energy (NCE) in CID and HCD; 5.0 Da isolation window CID and HCD. Previously fragmented ions were excluded for 180 seconds for all MS/MS scans. The MS1 mass scan range was 400–2500 m/z for glycoprotein standard and 800–1800 m/z for plasma samples.

The condition of nano-LC-ESI-MS/MS for Q-TOF data : Digested AGP sample was separated by Ekspert™ nanoLC 400(Eksigent) and measured by an AB SCIEX TripleTOF® 5600^+^ mass spectrometer equipped with a nano-electrospray source in information-dependent acquisition (IDA) experiment mode. Sample was desalted and concentrated by a C_18_ trap-column (i.d. 180 μm, length 20 mm, and particle size 5 μm (Waters, USA)) for 10 min at a 5 μL/min flow rate. The trapped peptides were back-flushed on homemade microcapillary column (i.d. 100 μm and length 200 mm, C_18_ of 3 μm particle size −125Å) for separation. The LC gradient was performed for 120 min as same as that of nano-LC-ESI-MSMS for LTQ Orbitrap data acquisition. MS parameters were set to a MS1 scan of 250–1800 Da (250 msec accumulation time, positive ion mode) coupled to IDA criteria of a charge state of 2–5 exceeding 5 cps set to trigger a MS/MS product ion scan of 100–2000 Da (100 msec accumulation time, positive ion mode).

## Additional Information

**How to cite this article**: Park, G. W. *et al.* Integrated GlycoProteome Analyzer (I-GPA) for Automated Identification and Quantitation of Site-Specific N-Glycosylation. *Sci. Rep.* **6**, 21175; doi: 10.1038/srep21175 (2016).

## Supplementary Material

Supplementary Information

Supplementary Excel 1

Supplementary Excel 2

Supplementary Excel 3

Supplementary Excel 4

Supplementary Excel 5

Supplementary Excel 6

Supplementary Excel 7

Supplementary Excel 8

Supplementary Excel 9

Supplementary Excel 10

Supplementary Excel 11

Supplementary Excel 12

Supplementary Excel 13

Supplementary Excel 14

Supplementary Excel 15

Supplementary Excel 16

Supplementary Excel 17

Supplementary Excel 18

## Figures and Tables

**Table 1:** id-GPA search results (M-, S-, Y-score and FDR) by triplicate analysis of pools of normal and HCC human plasma.

Samples Sets		Number of N-glycopeptide spectra selected by M-score	M-score Thresholds	Number of N-glycopeptide candidates selected by S-score	S-score Thresholds	Number of N-glycopeptides identified by Y-score	Y-score Thresholds	Estimated FDR^a^ (%)	Number of unique N-glycopeptides	Number of unique glycoproteins
Non Depleted Human Plasma	Pooled Normal (10 cases)	3,296 (39.4%)	1.42	1,683 (51.1%)	98.0	412 (24.5%)	72.7	0.92	216	61
		3,013 (36.1%)	1.45	1,666 (55.3%)	98.0	467 (28.0%)	69.7	1.00	248	75
		3,163 (37.8%)	145	1,654 (52.3%)	98.0	443 (26.8%)	71.7	0.87	234	59
	Pooled HCC (10 cases)	2,623 (32.2%)	1.04	1,122 (42.8%)	98.0	254 (22.6%)	76.7	0.75	150	38
		2,829 (33.0%)	1.50	1,284 (45.4%)	98.0	304 (23.7%)	76.2	0.96	216	42
		2,345 (27.1%)	1.50	1,169 (49.8%)	98.0	425 (36.3%)	66.8	0.89	253	72
Top 6 Depleted Human Plasma^b^	Pooled Normal (10 cases)	5,281 (62.0%)	1.57	2,393 (45.3%)	98.0	508 (21.2%)	73.2	0.95	263	73
		5,422 (58.9%)	1.67	2,912 (53.7%)	98.0	612 (21.0%)	72.3	0.95	301	80
		5,703 (61.7%)	1.69	2,817 (49.4%)	98.0	497 (17.6%)	74.8	0.97	249	71
	Pooled HCC (10 cases)	5,564 (61.2%)	1.66	2,831 (50.9%)	98.0	628 (22.2%)	72.3	0.92	325	86
		5,175 (55.7%)	1.67	2,636 (50.9%)	98.0	592 (22.4%)	72.6	0.97	278	80
		5,302 (55.8%)	1.69	2,856 (53.9%)	98.0	556 (19.5%)	76.8	0.88	219	59

^a^Estimated FDR values calculated by GPA decoy method.

^b^The multiple affinity removal column (MARC; Agilent) was used to deplete the 6 most abundant human plasma proteins, namely, albumin, transferrin, IgG, IgA, haptoglobin, and α1-antitrypsin.
