# Supplementary material for: Integrated GlycoProteome Analyzer (I-GPA) for Automated Identification and Quantitation of Site-Specific N-Glycosylation
Source: Sci Rep. 2016 Feb 17;6:21175. doi: 10.1038/srep21175 (PMC4756296; doi:10.1038/srep21175)
Supplement: Supplementary Information [file srep21175-s1.pdf]

Supplementary information for:

Integrated GlycoProteome Analyzer (I-GPA) for Automated Identification and Quantitation of Site-Specific N-Glycosylation

Gun Wook Park*, Jin Young Kim*, Heeyoun Hwang, Ju Yeon Lee, Young Hee Ahn, Hyun Kyoung Lee, Eun Sun Ji, Kwang Hoe Kim, Hoi Keun Jeong, Ki Na Yun, Yong-Sam Kim, Jeong-Heon Ko, Hyun Joo An, Jae Han Kim, Young-Ki Paik , and Jong Shin Yoo**

*These authors are equally contributed.

**To whom correspondence should be addressed.

**Corresponding author: jongshin@kbsi.re.kr; Phone: +82 43 240 5150; Fax: +82 43 240 5159

List of supplementary notes, figures, tables, excels, and PDFs:

(1) List of supplementary notes:

Supplementary Note 1 GPA-DB construction using GPA-DB-Builder

Supplementary Note 2 RAW file conversion and search parameters for I-GPA

Supplementary Note 3 Calculation of M-score and Y-score

Supplementary Note 4 Generation of theoretical CID and HCD fragment peaks

Supplementary Note 5 Validation of id-GPA and q-GPA using standard samples

Supplementary Note 6 Quantification results for the proteome benchmark dataset

Supplementary Note 7 High-throughput global mapping of non-depleted and depleted plasma

Supplementary Note 8 Relative changes in the abundance of site-specific *N*-glycopeptides from IgG, AGP, and AACT in plasma

Supplementary Note 9 Identification of *N*-glycopeptides from standard α1-acid glycoprotein (AGP) using id-GPA search in Orbitrap and QTOF MS analysis.

Supplementary Note 10 Comparison between I-GPA and Byonic tools for the analysis of the standard α1-acid glycoprotein (AGP) data.

(2) List of supplementary figures:

Supplementary Figure 1 Criteria for the construction of GPA-DB-HumanPlasma, consisting of 291 selected glycoproteins from the PeptideAtlas library.

Supplementary Figure 2 Distributions of M-score of the HCD-MS/MS spectra.

Supplementary Figure 3 Comparison of the Area Under ROC Curves (AUC) for S-score optimization.

Supplementary Figure 4 Comparison of the Area Under ROC Curves (AUC) for the optimization of Y-scoring.

Supplementary Figure 5 Determination of estimated FDR (false-discovery rate) by GPA decoy method using 3 different GPA-databases

Supplementary Figure 6 Calibration curves of five *N*-glycopeptides from RNase B, quantitated by 3TIQ.

Supplementary Figure 7 Chromatograms from 12 human plasma samples.

Supplementary Figure 8 Venn diagrams of the number of *N*-glycoproteins and *N*-glycopeptides identified in 6 protein depleted and non-depleted plasma.

Supplementary Figure 9 Venn diagrams of the number of analyzed *N*-glycoproteins and *N*-glycopeptides identified in HCC and normal human plasma.

Supplementary Figure 10 Analysis of normal and HCC non-depleted plasma samples by I-GPA.

Supplementary Figure 11 Automated identification of site-specific *N*-glycopeptides from α-Fetoprotein (AFP) in HCC human plasma.

Supplementary Figure 12 Comparison of relative changes in the abundance of site-specific *N*-glycopeptides in normal and HCC human plasma.

Supplementary Figure 13 Label-free quantitative analysis of depleted normal and HCC plasma samples using I-GPA.

Supplementary Figure 14 Label-free quantitative analysis of non-depleted normal and HCC plasma samples using I-GPA.

Supplementary Figure 15 Identification of *N*-glycopeptides from standard α1-acid glycoprotein (AGP) using id-GPA search in Orbitrap and QTOF MS analysis.

Supplementary Figure 16 The scatter plots (a,c) of manually validated data in the analysis of standard α1-acid glycoprotein (AGP) and their receiver operating characteristic (ROC) curves (b,d) were compared between I-GPA and Byonic tools, respectively.

Supplementary Figure 17 Exemplary spectrum of false positive assignments by Byonic in the analysis of *N*-glycopeptides from standard α1-acid glycoprotein (AGP).

Supplementary Figure 18 Exemplary spectrum of false positive assignments by Byonic in the analysis of *N*-glycopeptides from standard α1-acid glycoprotein (AGP).

(3) List of supplementary tables:

Supplementary Table 1 List of tryptic peptides used for construction of GPA-DB-AGP and GPA-DB-Mixture.

Supplementary Table 2 Efficiency of HILIC enrichment for the identification of *N*-glycopeptides from α1-acid glycoprotein (AGP) standard sample.

Supplementary Table 3 Examples of target and decoy fragment ions (Y/B) generated from tryptic *N*-glycopeptide (ENGTISR_5402) of α1-acid glycoprotein (AGP).

Supplementary Table 4 Evaluation of FDR with GPA decoy method using different sizes of GPA-databases for the analysis of standard α1-acid glycoprotein (AGP).

Supplementary Table 5 Comparison of α1-acid glycoprotein (AGP) *N*-glycopeptides identified using GPA-DB-AGP, GPA-DB-Mixture, and GPA-DB-HumanPlasma.

Supplementary Table 6 Comparisons of number of N-glycoproteins and *N*-glycopeptides identified from seven standard glycoproteins mixture by using different MS/MS fragmentation and databases of different sizes for the id-GPA search.

Supplementary Table 7 Results of label-free quantitation by 3TIQ for the calibration curve

of spiked RNase B.

Supplementary Table 8 Summary of the numbers of selected spectra, *N*-glycopeptides, estimated FDRs, and manually validated FDRs generated by I-GPA and Byonic for the standard α1-acid glycoprotein (AGP) data.

Supplementary Table 9 Comparison of *N*-glycopeptide profiles of IgG1 in the reference vs. our data.

(4) List of supplementary excels:

Supplementary Excel 1 List of 351 N-glycans for GPA-DataBase.

Supplementary Excel 2 List of 291 glycoproteins for GPA-DB-HumanPlasma.

Supplementary Excel 3 *N*-glycopeptide sequences for GPA-DB-HumanPlasma.

Supplementary Excel 4 Manual validation with *N*-glycopeptide spectrum matches (GSMs) in AGP standard sample using Byonic and I-GPA.

Supplementary Excel 5 Identification of *N*-glycopeptides in α1-acid glycoprotein (AGP) standard sample without HILIC enrichment.

Supplementary Excel 6 Identification of *N*-glycopeptides in standard mixture sample using GPA-DB-Mixture.

Supplementary Excel 7 Identification of *N*-glycopeptides in standard mixture sample using GPA-DB-HumanPlasma.

Supplementary Excel 8 Quantification of *N*-glycopeptides in α1-acid glycoprotein (AGP) standard sample without HILIC enrichment.

Supplementary Excel 9 Quantification of *N*-glycopeptides in α1-acid glycoprotein (AGP) standard samples with HILIC enrichment.

Supplementary Excel 10 Identification of *N*-glycopeptides in depleted human plasma.

Supplementary Excel 11 Identification of *N*-glycopeptides in non-depleted human plasma.

Supplementary Excel 12 Quantification of *N*-glycopeptides in depleted human plasma.

Supplementary Excel 13 Quantification of *N*-glycoproteins in depleted human plasma.

Supplementary Excel 14 Quantification of *N*-glycans in depleted human plasma.

Supplementary Excel 15 Quantification of *N*-glycopeptides in non-depleted human plasma.

Supplementary Excel 16 Quantification of *N*-glycoproteins in non-depleted human plasma.

Supplementary Excel 17 Quantification of *N*-glycans in non-depleted human plasma.

Supplementary Excel 18 Identification of *N*-glycopeptides in α1-acid glycoprotein (AGP) standard sample by QTOF MS.

(5) List of supplementary PDFs:

Supplementary PDFs were linked to following sites due to their big data size.

Supplementary PDF 1 The spectra manually assigned as the *N*-glycopeptides from α1-acid glycoprotein (AGP) glycoprotein in standard by Orbitrap MS (The Supplementary PDF 1 file is available for download at ftp://massive.ucsd.edu/MSV000079426/other/Supplementary_PDF_File1_Standard_AGP_SpectrumAssign_Orbitrap.pdf).

Supplementary PDF 2 The spectra manually assigned as the *N*-glycopeptides from α1-acid glycoprotein (AGP) glycoprotein in depleted plasma (The Supplementary PDF 2 file is available for download at ftp://massive.ucsd.edu/MSV000079426/other/Supplementary_PDF_File2_HumanPlasma_AGP_SpectrumAssign.pdf).

Supplementary PDF 3 The spectra manually assigned as the *N*-glycopeptides from IgG glycoprotein in non-depleted plasma (The Supplementary PDF 3 file is available for download at

ftp://massive.ucsd.edu/MSV000079426/other/Supplementary_PDF_File3_HumanPlasma_IgG_SpectrumAssign.pdf).

Supplementary PDF 4 The spectra manually assigned as the *N*-glycopeptides from α1-acid glycoprotein (AGP) glycoprotein in standard by QTOF MS (The Supplementary PDF 4 file is available for download at ftp://massive.ucsd.edu/MSV000079426/other/Supplementary_PDF_File4_Standard_AGP_SpectrumAssign_QTOF.pdf).

Supplementary PDF 5 The spectra automatically assigned as the 619 N-glycopeptides from 123 glycoproteins in human plasma (The Supplementary PDF 5 file is available for download at ftp://massive.ucsd.edu/MSV000079426/other/Supplementary_PDF_File5_HumanPlasma_619Glycopeptides_SpectrumAssign.pdf).

Supplementary Notes

Supplementary Note 1: GPA-DB construction using GPA-DB-Builder

The 351 N-glycans downloaded from Ozohanics, O et al.[1] for the mammalian *N*-glycopeptide database (**Supplementary Excel 1**). Amino acid sequence and N-glycosylation site information were obtained from the UniProt database. From the UniProt accession number, GPA-DB-Builder program automatically performs in silico trypsin digestion (up to 2 missed cleavages). Information on glycoprotein, peptide sequence, and N-glycosylation site information were stored in the Excel file, and the isotope pattern (masses and relative intensities) of *N*-glycopeptide (peptide+glycan) is calculated. GPA databases were constructed for individual standard samples. The α1-acid glycoprotein (AGP) study used a database (GPA-DB-AGP) constructed from AGP 1 and 2 glycoproteins (**Supplementary Table 1**). The mixed sample study used a database (GPA-DB-Mixture) constructed from the UniProt database containing the three model glycoproteins, AGP, IgG, and RNase B (**Supplementary Table 1**). GPA databases were constructed by GPA-DB-Builder according to the sample including standard glycoproteins and human plasma. The human glycopeptide database was constructed as a TXT file, combining possible tryptic peptides and glycans for 291 glycoproteins in human plasma (**Supplementary Excel 2**) selected from the PeptideAtlas library. It includes a total of 722 *N*-glycosylation sites and 726 tryptic *N*-glycopeptide sequences for 282 known plasma glycoproteins (**Supplementary Fig. 1 and Supplementary Excel 3**).

Supplementary Note 2: RAW file conversion and search parameters for I-GPA

The mass spectrometer RAW file from Orbitrap was converted to MS (.txt) and MS/MS (.mgf) files using the freeware program RawExtractor v1.9 (The Scripps Research Institute, La Jolla, CA) and MM File Conversion Tools v3.9 (http://www.massmatrix.net/mm-cgi/downloads.py). *N*-glycopeptides were analyzed by high resolution mass spectrometry with HCD, CID, and HCD/CID fragmentation followed by id-GPA, with following search parameters: glycopeptide tolerance = 10 ppm; Fragment tolerance = 0.02 Da (HCD) and 1.5 Da (CID); Missed cleavages = 0, Modification: Carbamidomethyl cysteine (fixed), N-glycan search (Mammalian). The I-GPA software is available for free download at https://drive.google.com/open?id=0ByH5JUI8j8UFfnBmbnJoRWJSTDgtVzZUUlZtd01QQ2NnOUxRNHE3ZVp6SnA0clJPd3hlR2M&authuser=0/IGPAv1.0_DataFiles.zip . For details on system requirements and input RAW file formats, see the READ-ME file on the Web site.

Supplementary Note 3: Calculation of M-score and Y-score

In order to calculate M-score as follows, our method uses total 15 oxonium ions (Fig. 2a left in the main text) differently weighted according to their frequency of appearance in HCD spectra[1] (For example of oxonium ions: weight of m/z 657 ion=1, weight of m/z 366 ion=3, weight of m/z 204 ion=4, weight of m/z 186 ion=4, weight of m/z 168 ion=4, weight of m/z 138 ion=4, weight of m/z 528 ion=1, weight of m/z 350 ion=1, weight of m/z 147 ion=1, weight of m/z 129 ion=1, weight of m/z 454 ion=1, weight of m/z 292 ion=2, weight of m/z 274 ion=2, weight of m/z 163 ion=4, and weight of m/z 145 ion=4). The weight value (C) indicates the number of arrow in Fig. 1a, which is from 1 to 4 corresponding to their theoretical frequency of appearance in HCD spectra (Equation 1 in the main text). According to Equation 1, we can

obtain M-score distribution from HCD-MS/MS spectra of our sample. M-score distribution can be little bit different between experiments. So, we made that M-score thresholds is automatically determined by Gaussian Fit to discriminate glycopeptides spectra from non-glycopeptides ones.

In order to determine Y-score threshold, we calculated the estimated FDR using a novel decoy method. After S-scoring, we obtained N-glycopeptide candidates including their glycoforms and peptides. Based on this information, we constructed a decoy MS/MS database by changing the numbers of Hex, HexNAc, Fuc, and NeuAc (for glycoforms) and amino-acid sequences (for peptides). Using this decoy MS/MS database, we obtained a decoy-score distribution that enabled us to distinguish between false and true identifications. From this distribution, the Y-score threshold was determined for the selection of true identifications with a given estimated FDR (Fig. 2c middle in the main text). Because we fixed 1% estimated FDR in every analysis Y-score thresholds can be different between experiments. Y-score threshold is automatically determined by target and decoy score distribution of each result.

Supplementary Note 4: Generation of theoretical CID and HCD fragment peaks

For generation of theoretical CID and HCD fragments (Y- and B- ions), we consider the theoretically possible 362 glycan fragments in GPA database. First of all, the molecular weights of 362 glycan fragments are added to that of peptide from N-glycopeptide candidate to generate the list of total Y-ions. Y-ions should have lower molecular weight than precursor ion, and their glycan type such as complex, high mannose and hybrid forms should be the same type as parents N-glycopeptides. In addition, there are glycan fragment ions such as 1_1_0_0 (366.14

Da), 1_0_0_1 (454.156 Da), 2_1_0_0 (528.1928 Da), 1_1_0_1 (657.235 Da), 2_1_0_1 (819.288 Da), 2_2_0_1 (1022.367Da), 1_1_2_0 (658.268Da), 1_1_1_1 (803.299Da), 1_1_2_1 (949.363Da) as B-ions. Their glycan type should have consensus with parents N-glycopeptides. In theoretical CID fragments, the charge of N-glycopeptide fragments (Y-ions) are considered from precursor charge -1 up to +1. For example, the precursor of ENGTISR_5_4_0_2 (+3) are fragmented into the double and single charged Y-ions and B-ions. In theoretical HCD fragments, only single charged Y-ions are considered regardless of precursor ion's charge. The theoretical fragments of b- and y-ions from peptide sequence are generated with the same method as the Mascot and Sequest programs do.

Supplementary Note 5: Validation of id-GPA and q-GPA using standard samples

The analysis of the AGP standard by id-GPA ultimately yielded 95 unique *N*-glycopeptides. (**Supplementary Excel 5, Supplementary PDF 1**). To validate the identification of *N*-glycopeptides by id-GPA, we performed the same analysis with GPA databases of various sizes. As shown in **Supplementary Table 4**, id-GPA yielded almost same results in terms of the numbers of selected *N*-glycopeptides spectra, selected *N*-glycopeptide candidates, and identified *N*-glycopeptides from AGP, with similar FDRs of ≤1.0% (**Supplementary Fig. 5**). Regardless of DB size, the analysis yielded consistent results: for GPA-DB-AGP, GPA-DB-Mixture, and GPA-DB-Human Plasma (ranging in size from 4,212 to 254,826 *N*-glycopeptides), respectively, we obtained 95, 95, and 93 unique *N*-glycopeptide assignments (**Supplementary Table 4**). Two *N*-glycopeptides, QDQCIYNTTYLNVQR_6501 and CANLVPVPITNATLDR_6503, yielded poor-quality MS/MS spectra; nonetheless, they were

misidentified only in a search of the largest database, GPA-DB-Human Plasma. (Here, the string of digits following the amino-acid sequence of the peptide denotes the composition of the attached glycan: for example, the glycoform with 6 Hex, 5 HexNAc, 0 Fucose, and 3NeuAc, in that order, was designated 6503).

We performed additional analysis using an N-glycoprotein standard mixture containing various types of *N*-glycopeptides, including glycoforms of the high-mannose type from RNase B and similar amino-acid sequences from IgG isoforms Again, the accuracy of our identification of *N*-glycopeptides was similar, regardless of the types of *N*-glycopeptides types and the size of the database. (**Supplementary Table 6** and **Supplementary Excels 6, 7**).

Using q-GPA, we determined calibration curves for the *N*-glycopeptides from glycoprotein RNase B, spiked at different concentrations into AGP standard solution (**Supplementary Fig. 6** and **Supplementary Table 7**). The levels of five *N*-glycopeptides increased along with the concentration of RNase B, with good linearity (R^2 = 0.99).

Supplementary Note 6: Quantification results for the proteome benchmark dataset

In order to evaluate the reproducibility in biological and technical replicates, we prepared a benchmark dataset with standard glycoproteins spiked into HeLa cell lysates at two known ratios. In sample 1, each 1ug of IgG, fetuin, vitronectin, haptoglobin AGP, and alpha-1-antitrypsin were spiked into 15ug of HeLa cell lysates. In sample 2, each 1ug of IgG, fetuin, vitronectin, haptoglobin and each 3ug of AGP, and Alpha-1-antitrypsin were spiked into 15ug of HeLa cell lysates. They were individually enriched by HILIC and followed by LC/MS/MS

analysis. As you can see in **Supplementary Fig. 7**, they were detected at two different ratios over the entire glycoprotein expression range.

Replicate samples were filtered for two out of three valid values and averaged. The log ratios of 3TIQ between the sample 1 and sample 2 of 1:1 (orange) versus 3:1 (blue) ratios were plotted against the logarithm of N-glycopeptide intensities (**Supplementary Fig. 7A**). They were completely distinguished across the entire abundance range. Comparison of technical replicates in both runs of sample 1 shows repeatability of runs by R2 = 0.9816 (**Supplementary Fig. 7B**). The histogram of the ratio distributions of HeLa + four standards (IgG, fetuin, vitronectin, and haptoglobin) and two standards (AGP and alpha-1-antitrypsin) N-glycopeptides were obtained using the 3TIQ method. **Supplementary Fig. 7C** showed logarithmic fold change of 1.56, which is close to the expected logarithmic fold change of 1.58, showing accurate relative abundance of N-glycopeptides at two known ratios (1:1 and 1:3). 3TIQ performed best by generating narrow distributions.

Supplementary Fig. 7D showed quantification results of benchmark dataset (sample 3 and sample 4) with two orders of magnitude in dynamic range. Sample 3 were prepared by spiking each 1ug of fetuin, IgG, A1AT, vitronectin, and haptoglobin into 20ug of HeLa cell lysates. Sample 4 were prepared by spiking 1ug of fetuin, each 10ug of IgG and A1AT, and each 0.1ug of vitronectin and haptoglobin into 20ug of HeLa cell lysates. The logarithm of experimental abundance ratio of glycopeptides obtained from 3TIQ method were well matched against the logarithm of true abundance ratio between sample 3 and sample 4 (sample 3 / sample 4 = 1 (fetuin), = 0.1 (IgG and A1AT), = 10 (vitronectin and haptoglobin)).

Supplementary Note 7: High-throughput global mapping of non-depleted and depleted plasma

Of these, 449 and 352 unique site-specific *N*-glycopeptides identified in non-depleted and depleted plasma were quantitatively compared between normal and HCC plasma (see **Supplementary Fig. 13 and Supplementary Excels 12–14** for depleted normal and HCC plasma, see **Supplementary Fig. 14** and **Supplementary Excels 15–17** for non-depleted normal and HCC plasma); proteins shown in red and in green were highly represented in HCC plasma and in normal plasma, respectively. Biantennary glycoforms (e.g., 5402) with the largest numbers of linkages were mostly detected in non-depleted and depleted human plasma.

In the case of depleted plasma, the *N*-glycopeptides (outer circle) were sorted into 99 N-glycoproteins (middle circle) and linked to 58 glycoforms (core circle) using Cytoscape[2,3] (**Supplementary Fig. 13**). Because even a single N-glycoprotein can have various glycoforms, and the same glycan can be found in various N-glycoproteins, multiple linkages were observed between glycoforms and N-glycoproteins. In another case of non-depleted plasma, the *N*-glycopeptides (outer circle) were sorted into 89 N-glycoproteins (middle circle) and linked to 66 glycoforms (core circle) (**Supplementary Fig. 14**).

Supplementary Note 8: Relative changes in the abundance of site-specific *N*-glycopeptides from IgG, AGP, and AACT in plasma

This global mapping permits the identification of proteins present at different concentrations, while our approach can identify the specific changes of each *N*-glycopeptide in a single protein. We examined site-specific N-glycosylation microheterogeneity in detail based on each single

N-glycoprotein identified here. Typically, some N-glycoproteins showed significant changes not at the protein level but at the *N*-glycopeptide level, such as IgG from non-depleted plasma, and AGP and A1ACT from depleted plasma. The relative abundances of all site-specific *N*-glycopeptides identified in a single N-glycoprotein were compared between normal and HCC plasma: IgG (**Supplementary Fig. 12a**), AGP (**Supplementary Fig. 12b**) and A1ACT (**Supplementary Fig. 12c**).

Supplementary Fig. 12a shows the *N*-glycopeptides from IgG according to the various site-specific glycoforms. IgG 1, 2 and/or 3, and 3 and/or 4 were individually identified by 46 site-specific *N*-glycopeptides, primarily the core-fucosylated bi-antennary glycoforms. A recently published study[4] was able to identify only 16 *N*-glycopeptides from IgG1 and 2 in 1,821 human plasma samples. By contrast, our method allowed quantitation of 46 *N*-glycopeptides without protein enrichment, and their percent of mono-galactosylated and di-galactosylated compositions were accurately identified (~95%) by our method (**Supplementary Table 9**). In HCC plasma, all IgG isomers exhibited markedly elevated agalactosylated glycan compositions relative to normal plasma, which was also consistent with the results of other studies of cancer-related changes in IgG glycosylation[5] (**Supplementary Fig. 12a**). In the case of AGP, we identified more *N*-glycopeptides with more highly branched complex glycoforms from AGP1 and AGP2 (**Supplementary Fig. 12b**). Of these, 92% (54/59) overlapped with *N*-glycopeptides identified from the standard samples. Notably, all *N*-glycopeptides present at high levels in HCC contained fucose in their glycoforms. AACT also followed the same trend, exhibiting larger changes in fucosylation on *N*-glycopeptides with more highly branched glycoforms (**Supplementary Fig. 12c**).

Supplementary Note 9: Identification of *N*-glycopeptides from standard α1-acid glycoprotein (AGP) using id-GPA search in Orbitrap and QTOF MS analysis.

In order to demonstrate the efficacy of id-GPA, we have tried careful manual curation for total 1,000 MS/MS spectra (**Supplementary Excel 4**) of standard AGP sample from Orbitrap and QTOF that have the capability to do CID and/or HCD. We consider the CID data with mass tolerance of 1.5 Da for Orbitrap and 0.02 Da for QTOF. Individual fragment ions (b-ions, y-ions, B-ions, and Y-ions) of 456 filtered MS/MS spectra of 95 unique *N*-glycopeptides from Orbitrap MS are assigned, where in the case of QTOF MS, the id-GPA gave 985 filtered MS/MS spectra of 111 unique *N*-glycopeptides identified at estimated FDR < 1% using GPA decoy method (**Supplementary Fig. 15**).

Supplementary Note 10: Comparison between I-GPA and Byonic tools for the analysis of the standard α1-acid glycoprotein (AGP) data.

In order to understand which tools have better performance, we tried to compare our I-GPA to the identical analysis using the newest version of Byonic (v2.0-25). Almost 1,000 MS/MS data of *N*-glycopeptides were reanalyzed on both I-GPA and Byonic. We compared our I-GPA to the Byonic software for the analysis of the standard AGP data. (**Supplementary Excel 4**) Byonic gave 28% false positives at an FDR of 0% as the Byonic tool offered. This might be comparable to the report that S. W. Wu, T. H. Pu, R. Viner et al.[6] found more than 37% false positives in the analysis of single EGFR protein despite a claim of zero FDR by using Byonic program[5]. On the other hand, I-GPA gave 0.0% false positives at an estimated FDR of 0.9%

using GPA decoy method (**Supplementary Table 8**). In the statistical analysis using the scatter plots and ROC curve for the manually validated data between two different algorithms, I-GPA (AUC=0.974) has showed much higher sensitivity and specificity than Byonic (AUC=0.726) (**Supplementary Fig. 16**).

For example, Byonic resulted in incorrect identification of *N*-glycopeptide because of poor assignment of fragment ions in CID MS/MS spectrum. And, its corresponding HCD MS/MS spectrum showed no identification (**Supplementary Fig. 17**). In CID MS/MS spectrum, the intense fragment ions presenting sequential glycosyl losses were not assigned by Byonic, while, they were clearly assigned by id-GPA (b-bottom) confirming it as *N*-glycopeptide (SVQ…PNK_6513). And b- and y ions by peptide backbone fragmentation were well matched in its corresponding HCD MS/MS spectrum. As an another example, Byonic resulted in incorrect identification of *N*-glycopeptide because of poor assignment of fragment ions in HCD MS/MS spectrum. And, its corresponding CID MS/MS spectrum showed no identification (**Supplementary Fig. 18**). In HCD MS/MS spectrum, only a few the fragment ions generating by sequential glycosyl losses were assigned by Byonic. It lead a false identification as LVPVPITNATLDR_6531 in glycoform attatched to peptides. However, id-GPA resulted in true identification by combined search of HCD and CID MS/MS spectrum, where CID MS/MS showed clearly the fragment ions generating by sequential glycosyl losses to correctly identify *N*-glycopeptides as LVPVPITNATLDR_6512 including glycoform.

References

1. Ozohanics, O., Turiak, L., Puerta, A., Vekey, K. & Drahos, L. High-performance liquid chromatography coupled to mass spectrometry methodology for analyzing site-specific N-glycosylation patterns. Journal of chromatography. A 1259, 200-212 (2012).

2. R. Saito, M. E. Smoot, K. Ono et al., Nature methods 9 (11), 1069 (2012).

3. M. S. Cline, M. Smoot, E. Cerami et al., Nature protocols 2 (10), 2366 (2007).

4. Huffman, J.E. et al. Comparative Performance of Four Methods for High-throughput Glycosylation Analysis of Immunoglobulin G in Genetic and Epidemiological Research. Molecular & cellular proteomics : MCP 13, 1598-1610 (2014).

5. Malhotra, R. et al. Glycosylation changes of IgG associated with rheumatoid arthritis can activate complement via the mannose-binding protein. Nature medicine 1, 237-243 (1995).

6. Wu, S.W., Pu, T.H., Viner, R. & Khoo, K.H. Novel LC-MS2 product dependent parallel data acquisition function and data analysis workflow for sequencing and identification of intact glycopeptides. Analytical chemistry 86, 5478-5486 (2014).

Supplementary Figures

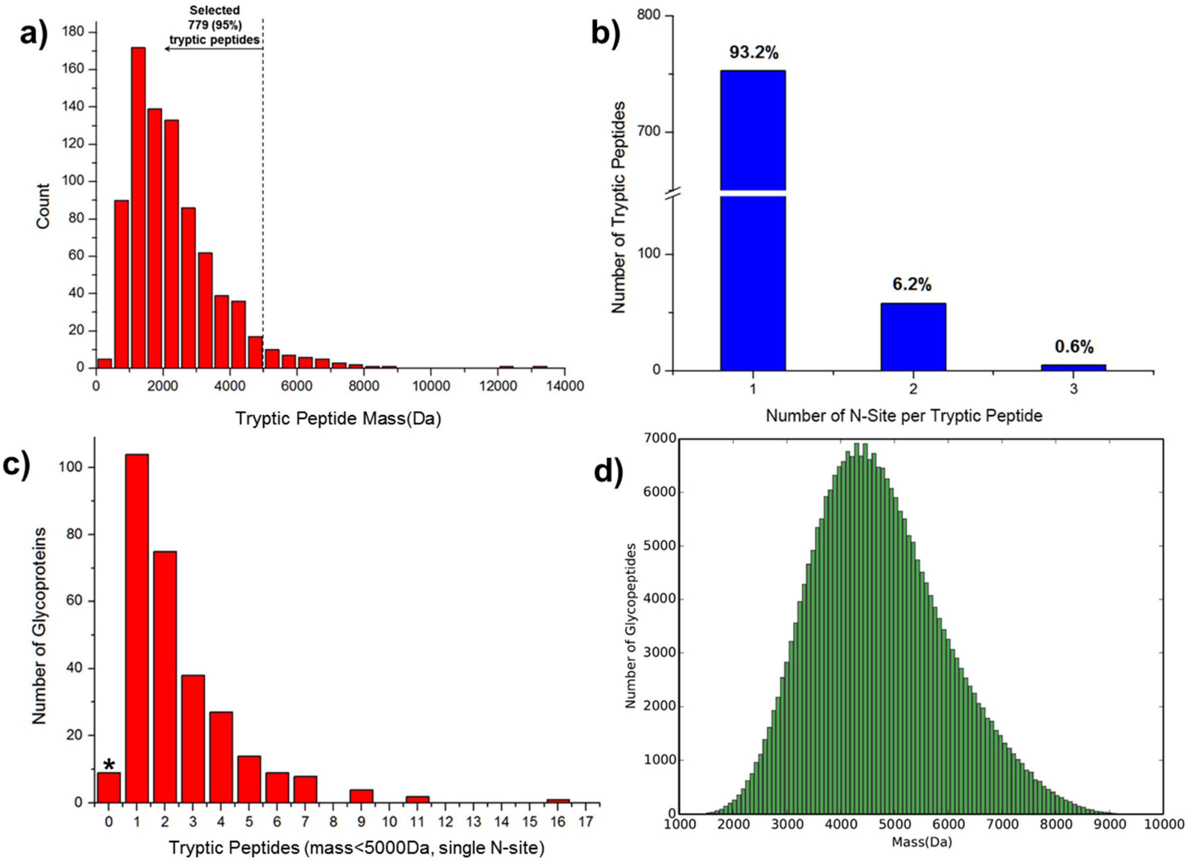

Supplementary Figure 1. Criteria for the construction of GPA-DB-Human Plasma, consisting of 291 selected glycoproteins from the PeptideAtlas library.

(a) The mass range is confined from 400 Da to 5000 Da for best performance of MS acquisition; therefore, 779 tryptic peptides (95%) were considered. (b) The distribution of the number of *N*-glycosylation sites in the UniProt database within a tryptic peptide backbone. Of the 779 peptides, 93.2% were singly *N*-glycosylated peptides, i.e., peptides with only a single *N*-glycosylation site. (c) Distribution of 722 tryptic peptides from 282 glycoproteins with 726 *N*-glycosylation sites (*9 glycoproteins were excluded because of the criteria described above: P07225, P12830, P17813, P12318, Q9BTY2, P00740, P10586, Q12841, P10153) (d) A total of 253,422 *N*-glycopeptides in the mass range 1000–10,000 Da.

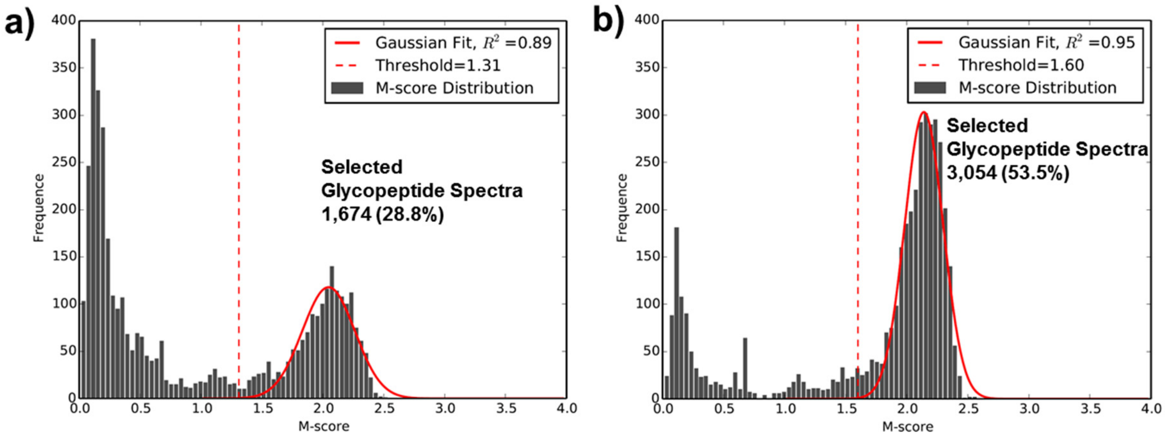

Supplementary Figure 2. Distributions of M-score of the HCD-MS/MS spectra.

The distributions of M-scores (red line) from tryptic *N*-glycopeptides in the analysis of α1-acid glycoprotein (AGP) standard sample (a) without and (b) with HILIC enrichment.

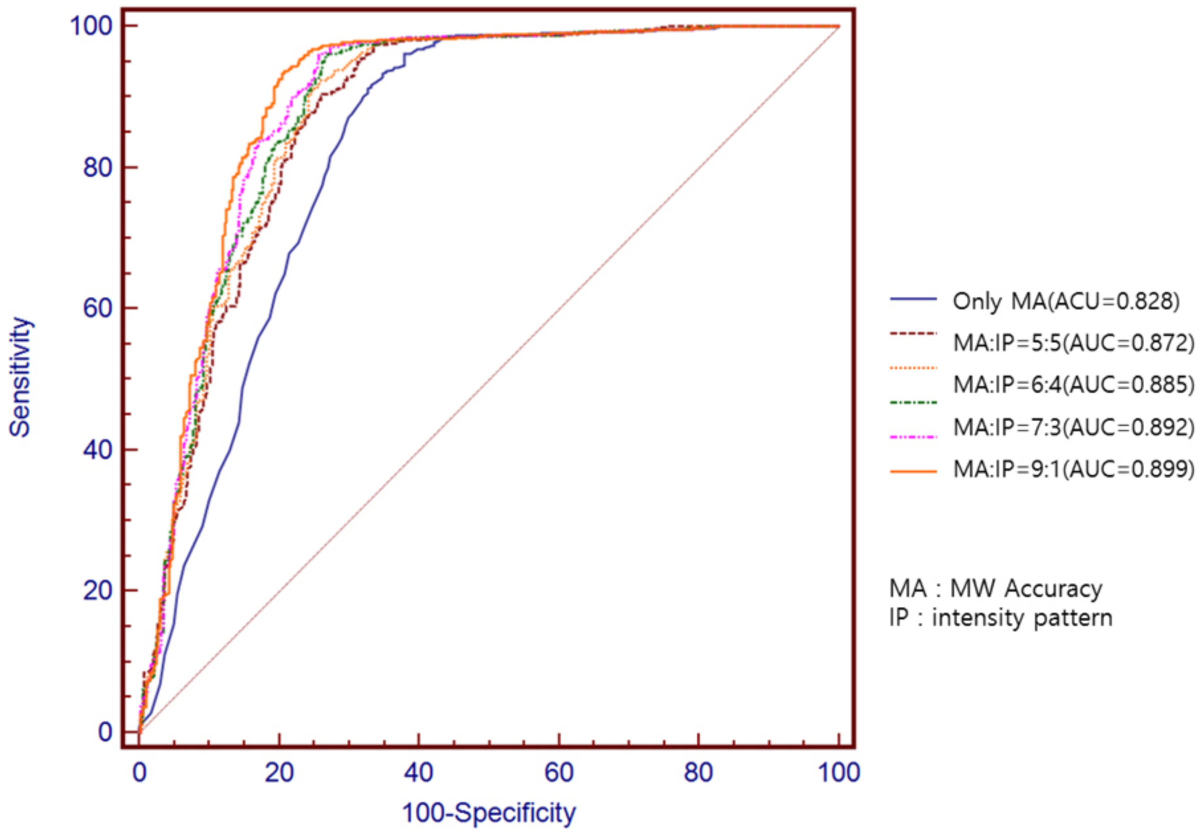

Supplementary Figure 3. Comparison of the Area Under ROC Curves (AUC) for S-score optimization.

The ratio of M.W. Accuracy (MA) and Intensity Pattern (IP) was considered for determining the best AUC value from the S-score by α1-acid glycoprotein (AGP) standard *N*-glycopeptide analysis. A combination of MA and IP gave a better AUC value than could be obtained using only MA. It appears that higher AUC values were associated with higher IP values. The best MP:IP ratio for S-scoring was determined to be 9:1(AUC=0.899).

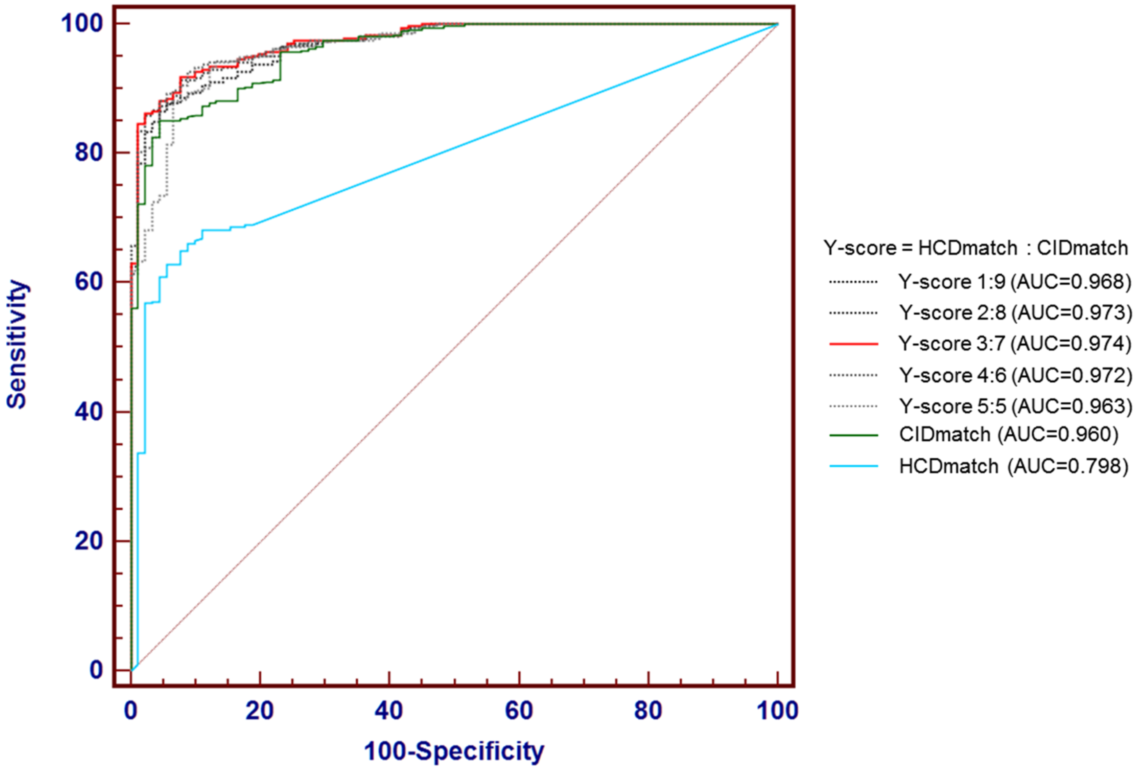

Supplementary Figure 4. Comparison of the Area Under ROC Curves (AUC) for the optimization of Y-scoring.

Weighted values of HCDmatch and CIDmatch for Y-scoring from 0.1 to 0.5 and 0.9 to 0.5, respectively, were considered. The highest AUC value obtained was 0.974, corresponding to weighted values of HCDmatch and CIDmatch of 0.3 and 0.7, respectively. These values were used for Y-score optimization.

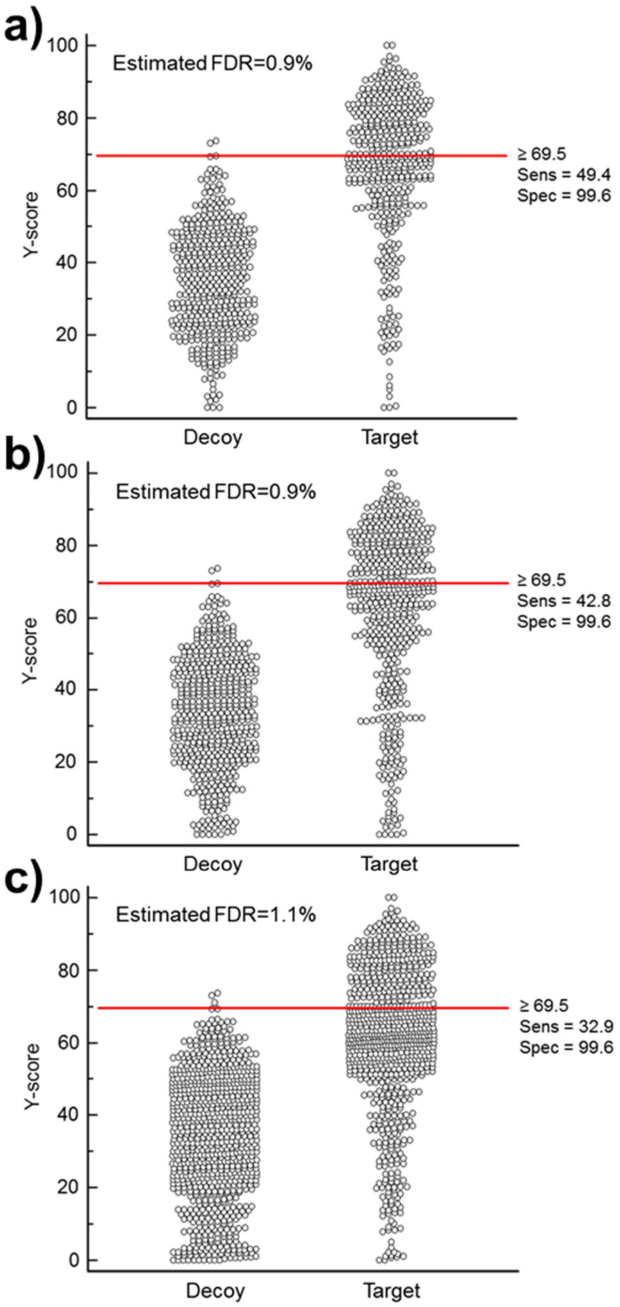

Supplementary Figure 5. Determination of estimated FDR (false-discovery rate) by GPA decoy method using 3 different GPA-databases.

Scatter plots of an analysis of standard α1-acid glycoprotein (AGP) using (a) GPA-DB-AGP: two glycoproteins for AGP1/2, (b) GPA-DB-Mixture: seven glycoprotein mixtures, and (c) GPA-DB-HumanPlasma: 282 glycoproteins from human plasma. The estimated FDR values were similar each other: (a) 0.9%, (b) 0.9%, and (c) 1.1 %.

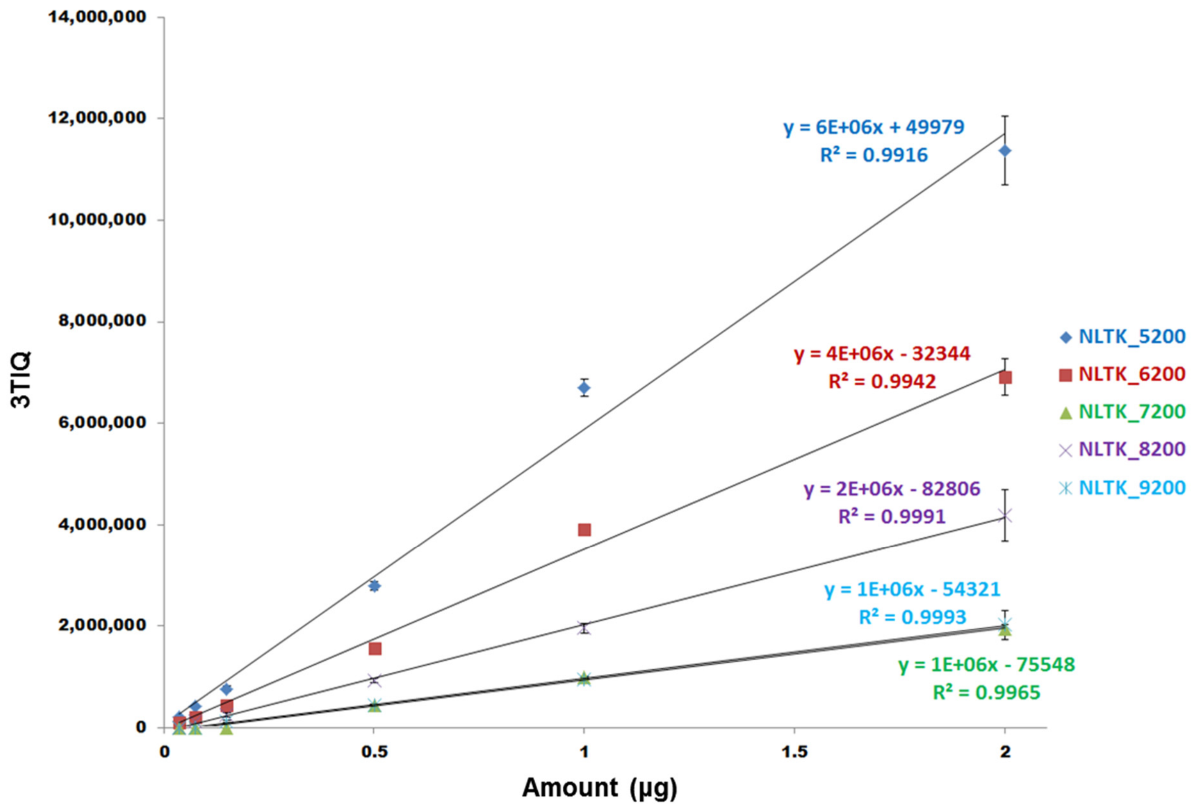

Supplementary Figure 6. Calibration curves of five *N*-glycopeptides from RNase B quantitated by 3TIQ.

Each point represents the average of technical triplicates. For calibration curves of each *N*-glycopeptide, six different amounts of digested RNase B [0.0375 µg (2.5 pmol), 0.075 µg (5 pmol), 0.15 µg (10 pmol), 0.5 µg (33.3 pmol), 1 µg (66.7 pmol), and 2 µg (133.4 pmol)] were prepared and spiked into 0.15 µg (0.35 pmol) of α1-acid glycoprotein (AGP). The calibration curves of all *N*-glycopeptides exhibited good linearity ($R^2 \geq 0.99$).

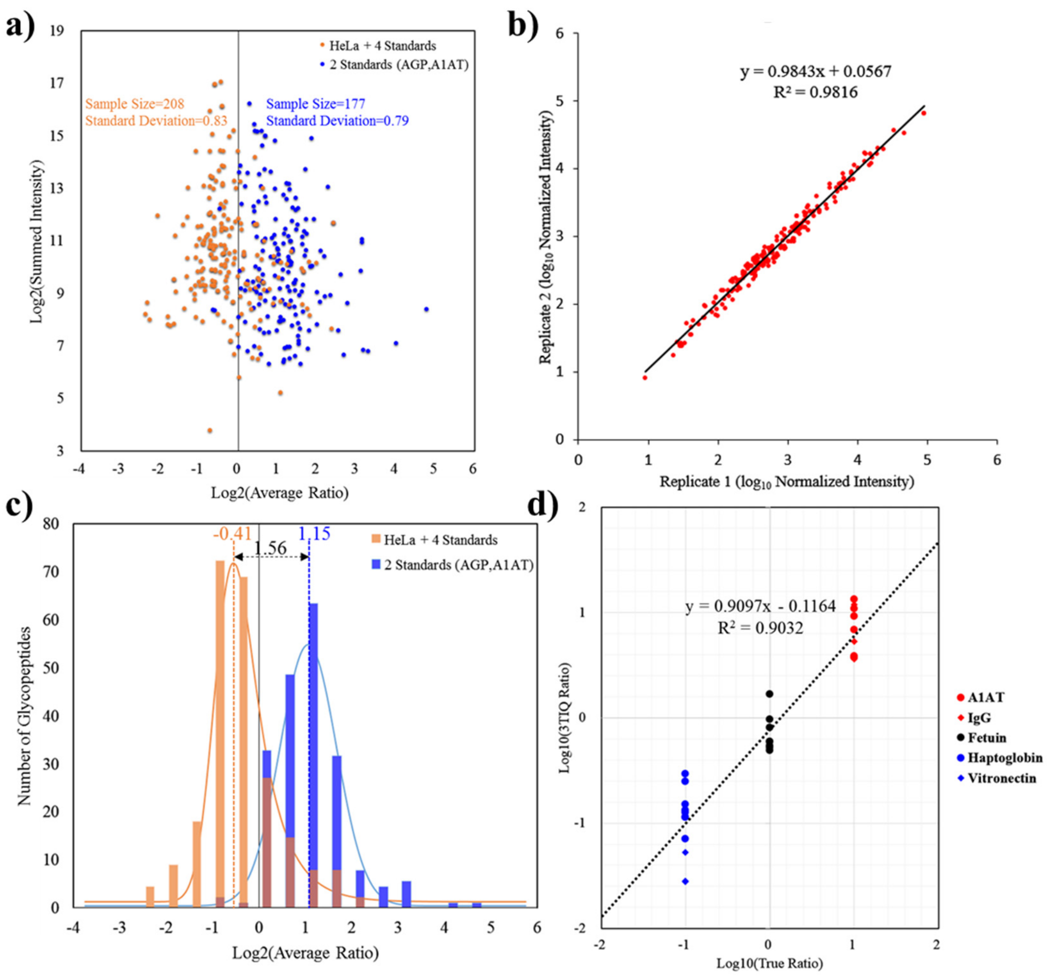

Supplementary Figure 7. Quantification results for the proteome benchmark dataset.

(A) Replicate groups were filtered for two out of three valid values and averaged, and the log ratios of the two standards (AGP and alpha-1-antitrypsin) (blue) / HeLa cell lysates + four standards (IgG, fetuin, vitronectin, and haptoglobin) (orange) 3:1 versus 1:1 samples were plotted against the logarithm of glycopeptide intensities. (B) Comparison of technical replicates in both runs of 1:1 sample. Repeatability of runs indicated by R2 = 0.9816. (C) The histogram of the ratio distributions of HeLa + four standards (IgG, fetuin, vitronectin, and haptoglobin) and two standards (AGP and Alpha-1-antitrypsin) N-glycopeptides obtained using the 3TIQ method. (D) Quantification results of benchmark dataset showing dynamic range. The logarithm of experimental abundance ratio of glycopeptides obtained from 3TIQ method against the logarithm of true abundance ratio between different amount of sample mixtures from fetuin, IgG, A1AT, vitronectin, and haptoglobin.

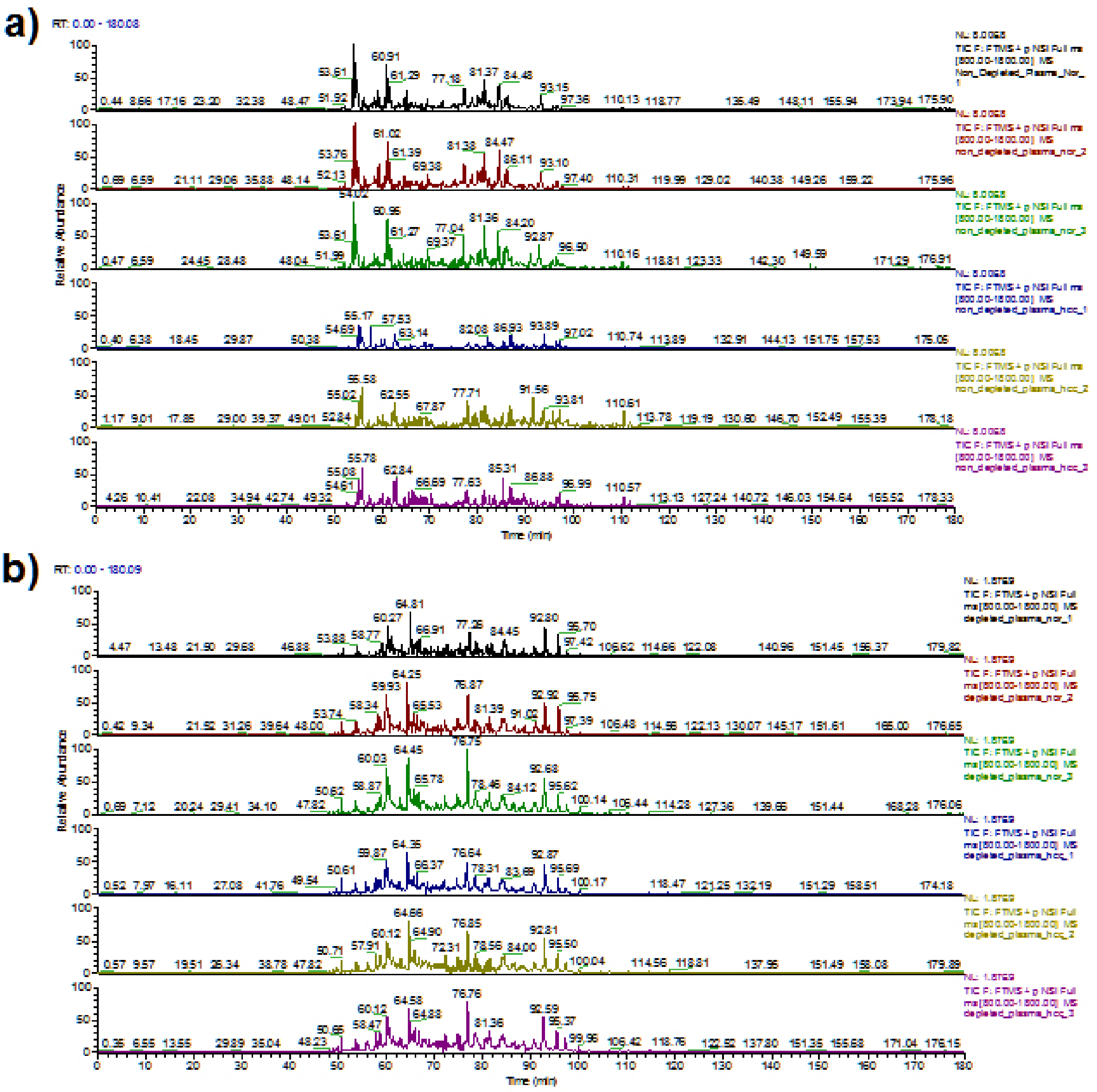

Supplementary Figure 8. Chromatograms from 12 human plasma samples.

(a) TICs of non-depleted normal (upper three) or HCC (lower three) plasma. (b) TICs of depleted normal (upper three) and HCC (lower three) plasma. Each sample was individually enriched by HILIC, and then analyzed by nano-UPLC-LTQ Orbitrap MS.

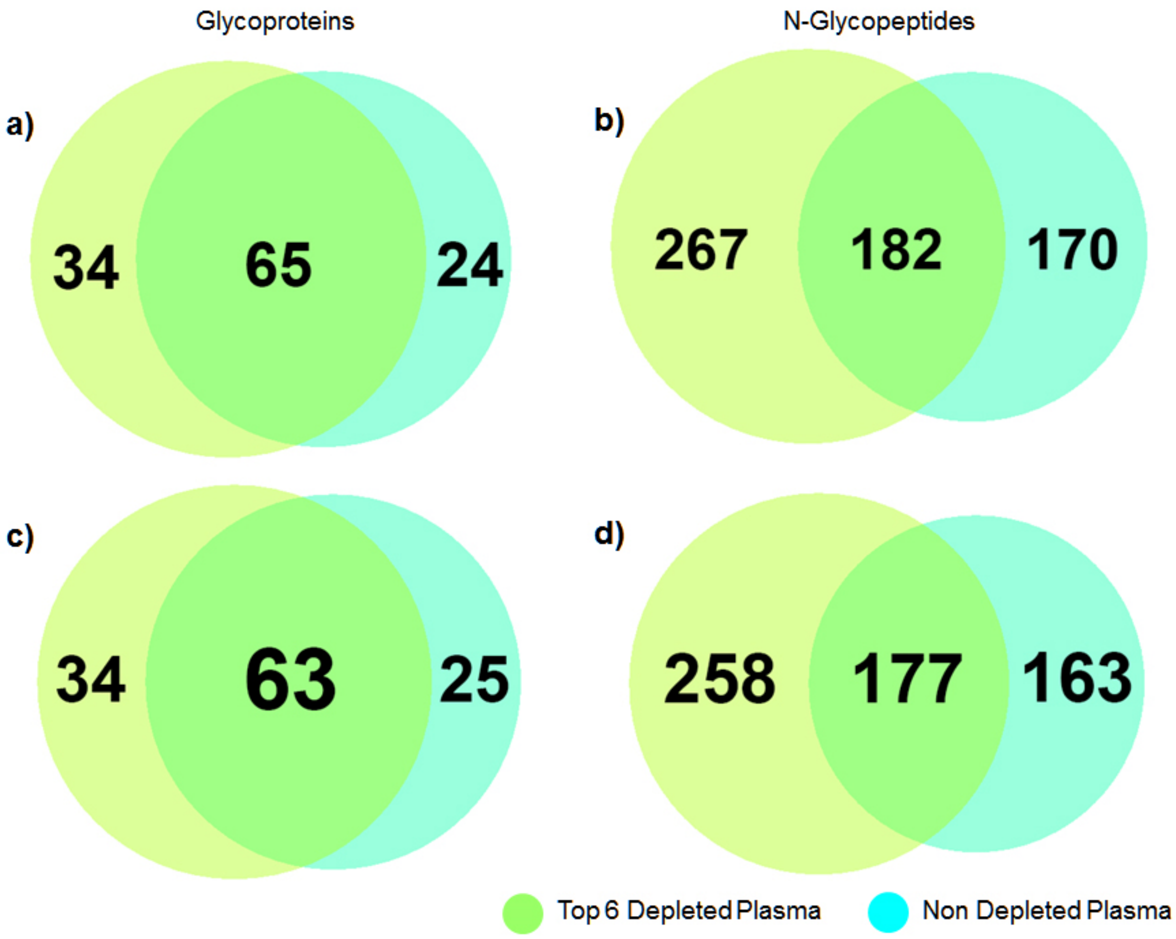

Supplementary Figure 9. Venn diagrams of the number of N-glycoproteins and *N*-glycopeptides identified in 6 protein depleted and non-depleted plasma.

(a) Number of 123 N-glycoproteins identified. (b) Number of 619 *N*-glycopeptides identified. (c) Number of 122 N-glycoproteins quantitated. (d) Number of 598 *N*-glycopeptides quantitated.

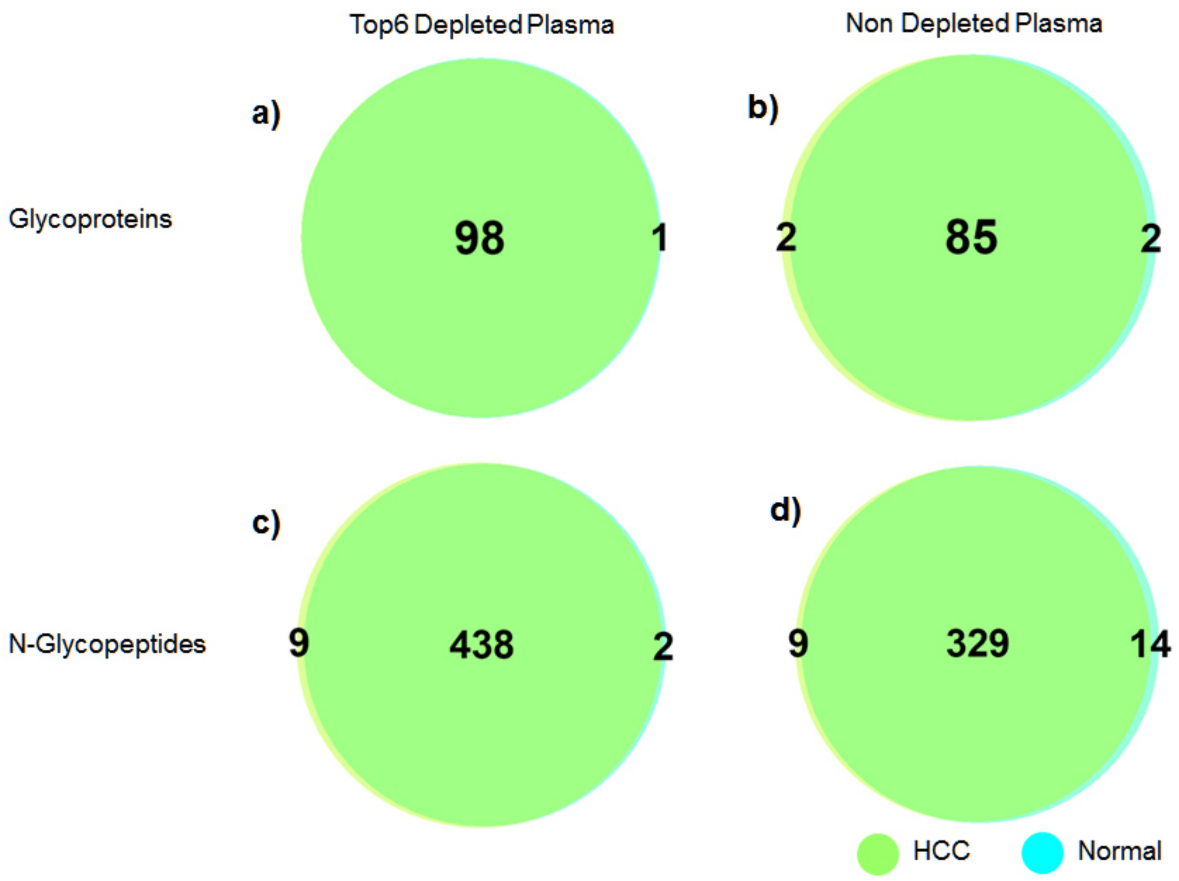

Supplementary Figure 10. Venn diagrams of the number of analyzed N-glycoproteins and *N*-glycopeptides identified in HCC and normal human plasma.

(a) Number of 99 N-glycoproteins identified in depleted plasma. (b) Number of 99 *N*-glycoproteins identified in non-depleted plasma (c) Number of 449 *N*-glycopeptides identified in depleted plasma (d) Number of 352 *N*-glycopeptides identified in non-depleted plasma.

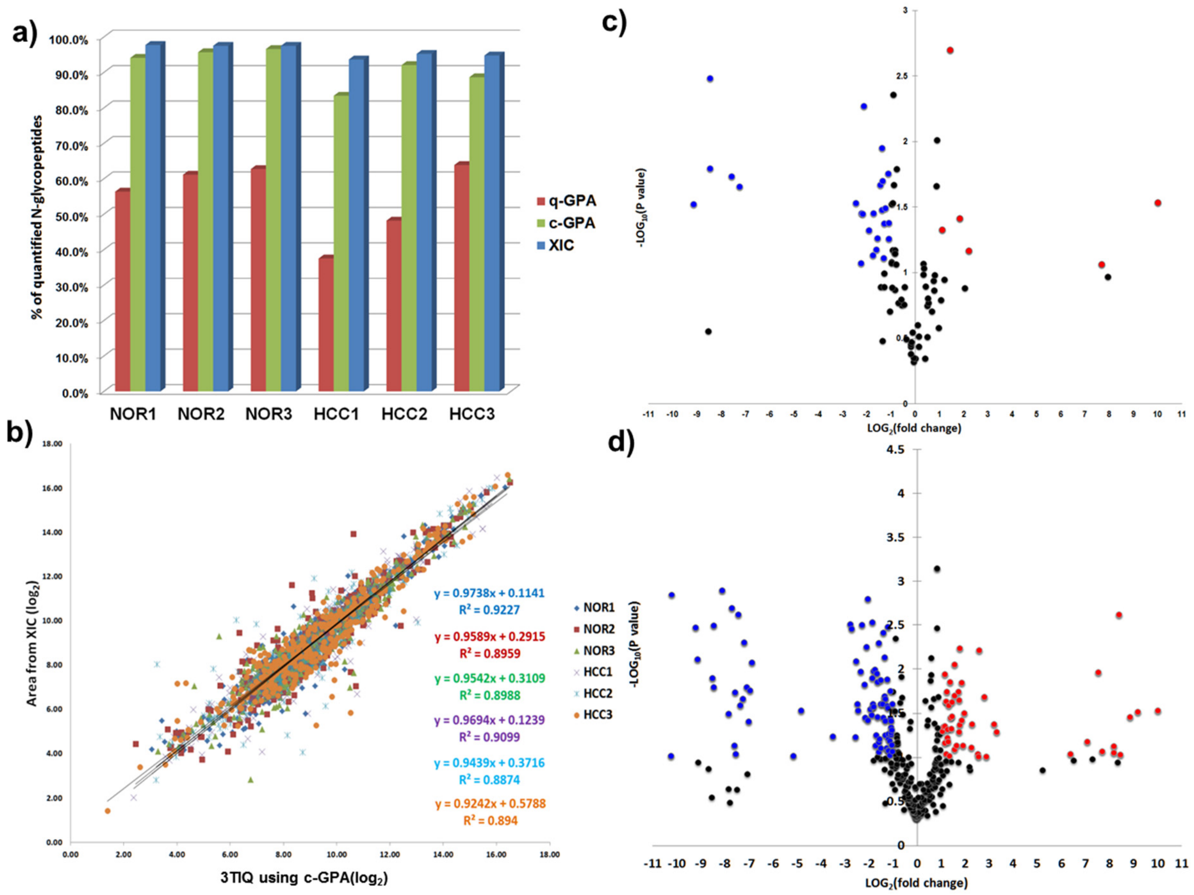

Supplementary Figure 11. Analysis of normal and HCC non-depleted plasma samples by I-GPA.

(a) Results of label-free quantitative analysis, using c-GPA, of *N*-glycopeptides in a non-depleted human plasma sample. (b) Comparison of label-free quantitation based on the three most intense isotope peaks (3TIQ) in non-depleted human plasma, after *N*-glycopeptides with coefficients of variation of <30% were filtered out. (d) and (e) Volcano plot showing log (fold change) versus log(P-value) of differentially expressed N-glycoproteins (d) or *N*-glycopeptides(e) with blue and red circles indicating down-regulation and up-regulation, respectively.

a)

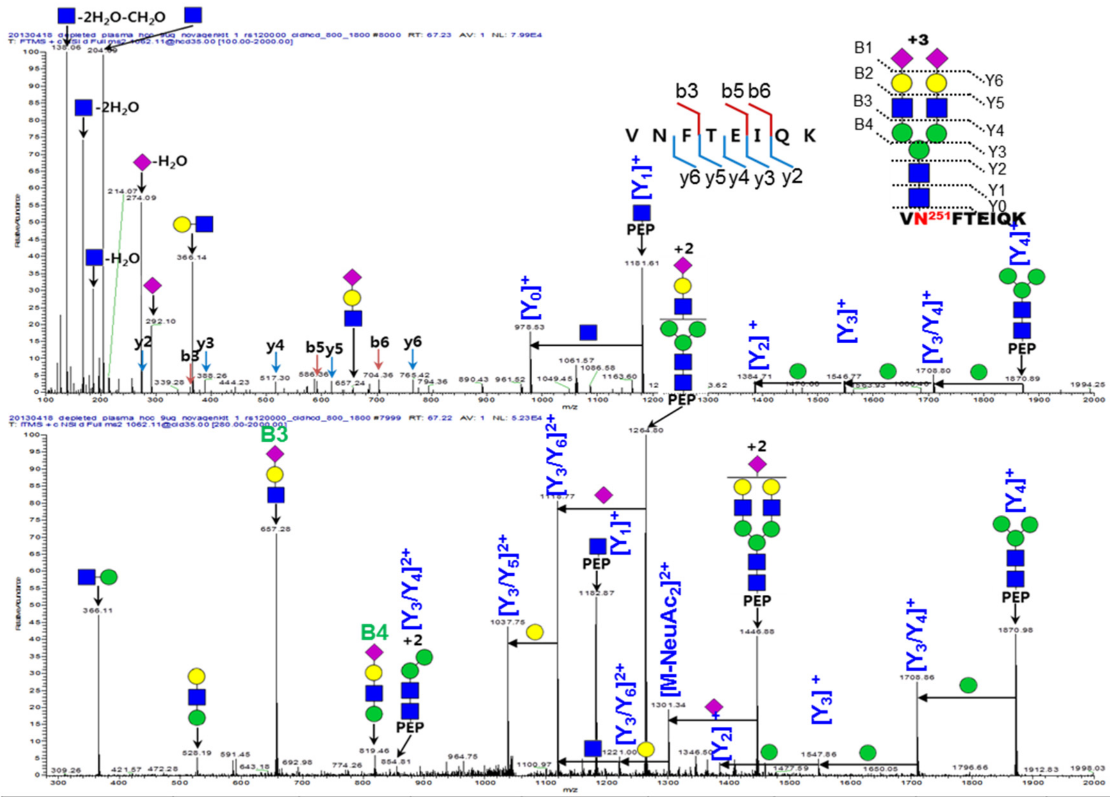

b)

c)

Identification	m/z	charge	RT	M-score	S-score	HCDmatch	CIDmatch	Y-score
	1061.769	3+	67.23	2.31	99.69	71.5%	87.5%	82.7
Quantification	3TIQ by c-GPA						log2(HCC/NOR)	
	Normal1	Normal2	Normal3	HCC1	HCC2	HCC3		
	ND	ND	ND	3,122,505	2,941,621	3,370,720	7.9	

Supplementary Figure 12. Automated identification of site-specific *N*-glycopeptides from α-Fetoprotein (AFP) in HCC human plasma.

VNFTEIQK_5402 from AFP in (a) HCD and (b) CID-MS/MS spectra from HCC human plasma. The HCD spectrum (a) was acquired at high energy, which gives more amide bond breakage, producing b- and y-ions and eight glycan oxonium ions. The CID spectrum (b) gives y-ion *N*-glycopeptide fragment ions, resulting from partial fragmentation of the glycan, and b-ions, glycan fragment ions resulting from the non-reducing ends of the glycan. (c) Detailed information on three scoring results from I-GPA analysis of *N*-glycopeptides from AFP in human plasma.

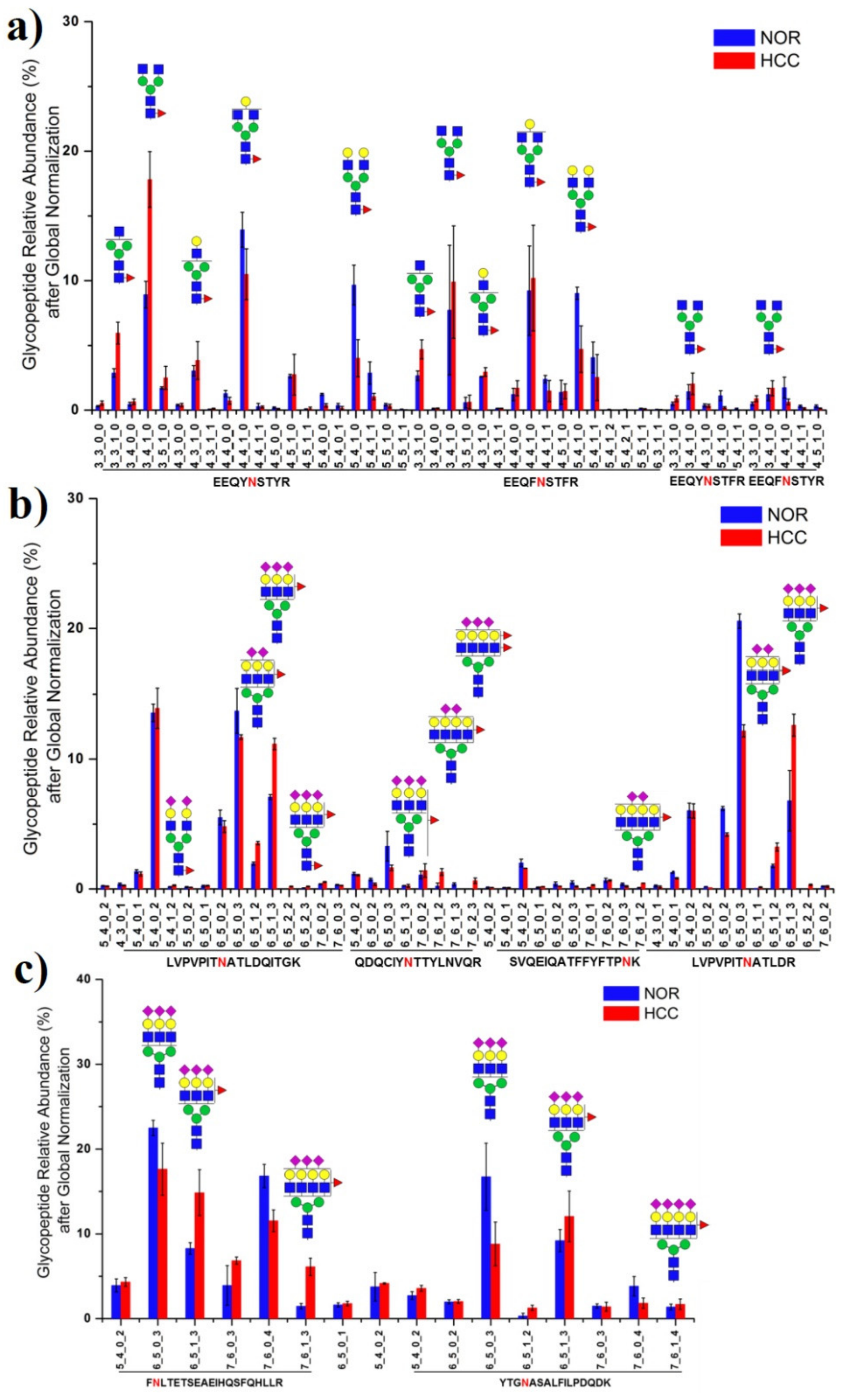

Supplementary Figure 13. Comparison of relative changes in the abundance of site-specific N-glycopeptides in normal and HCC human plasma.

(a) IgG1, 2, and 3/4 isoforms, (b) α1-acid glycoprotein (AGP) 1 and 2 isoforms, and (c) α1-antichymotrypsin (AACT) exhibited independent site-specific changes in *N*-glycopeptide abundance between normal and HCC plasma.

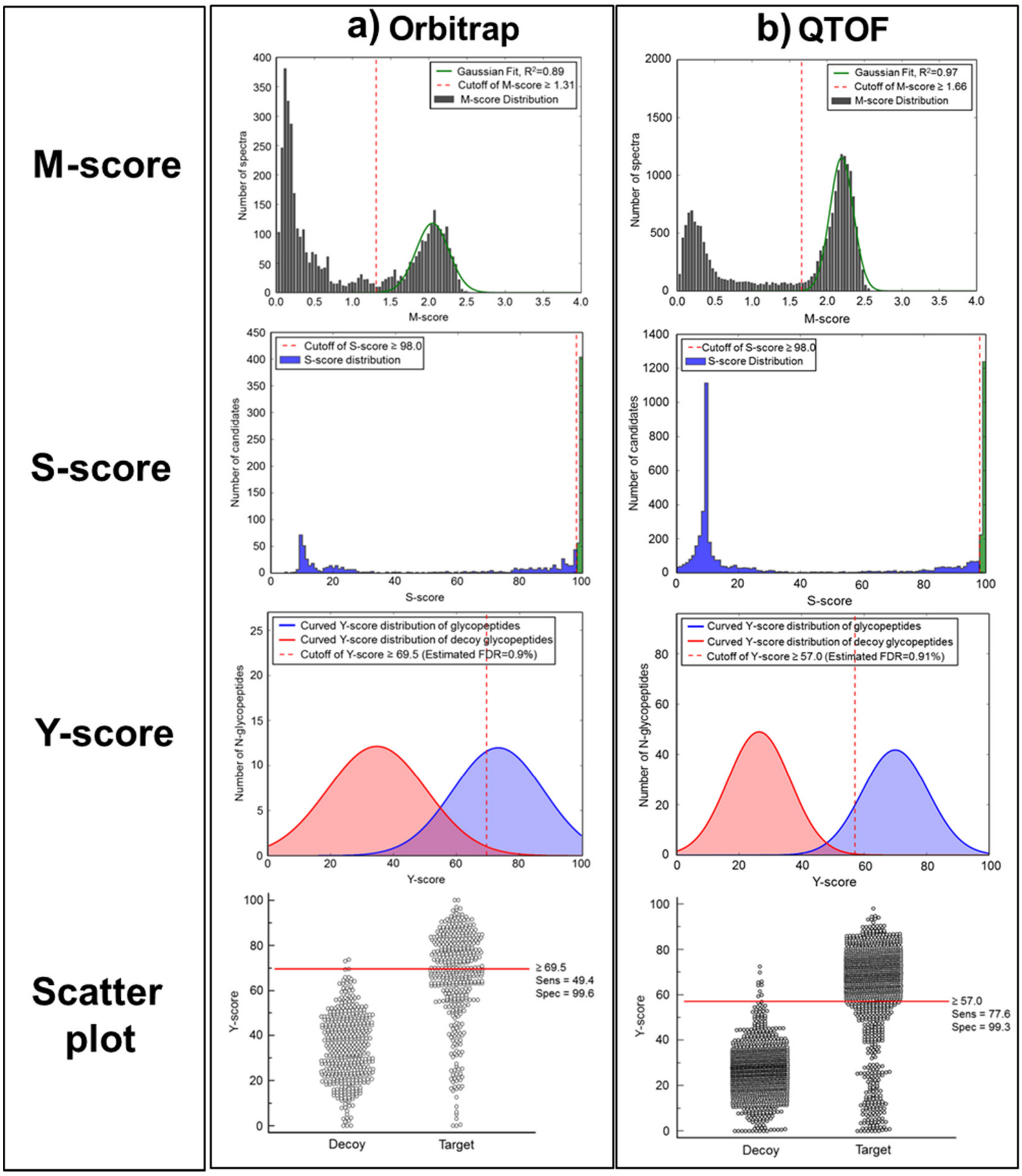

Supplementary Figure 14. Identification of *N*-glycopeptides from standard α1-acid glycoprotein (AGP) using id-GPA search in Orbitrap and QTOF MS analysis.

The id-GPA was designed for identification of site-specific *N*-glycopeptides in high-throughput analysis using three scoring systems (M-, S-, and Y-scores). (a) In Orbitrap MS, the id-GPA gave 456 true positive MS/MS spectra of 95 unique *N*-glycopeptides identified at estimated FDR < 1% using GPA decoy method. (b) In QTOF MS, the id-GPA gave 985 filtered MS/MS spectra of 111 unique *N*-glycopeptides identified at estimated FDR < 1% using GPA decoy method.

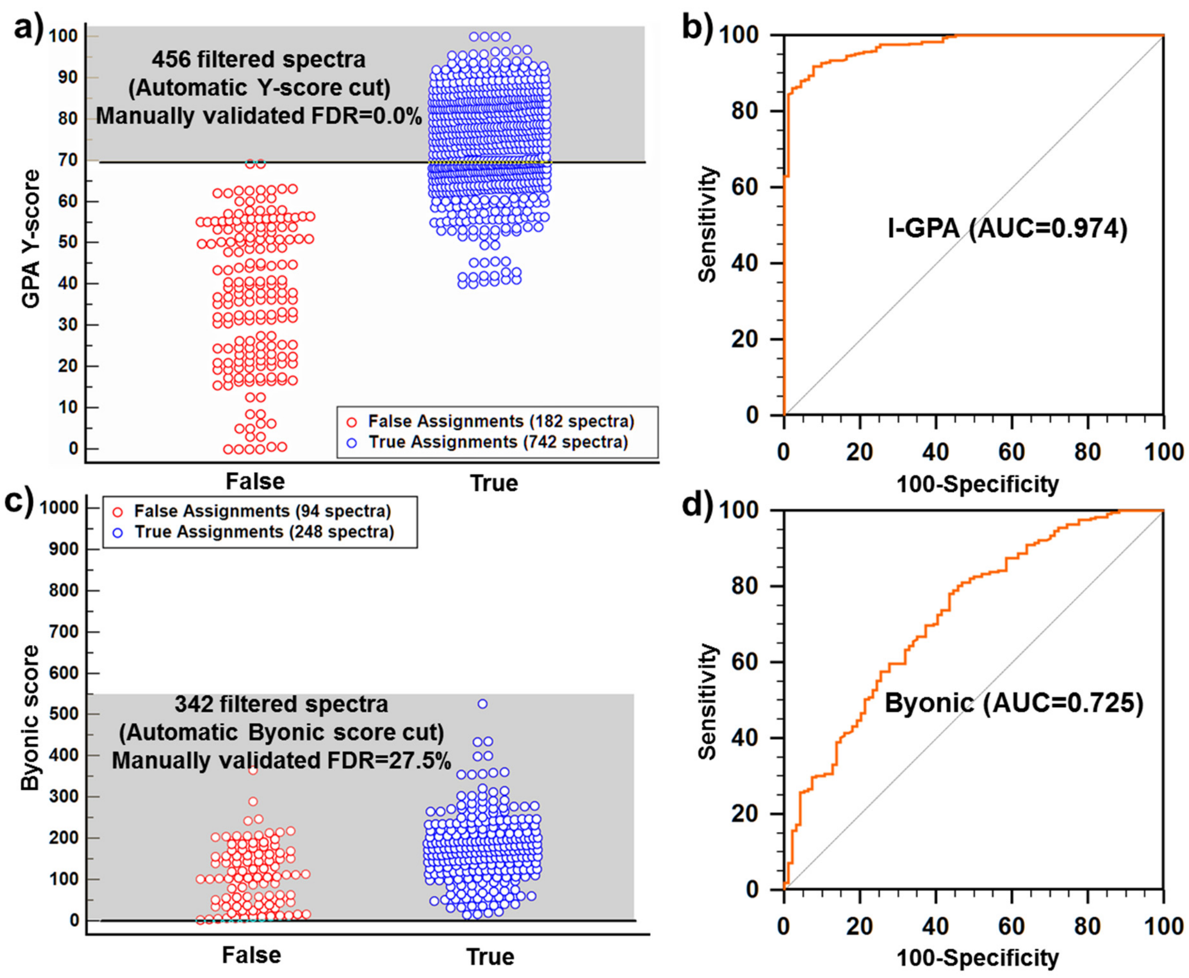

Supplementary Figure 15. The scatter plots (a,c) of manually validated data in the analysis of standard α1-acid glycoprotein (AGP) and their receiver operating characteristic (ROC) curves (b,d) were compared between I-GPA and Byonic tools, respectively.

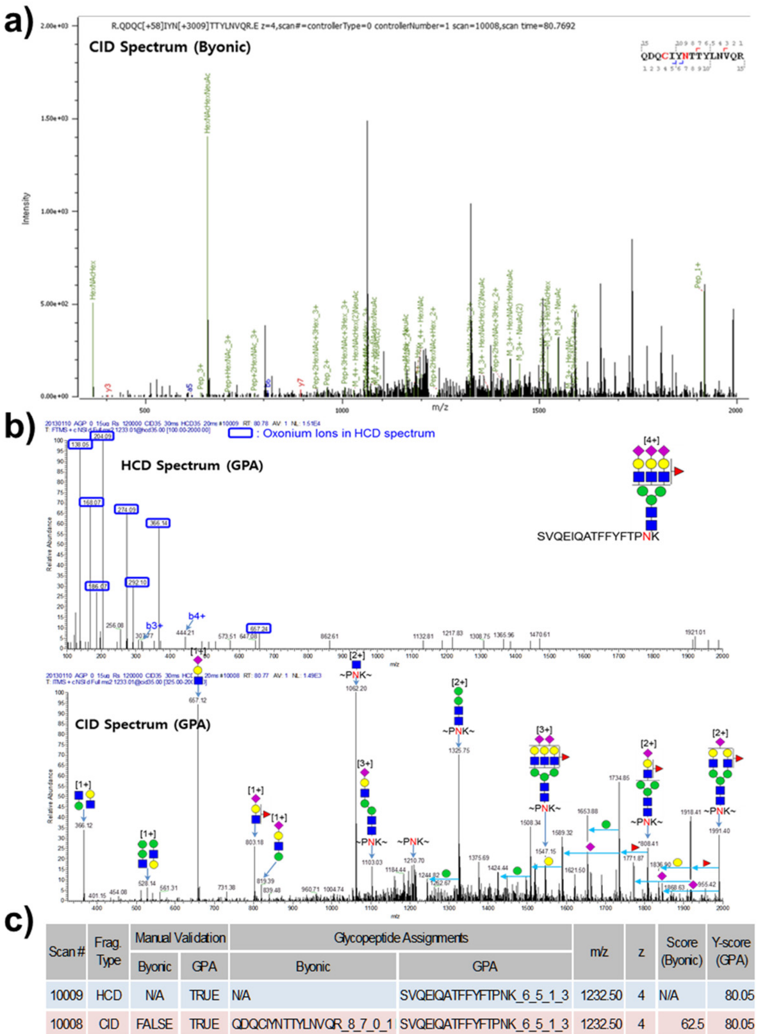

c)

Scan #	Frag. Type	Manual Validation		Glycopeptide Assignments		m/z	z	Score (Byonic)	Y-score (GPA)
		Byonic	GPA	Byonic	GPA				
10009	HCD	N/A	TRUE	N/A	SVQEIQATFFYFTPNK_6_5_1_3	1232.50	4	N/A	80.05
10008	CID	FALSE	TRUE	QDQCIYNTTYLNVQR_8_7_0_1	SVQEIQATFFYFTPNK_6_5_1_3	1232.50	4	62.5	80.05

Supplementary Figure 16. Exemplary spectrum of false positive assignments by Byonic in the analysis of *N*-glycopeptides from standard α1-acid glycoprotein (AGP).

Byonic resulted in incorrect identification of *N*-glycopeptide because of poor assignment of fragment ions in CID MS/MS spectrum. And, its corresponding HCD MS/MS spectrum showed no identification. In CID MS/MS spectrum, the intense fragment ions presenting sequential glycosyl losses were not assigned by Byonic (a), while they were clearly assigned by id-GPA (b-bottom) confirming it as *N*-glycopeptide (SVQ…PNK_6513). And b- and y ions by peptide backbone fragmentation were well matched in its corresponding HCD MS/MS spectrum (b-top). Detailed score informations about *N*-glycopeptide identification by Byonic and id-GPA are shown in table (c) as well as and manual validation results.

a)

b)

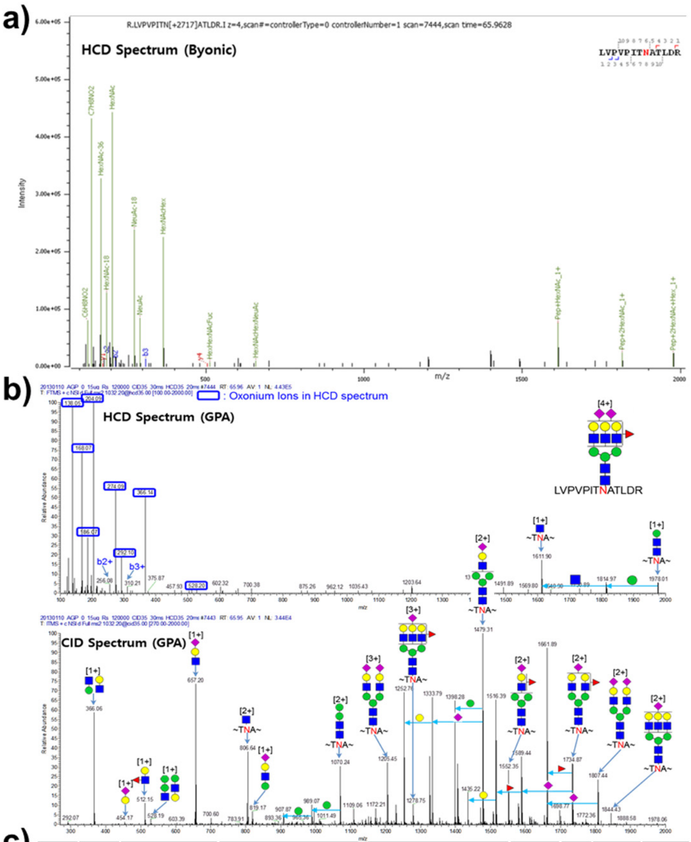

c)

Scan #	Frag. Type	Manual Validation		Glycopeptide Assignments		m/z	z	Score (Byonic)	Y-score (GPA)
		Byonic	GPA	Byonic	GPA				
7444	HCD	FALSE	TRUE	LVPVPITNATLDR_6_5_3_1	LVPVPITNATLDR_6_5_1_2	1031.95	4	218.1	87.8
7443	CID	N/A	TRUE	N/A	LVPVPITNATLDR_6_5_1_2	1031.95	4	N/A	87.8

Supplementary Figure 17. Exemplary spectrum of false positive assignments by Byonic in the analysis of *N*-glycopeptides from standard α1-acid glycoprotein (AGP).

Byonic resulted in incorrect identification of *N*-glycopeptide because of poor assignment of fragment ions in HCD MS/MS spectrum. And, its corresponding CID MS/MS spectrum showed no identification. In HCD MS/MS spectrum, only a few the fragment ions generating by sequential glycosyl losses were assigned by Byonic. It leads a false identification as LVPVPITNATLDR_6531 in glycoform attatched to peptides (a). However, id-GPA resulted in true identification by combined search of HCD and CID MS/MS spectrum (b), where CID MS/MS showed clearly the fragment ions generating by sequential glycosyl losses to correctly identify *N*-glycopeptides as LVPVPITNATLDR_6512 including glycoform. Detailed score informations about *N*-glycopeptide identification by Byonic and id-GPA are shown in table (c) as well as and manual validation results.

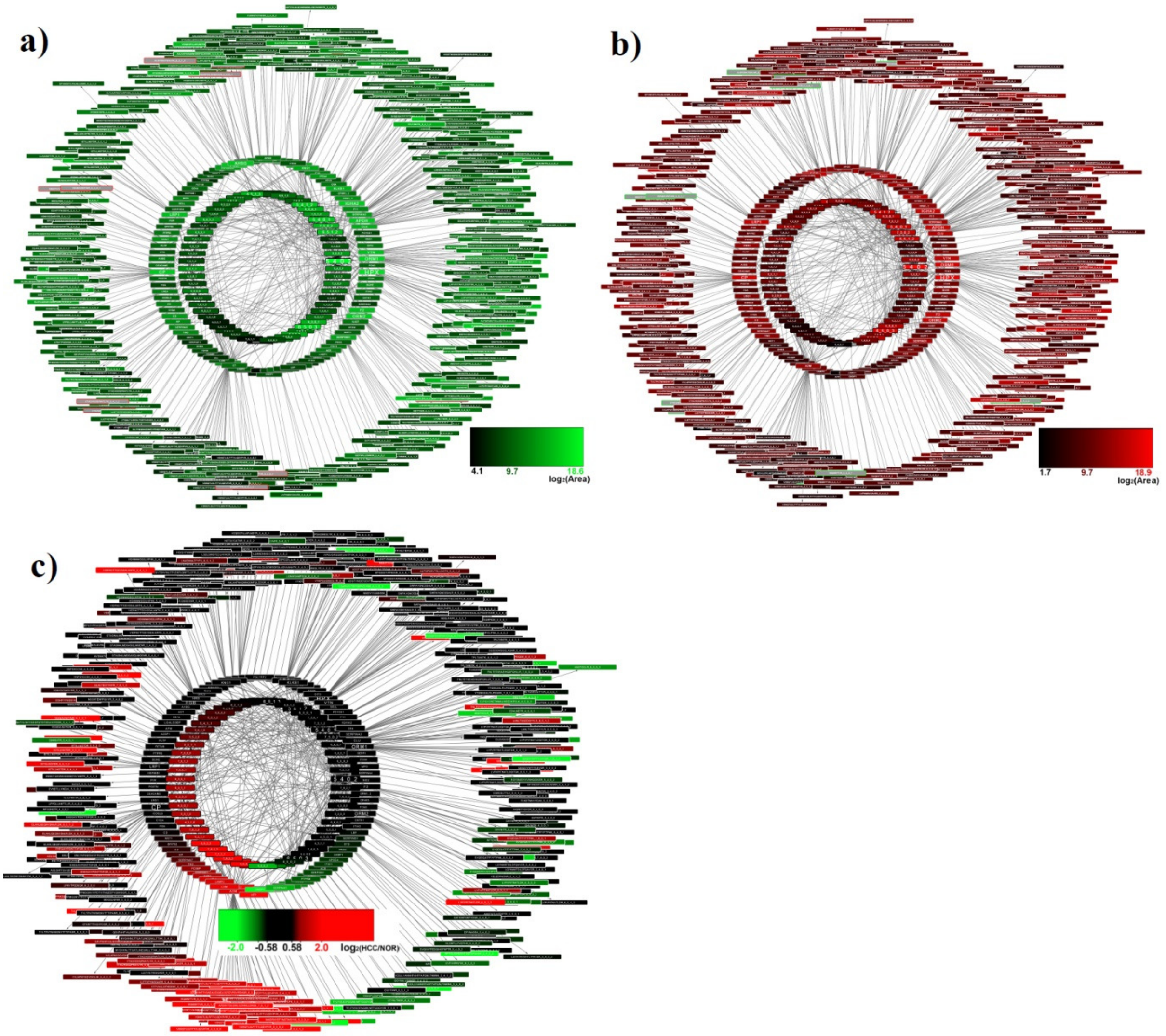

Supplementary Figure 18. Label-free quantitative analysis of depleted normal and HCC plasma samples using I-GPA.

Global mapping of glycans, N-glycoproteins, and site-specific *N*-glycopeptides in depleted human plasma. I-maps represent (a) normal, (b) HCC, and (c) glycans, glycoproteins, and *N*-glycopeptides differently expressed between normal and HCC human plasma; green indicates lower concentrations and red indicates higher concentrations.

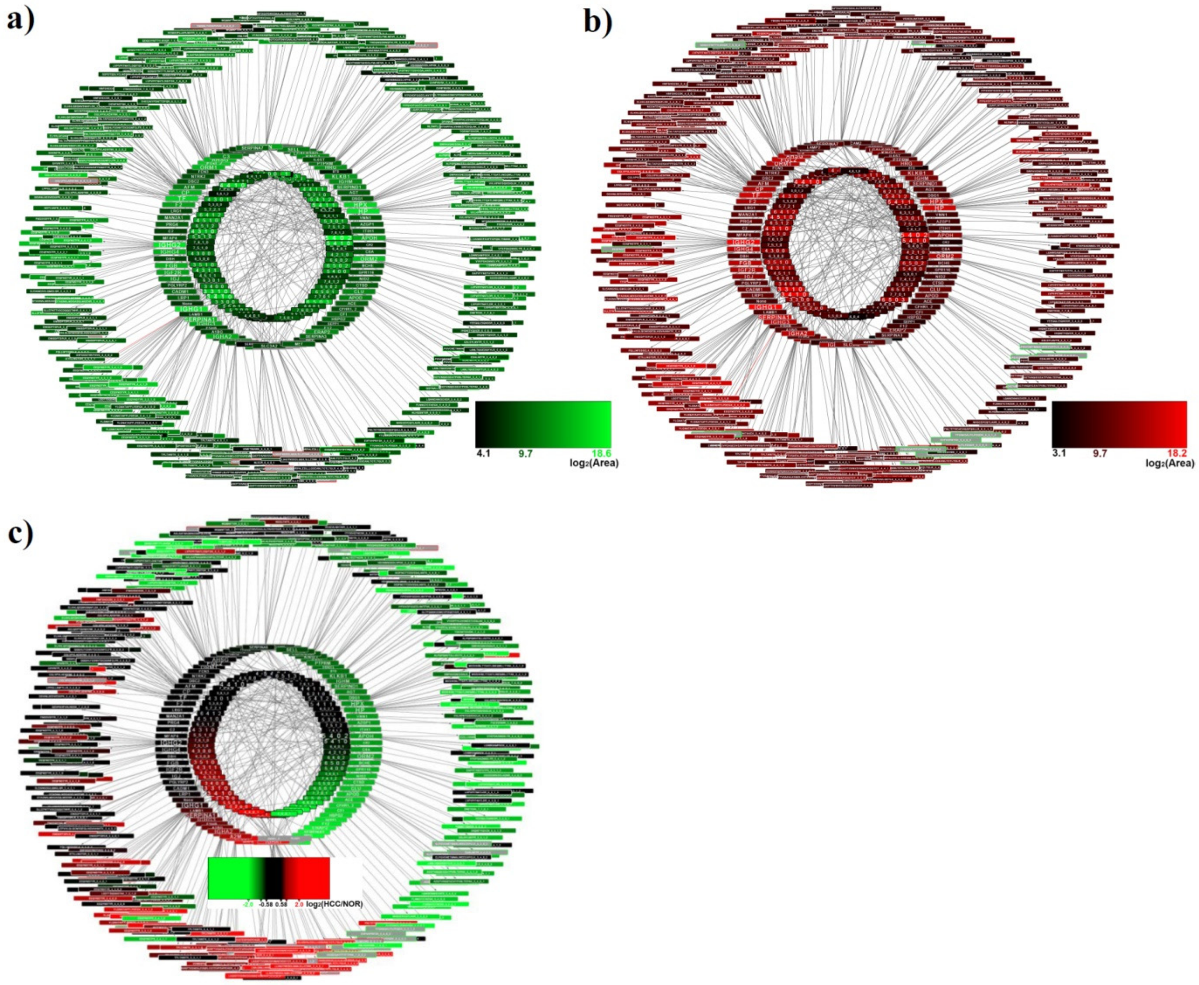

Supplementary Figure 19. Label-free quantitative analysis of non-depleted normal and HCC plasma samples using I-GPA

Global mapping of glycans, *N*-glycoproteins, and site-specific *N*-glycopeptides in non-depleted human plasma. I-maps represent (a) normal, (b) HCC, and (c) glycans, glycoproteins, and *N*-glycopeptides differently expressed between normal and HCC human plasma; green indicates lower concentrations and red indicates higher concentrations.

Supplementary Tables

Supplementary Table 1. List of tryptic peptides used for construction of GPA-DB-AGP and GPA-DB-Mixture.

No.	Glycoproteins	Uniprot accession numbers	*N*-Glycosylation sites	Tryptic Peptide Sequences
1	AGP1	P02763	33	LVPVPITNATLDQITGK*
2	AGP1	P02763	33	CANLVPVPITNATLDQITGK*
3	AGP1	P02763	33	QIPLCANLVPVPITNATLDQITGK
4	AGP2	P19652	33	LVPVPITNATLDR*
5	AGP2	P19652	33	CANLVPVPITNATLDR*
6	AGP2	P19652	33	QIPLCANLVPVPITNATLDR
7	AGP1,2	P02763,P19652	56,56	NEEYNK
8	AGP1,2	P02763,P19652	72,72	SVQEIQATFFYFTPNK
9	AGP1	P02763	93	QDQCIYNTTYLNVQR
10	AGP1	P02763	103	ENGTISR
11	AGP2	P19652	93	QNQCFYNSSYLNVQR
12	AGP2	P19652	103	ENGTVSR
13	IgG1	P01857	180	EEQYNSTYR
14	IgG2	P01859	176	EEQFNSTFR
15	IgG3	P01860	227	EEQYNSTFR
16	IgG3	P01860	322	GFYPSDIAVEWESSGQPENNYNTTPPMLDSDGSFFLYSK
17	IgG4	P01861	177	EEQFNSTYR
18	RNase B	P61823	69	NLTK

Red letter is *N*-glycosylation site, * N-terminal truncation

Supplementary Table 2. Efficiency of HILIC enrichment for the identification of *N*-glycopeptides from the α1-acid glycoprotein (AGP) standard sample. Numbers of selected N-glycopeptide spectra and unique *N*-glycopeptides in the AGP standard sample. For in-depth glycoproteome analysis of the AGP (1.5 mg) standard, glycopeptide enrichment was performed by HILIC of tryptic glycopeptides of the standard AGP glycoprotein. We compared two cases by analyzing data sets from AGP standard samples with or without HILIC enrichment. The selected *N*-glycopeptide spectra by M-score showed an 82.4% (3054) increase in the HILIC-enriched result by id-GPA. Finally, when the HILIC enrichment method was used as the target database, 137 (44.2% increase) unique glycopeptides across 469 *N*-glycopeptide spectra were identified at a Y-score threshold > 64.8 (FDR = 0.64).

Samples Sets		Number of *N*-glycopeptide spectra selected by M-score	Number of *N*-glycopeptide candidates selected by S-score	Number of *N*-glycopeptides filtered by Y-score	Number of unique *N*-glycopeptides	Number of unique glycoproteins	Threshold of Y-score (Estimated FDR)
AGP Standard Sample	without HILIC Enrich	1674 (28.8%)	462 (41.8%)	228	95	2	69.5 (0.87%)
	with HILIC Enrich	3054 (53.5%)	944 (41.8%)	469	137	2	64.8 (0.64%)

Supplementary Table 3. Examples of target and decoy fragment ions (Y/B) generated from tryptic *N*-glycopeptide (ENGTISR_5402) of standard α1-acid glycoprotein (AGP).

Target Y-ions	Decoy Y-ions	Target B-ions	Decoy B-ions
ENGTISR_0_0_0_0	RSITGNE_0_0_0_0	1_1_0_0	1_1_0_0
ENGTISR_0_1_0_0	RSITGNE_1_0_0_0	1_0_0_1	0_1_1_0
ENGTISR_0_2_0_0	RSITGNE_2_0_0_0	2_1_0_0	1_2_0_0
ENGTISR_1_2_0_0	RSITGNE_2_1_0_0	1_1_0_1	1_1_1_0
ENGTISR_2_2_0_0	RSITGNE_2_2_0_0	2_1_0_1	1_2_1_0
ENGTISR_3_2_0_0	RSITGNE_2_3_0_0		
ENGTISR_3_3_0_0	RSITGNE_3_3_0_0		
ENGTISR_3_3_0_1	RSITGNE_3_3_1_0		
ENGTISR_3_4_0_0	RSITGNE_4_3_0_0		
ENGTISR_4_3_0_0	RSITGNE_3_4_0_0		
ENGTISR_4_3_0_1	RSITGNE_3_4_1_0		
ENGTISR_4_4_0_0	RSITGNE_4_4_0_0		
ENGTISR_4_4_0_1	RSITGNE_4_4_1_0		
ENGTISR_5_4_0_0	RSITGNE_4_5_0_0		
ENGTISR_5_4_0_1	RSITGNE_4_5_1_0		

Glycopeptide nomenclature; PEP_#Hex_#HexNac_#Fuc_#NeuAc.

Supplementary Table 4. Evaluation of FDR with GPA decoy method using different sizes of GPA-databases (two glycoproteins for AGP1/2, seven glycoprotein mixtures, and 282 glycoproteins from human plasma) for the analysis of standard α1-acid glycoprotein (AGP). The id-GPA yielded similar numbers of filtered spectra, assigned spectra, and unique *N*-glycopeptides. Regardless of different sizes of GPA-databases, the estimated false discovery rate (FDR) was consistently ranged from 0.9 to 1.1%, while manually validated FDR gave similar results from 0.0 to 1.3%.

	GPA-DB-AGP (4,214 *N*-glycopeptides)	GPA-DB-Mixture (6,318 *N*-glycopeptides)	GPA-DB-HumanPlasma (253,422 *N*-glycopeptides)
Number of filtered spectra (Y-score ≥ 69.5)	456	456	456
Number of AGP assigned spectra	456	456	450
Number of unique *N*-glycopeptides of AGP	95	95	92
Estimated FDR[a]	0.9%	0.9%	1.1%
Manually validated FDR	0.0%	0.0%	1.3%

[a]Estimated FDR values calculated by GPA decoy method

Supplementary Table 5. Comparison of α1-acid glycoprotein (AGP) *N*-glycopeptides identified using GPA-DB-AGP, GPA-DB-Mixture, and GPA-DB-HumanPlasma.

RT	Scan (HCD_CID)	GPA-DB-AGP	S-score	S.Rank	Y-score	GPA-DB-Mixture	S-score	S.Rank	Y-score	GPA-DB-HumanPlasma	S-score	S.Rank	Y-score
48.58	4628_4627	NEEYNK_5_4_0_0	98.93	1	86.7	NEEYNK_5_4_0_0	98.93	1	86.7	NEEYNK_5_4_0_0	98.93	2	86.7
49.14	4692_4691	NEEYNK_5_4_0_1	99.94	1	92.5	NEEYNK_5_4_0_1	99.94	1	92.5	NEEYNK_5_4_0_1	99.94	1	92.5
49.16	4694_4693	NEEYNK_5_4_0_1	99.93	1	89.6	NEEYNK_5_4_0_1	99.93	1	89.6	NEEYNK_5_4_0_1	99.93	1	89.6
49.21	4700_4699	NEEYNK_6_5_0_1	99.28	1	89.1	NEEYNK_6_5_0_1	99.28	1	89.1	NEEYNK_6_5_0_1	99.28	4	89.1
49.27	4707_4706	ENGTISR_7_6_0_1	99.86	1	86.9	ENGTISR_7_6_0_1	99.86	1	86.9	ENGTISR_7_6_0_1	99.86	1	86.9
49.29	4709_4708	NEEYNK_6_5_1_1	99.10	1	84.4	NEEYNK_6_5_1_1	99.10	1	84.4	NEEYNK_6_5_1_1	99.10	2	84.4
49.38	4718_4717	NEEYNK_4_3_0_1	99.80	1	71.5	NEEYNK_4_3_0_1	99.80	1	71.5	NEEYNK_4_3_0_1	99.80	1	71.5
49.45	4725_4724	ENGTISR_6_5_0_1	99.84	1	100.0	ENGTISR_6_5_0_1	99.84	1	100.0	ENGTISR_6_5_0_1	99.84	2	100.0
49.48	4729_4728	ENGTISR_6_5_1_1	99.94	1	88.3	ENGTISR_6_5_1_1	99.94	1	88.3	ENGTISR_6_5_1_1	99.94	2	88.3
49.52	4733_4732	ENGTISR_5_4_0_1	99.97	1	91.4	ENGTISR_5_4_0_1	99.97	1	91.4	ENGTISR_5_4_0_1	99.97	1	91.4
49.76	4762_4761	NEEYNK_6_5_0_2	99.94	1	95.8	NEEYNK_6_5_0_2	99.94	1	95.8	NEEYNK_6_5_0_2	99.94	2	95.8
49.82	4769_4768	NEEYNK_5_4_0_2	99.93	1	91.4	NEEYNK_5_4_0_2	99.93	1	91.4	NEEYNK_5_4_0_2	99.93	1	91.4
49.84	4771_4770	ENGTISR_7_6_0_2	99.93	1	95.5	ENGTISR_7_6_0_2	99.93	1	95.5	ENGTISR_7_6_0_2	99.93	1	95.5
49.95	4786_4785	NEEYNK_5_4_0_2	99.89	1	96.4	NEEYNK_5_4_0_2	99.89	1	96.4	NEEYNK_5_4_0_2	99.89	2	96.4
49.96	4788_4787	ENGTISR_7_6_1_2	99.68	1	90.5	ENGTISR_7_6_1_2	99.68	1	90.5	ENGTISR_7_6_1_2	99.68	1	90.5
50.02	4795_4794	ENGTISR_6_5_1_2	99.82	1	86.7	ENGTISR_6_5_1_2	99.82	1	86.7	ENGTISR_6_5_1_2	99.82	1	86.7
50.22	4821_4820	ENGTISR_5_4_0_1	99.97	1	78.0	ENGTISR_5_4_0_1	99.97	1	78.0	ENGTISR_5_4_0_1	99.97	1	78.0

50.31	4832_4831	ENGTISR_5_4_0_2	99.97	1	84.7	ENGTISR_5_4_0_2	99.97	1	84.7	ENGTISR_5_4_0_2	99.97	1	84.7
50.43	4848_4847	NEEYNK_6_5_0_3	99.76	1	87.7	NEEYNK_6_5_0_3	99.76	1	87.7	NEEYNK_6_5_0_3	99.76	2	87.7
50.62	4872_4871	ENGTISR_7_6_0_3	99.96	1	82.5	ENGTISR_7_6_0_3	99.96	1	82.5	ENGTISR_7_6_0_3	99.96	1	82.5
51.17	4953_4952	ENGTISR_6_5_0_3	98.89	1	87.2	ENGTISR_6_5_0_3	98.89	1	87.2	ENGTISR_6_5_0_3	98.89	1	87.2
61.73	6674_6673	QDQCIYNTTYLNVQR_7_6_0_0	99.95	1	70.0	QDQCIYNTTYLNVQR_7_6_0_0	99.95	1	70.0	QDQCIYNTTYLNVQR_7_6_0_0	99.95	2	70.0
62.93	6889_6888	LVPVPITNATLDR_7_6_0_0	99.94	1	91.3	LVPVPITNATLDR_7_6_0_0	99.94	1	91.3	LVPVPITNATLDR_7_6_0_0	99.94	2	91.3
62.98	6898_6897	QDQCIYNTTYLNVQR_7_6_0_1	99.93	1	88.2	QDQCIYNTTYLNVQR_7_6_0_1	99.93	1	88.2	QDQCIYNTTYLNVQR_7_6_0_1	99.93	3	88.2
62.99	6900_6899	QDQCIYNTTYLNVQR_7_6_0_1	99.56	1	100.0	QDQCIYNTTYLNVQR_7_6_0_1	99.56	1	100.0	QDQCIYNTTYLNVQR_7_6_0_1	99.56	7	100.0
63.01	6903_6902	LVPVPITNATLDR_6_5_1_0	99.86	1	84.3	LVPVPITNATLDR_6_5_1_0	99.86	1	84.3	LVPVPITNATLDR_6_5_1_0	99.86	2	84.3
63.07	6914_6913	LVPVPITNATLDR_6_5_0_0	99.94	1	92.3	LVPVPITNATLDR_6_5_0_0	99.94	1	92.3	LVPVPITNATLDR_6_5_0_0	99.94	1	92.3
63.28	6953_6952	QDQCIYNTTYLNVQR_6_5_0_1	99.60	1	70.0	QDQCIYNTTYLNVQR_6_5_0_1	99.60	1	70.0	ALGFENATQALGR_7_7_0_1	99.56	12	83.7
63.35	6966_6965	LVPVPITNATLDR_5_4_0_0	99.80	1	81.0	LVPVPITNATLDR_5_4_0_0	99.80	1	81.0	LVPVPITNATLDR_5_4_0_0	99.80	1	81.0
63.38	6973_6972	LVPVPITNATLDR_5_4_0_0	99.72	1	78.1	LVPVPITNATLDR_5_4_0_0	99.72	1	78.1	LVPVPITNATLDR_5_4_0_0	99.72	1	78.1
63.81	7050_7049	LVPVPITNATLDR_3_3_0_0	99.98	1	74.4	LVPVPITNATLDR_3_3_0_0	99.98	1	74.4	LVPVPITNATLDR_3_3_0_0	99.98	1	74.4
64.31	7142_7141	QDQCIYNTTYLNVQR_7_6_0_2	99.54	1	74.0	QDQCIYNTTYLNVQR_7_6_0_2	99.54	1	74.0	QDQCIYNTTYLNVQR_7_6_0_2	99.54	3	74.0
64.35	7149_7148	QDQCIYNTTYLNVQR_7_6_0_2	99.70	1	73.2	QDQCIYNTTYLNVQR_7_6_0_2	99.70	1	73.2	QDQCIYNTTYLNVQR_7_6_0_2	99.70	4	73.2
64.47	7171_7170	QDQCIYNTTYLNVQR_6_5_1_2	99.45	1	78.2	QDQCIYNTTYLNVQR_6_5_1_2	99.45	1	78.2	QDQCIYNTTYLNVQR_6_5_1_2	99.45	11	78.2
64.49	7175_7174	QDQCIYNTTYLNVQR_6_5_0_2	98.68	1	82.9	QDQCIYNTTYLNVQR_6_5_0_2	98.68	1	82.9	QDQCIYNTTYLNVQR_6_5_0_2	98.68	1	82.9
64.52	7182_7181	QDQCIYNTTYLNVQR_6_5_0_2	99.70	1	80.9	QDQCIYNTTYLNVQR_6_5_0_2	99.70	1	80.9	QDQCIYNTTYLNVQR_6_5_0_2	99.70	7	80.9
64.57	7191_7190	LVPVPITNATLDR_6_5_1_1	99.94	1	86.2	LVPVPITNATLDR_6_5_1_1	99.94	1	86.2	LVPVPITNATLDR_6_5_1_1	99.94	1	86.2

64.65	7206_7205	LVPVPITNATLDR_6_5_1_1	99.74	1	84.5	LVPVPITNATLDR_6_5_1_1	99.74	1	84.5	LVPVPITNATLDR_6_5_1_1	99.74	1	84.5
64.68	7211_7210	LVPVPITNATLDR_6_5_0_1	99.96	1	82.8	LVPVPITNATLDR_6_5_0_1	99.96	1	82.8	LVPVPITNATLDR_6_5_0_1	99.96	1	82.8
64.71	7217_7216	LVPVPITNATLDR_6_5_0_1	99.30	1	85.5	LVPVPITNATLDR_6_5_0_1	99.30	1	85.5	LVPVPITNATLDR_6_5_0_1	99.30	1	85.5
64.77	7228_7227	LVPVPITNATLDR_3_3_0_0	99.90	1	72.6	LVPVPITNATLDR_3_3_0_0	99.90	1	72.6	LVPVPITNATLDR_3_3_0_0	99.90	1	72.6
64.78	7230_7229	LVPVPITNATLDR_5_4_0_0	99.96	1	79.2	LVPVPITNATLDR_5_4_0_0	99.96	1	79.2	LVPVPITNATLDR_5_4_0_0	99.96	1	79.2
64.80	7233_7232	LVPVPITNATLDR_5_4_0_1	99.95	1	83.8	LVPVPITNATLDR_5_4_0_1	99.95	1	83.8	LVPVPITNATLDR_5_4_0_1	99.95	1	83.8
64.83	7239_7238	LVPVPITNATLDR_6_5_0_0	99.81	1	88.8	LVPVPITNATLDR_6_5_0_0	99.81	1	88.8	LVPVPITNATLDR_6_5_0_0	99.81	1	88.8
64.84	7241_7240	LVPVPITNATLDR_4_3_0_0	99.80	1	75.5	LVPVPITNATLDR_4_3_0_0	99.80	1	75.5	LVPVPITNATLDR_4_3_0_0	99.80	1	75.5
64.89	7250_7249	LVPVPITNATLDR_5_4_0_1	99.94	1	84.6	LVPVPITNATLDR_5_4_0_1	99.94	1	84.6	LVPVPITNATLDR_5_4_0_1	99.94	1	84.6
64.93	7257_7256	LVPVPITNATLDR_5_5_0_1	99.87	1	80.5	LVPVPITNATLDR_5_5_0_1	99.87	1	80.5	LVPVPITNATLDR_5_5_0_1	99.87	1	80.5
65.04	7277_7276	LVPVPITNATLDR_6_5_0_1	99.90	1	86.6	LVPVPITNATLDR_6_5_0_1	99.90	1	86.6	LVPVPITNATLDR_6_5_0_1	99.90	1	86.6
65.05	7279_7278	LVPVPITNATLDR_6_5_0_1	99.83	1	80.7	LVPVPITNATLDR_6_5_0_1	99.83	1	80.7	LVPVPITNATLDR_6_5_0_1	99.83	1	80.7
65.08	7285_7284	LVPVPITNATLDR_5_4_0_1	99.92	1	90.6	LVPVPITNATLDR_5_4_0_1	99.92	1	90.6	LVPVPITNATLDR_5_4_0_1	99.92	1	90.6
65.10	7288_7287	LVPVPITNATLDR_3_3_0_0	99.89	1	71.8	LVPVPITNATLDR_3_3_0_0	99.89	1	71.8	LVPVPITNATLDR_3_3_0_0	99.89	1	71.8
65.12	7292_7291	LVPVPITNATLDR_4_3_0_1	99.98	1	84.9	LVPVPITNATLDR_4_3_0_1	99.98	1	84.9	LVPVPITNATLDR_4_3_0_1	99.98	1	84.9
65.13	7294_7293	LVPVPITNATLDR_5_4_0_0	99.96	1	72.7	LVPVPITNATLDR_5_4_0_0	99.96	1	72.7	LVPVPITNATLDR_5_4_0_0	99.96	1	72.7
65.23	7310_7309	LVPVPITNATLDR_5_4_0_1	99.99	1	88.1	LVPVPITNATLDR_5_4_0_1	99.99	1	88.1	LVPVPITNATLDR_5_4_0_1	99.99	1	88.1
65.32	7327_7326	LVPVPITNATLDR_4_4_0_1	99.77	1	81.1	LVPVPITNATLDR_4_4_0_1	99.77	1	81.1	LVPVPITNATLDR_4_4_0_1	99.77	1	81.1
65.51	7362_7361	LVPVPITNATLDR_7_6_0_2	99.66	1	84.3	LVPVPITNATLDR_7_6_0_2	99.66	1	84.3	LVPVPITNATLDR_7_6_0_2	99.66	2	84.3
65.68	7393_7392	QDQCIYNTTYLNVQR_6_5_0_3	99.88	1	79.3	QDQCIYNTTYLNVQR_6_5_0_3	99.88	1	79.3	QDQCIYNTTYLNVQR_6_5_0_3	99.88	1	79.3

65.72	7400_7399	LVPVPITNATLDR_7_6_0_2	99.56	1	81.5	LVPVPITNATLDR_7_6_0_2	99.56	1	81.5	LVPVPITNATLDR_7_6_0_2	99.56	2	81.5
65.78	7411_7410	LVPVPITNATLDR_7_6_1_2	99.93	1	83.7	LVPVPITNATLDR_7_6_1_2	99.93	1	83.7	LVPVPITNATLDR_7_6_1_2	99.93	2	83.7
65.83	7420_7419	LVPVPITNATLDR_7_6_0_2	99.88	1	86.3	LVPVPITNATLDR_7_6_0_2	99.88	1	86.3	LVPVPITNATLDR_7_6_0_2	99.88	2	86.3
65.96	7444_7443	LVPVPITNATLDR_6_5_1_2	99.89	1	87.8	LVPVPITNATLDR_6_5_1_2	99.89	1	87.8	LVPVPITNATLDR_6_5_1_2	99.89	1	87.8
66.03	7457_7456	LVPVPITNATLDR_6_5_0_2	99.91	1	82.6	LVPVPITNATLDR_6_5_0_2	99.91	1	82.6	LVPVPITNATLDR_6_5_0_2	99.91	1	82.6
66.05	7461_7460	LVPVPITNATLDR_6_5_0_1	99.88	1	93.6	LVPVPITNATLDR_6_5_0_1	99.88	1	93.6	LVPVPITNATLDR_6_5_0_1	99.88	1	93.6
66.13	7475_7474	LVPVPITNATLDR_5_4_0_1	99.91	1	96.9	LVPVPITNATLDR_5_4_0_1	99.91	1	96.9	LVPVPITNATLDR_5_4_0_1	99.91	1	96.9
66.14	7477_7476	LVPVPITNATLDR_3_3_0_0	99.97	1	75.2	LVPVPITNATLDR_3_3_0_0	99.97	1	75.2	LVPVPITNATLDR_3_3_0_0	99.97	1	75.2
66.15	7479_7478	LVPVPITNATLDR_5_4_0_1	99.86	1	85.2	LVPVPITNATLDR_5_4_0_1	99.86	1	85.2	LVPVPITNATLDR_5_4_0_1	99.86	1	85.2
66.19	7486_7485	LVPVPITNATLDR_6_5_0_2	99.96	1	84.1	LVPVPITNATLDR_6_5_0_2	99.96	1	84.1	LVPVPITNATLDR_6_5_0_2	99.96	1	84.1
66.23	7494_7493	LVPVPITNATLDR_5_4_0_2	99.36	1	76.0	LVPVPITNATLDR_5_4_0_2	99.36	1	76.0	LVPVPITNATLDR_5_4_0_2	99.36	1	76.0
66.25	7497_7496	LVPVPITNATLDR_6_5_1_2	99.98	1	86.2	LVPVPITNATLDR_6_5_1_2	99.98	1	86.2	LVPVPITNATLDR_6_5_1_2	99.98	1	86.2
66.33	7512_7511	LVPVPITNATLDR_6_5_1_2	99.50	1	77.6	LVPVPITNATLDR_6_5_1_2	99.50	1	77.6	LVPVPITNATLDR_6_5_1_2	99.50	1	77.6
66.35	7516_7515	LVPVPITNATLDR_4_3_0_1	99.96	1	74.2	LVPVPITNATLDR_4_3_0_1	99.96	1	74.2	LVPVPITNATLDR_4_3_0_1	99.96	1	74.2
66.37	7519_7518	LVPVPITNATLDR_6_5_0_2	99.98	1	77.9	LVPVPITNATLDR_6_5_0_2	99.98	1	77.9	LVPVPITNATLDR_6_5_0_2	99.98	1	77.9
66.38	7521_7520	LVPVPITNATLDR_6_5_0_1	99.94	1	87.8	LVPVPITNATLDR_6_5_0_1	99.94	1	87.8	LVPVPITNATLDR_6_5_0_1	99.94	1	87.8
66.40	7525_7524	LVPVPITNATLDR_5_5_0_2	99.78	1	78.8	LVPVPITNATLDR_5_5_0_2	99.78	1	78.8	LVPVPITNATLDR_5_5_0_2	99.78	1	78.8
66.55	7552_7551	LVPVPITNATLDR_5_4_0_2	99.90	1	80.9	LVPVPITNATLDR_5_4_0_2	99.90	1	80.9	LVPVPITNATLDR_5_4_0_2	99.90	1	80.9
66.59	7560_7559	LVPVPITNATLDR_6_5_0_2	99.87	1	85.0	LVPVPITNATLDR_6_5_0_2	99.87	1	85.0	LVPVPITNATLDR_6_5_0_2	99.87	1	85.0
66.96	7629_7628	LVPVPITNATLDR_6_5_0_2	99.92	1	82.3	LVPVPITNATLDR_6_5_0_2	99.92	1	82.3	LVPVPITNATLDR_6_5_0_2	99.92	1	82.3

67.03	7642_7641	LVPVPITNATLDR_7_6_0_3	99.64	1	78.2	LVPVPITNATLDR_7_6_0_3	99.64	1	78.2	LVPVPITNATLDR_7_6_0_3	99.64	1	78.2
67.05	7646_7645	LVPVPITNATLDR_7_6_0_3	99.33	1	70.0	LVPVPITNATLDR_7_6_0_3	99.33	1	70.0	LVPVPITNATLDR_7_6_0_3	99.33	1	70.0
67.33	7695_7694	LVPVPITNATLDR_6_5_1_3	99.95	1	81.7	LVPVPITNATLDR_6_5_1_3	99.95	1	81.7	LVPVPITNATLDR_6_5_1_3	99.95	1	81.7
67.35	7699_7698	LVPVPITNATLDR_6_5_0_3	99.93	1	86.7	LVPVPITNATLDR_6_5_0_3	99.93	1	86.7	LVPVPITNATLDR_6_5_0_3	99.93	1	86.7
67.42	7712_7711	LVPVPITNATLDR_6_5_0_2	99.90	1	88.4	LVPVPITNATLDR_6_5_0_2	99.90	1	88.4	LVPVPITNATLDR_6_5_0_2	99.90	1	88.4
67.45	7717_7716	LVPVPITNATLDR_6_5_1_3	99.96	1	84.1	LVPVPITNATLDR_6_5_1_3	99.96	1	84.1	LVPVPITNATLDR_6_5_1_3	99.96	1	84.1
67.52	7730_7729	LVPVPITNATLDR_5_4_0_2	99.92	1	76.9	LVPVPITNATLDR_5_4_0_2	99.92	1	76.9	LVPVPITNATLDR_5_4_0_2	99.92	1	76.9
67.63	7750_7749	LVPVPITNATLDR_6_5_0_3	99.99	1	70.0	LVPVPITNATLDR_6_5_0_3	99.99	1	70.0	LVPVPITNATLDR_6_5_0_3	99.99	1	70.0
67.69	7761_7760	LVPVPITNATLDR_6_5_0_3	99.97	1	82.7	LVPVPITNATLDR_6_5_0_3	99.97	1	82.7	LVPVPITNATLDR_6_5_0_3	99.97	1	82.7
67.77	7776_7775	LVPVPITNATLDR_6_5_0_2	99.96	1	90.8	LVPVPITNATLDR_6_5_0_2	99.96	1	90.8	LVPVPITNATLDR_6_5_0_2	99.96	1	90.8
68.01	7818_7817	LVPVPITNATLDR_6_5_0_3	99.87	1	94.1	LVPVPITNATLDR_6_5_0_3	99.87	1	94.1	LVPVPITNATLDR_6_5_0_3	99.87	1	94.1
68.09	7833_7832	LVPVPITNATLDR_6_5_0_3	99.80	1	82.6	LVPVPITNATLDR_6_5_0_3	99.80	1	82.6	LVPVPITNATLDR_6_5_0_3	99.80	2	82.6
68.10	7835_7834	LVPVPITNATLDQITGK_6_5_0_2	99.43	1	77.4	LVPVPITNATLDQITGK_6_5_0_2	99.43	1	77.4	LVPVPITNATLDQITGK_6_5_0_2	99.43	2	77.4
68.82	7963_7962	CANLVPVPITNATLDR_6_5_0_2	99.82	1	73.7	CANLVPVPITNATLDR_6_5_0_2	99.82	1	73.7	CANLVPVPITNATLDR_6_5_0_2	99.82	3	73.7
69.28	8047_8046	LVPVPITNATLDQITGK_6_5_1_0	99.80	1	92.5	LVPVPITNATLDQITGK_6_5_1_0	99.80	1	92.5	LVPVPITNATLDQITGK_6_5_1_0	99.80	1	92.5
69.38	8066_8065	LVPVPITNATLDQITGK_6_5_0_0	99.88	1	88.1	LVPVPITNATLDQITGK_6_5_0_0	99.88	1	88.1	LVPVPITNATLDQITGK_6_5_0_0	99.88	1	88.1
69.47	8082_8081	LVPVPITNATLDQITGK_6_5_0_0	99.66	1	76.9	LVPVPITNATLDQITGK_6_5_0_0	99.66	1	76.9	LVPVPITNATLDQITGK_6_5_0_0	99.66	1	76.9
69.62	8110_8109	LVPVPITNATLDQITGK_5_4_0_0	99.71	1	92.0	LVPVPITNATLDQITGK_5_4_0_0	99.71	1	92.0	LVPVPITNATLDQITGK_5_4_0_0	99.71	1	92.0
69.65	8115_8114	LVPVPITNATLDQITGK_5_4_0_0	99.88	1	85.2	LVPVPITNATLDQITGK_5_4_0_0	99.88	1	85.2	LVPVPITNATLDQITGK_5_4_0_0	99.88	1	85.2
69.74	8130_8129	LVPVPITNATLDQITGK_5_4_0_0	99.84	1	72.8	LVPVPITNATLDQITGK_5_4_0_0	99.84	1	72.8	LVPVPITNATLDQITGK_5_4_0_0	99.84	1	72.8

70.07	8185_8184	LVPVPITNATLDQITGK_3_3_0_0	99.93	1	92.3	LVPVPITNATLDQITGK_3_3_0_0	99.93	1	92.3	LVPVPITNATLDQITGK_3_3_0_0	99.93	1	92.3
70.24	8214_8213	CANLVPVPITNATLDR_6_5_0_3	99.78	1	75.9	CANLVPVPITNATLDR_6_5_0_3	99.78	1	75.9	LSVATNVSATLTFNTSK_6_5_0_3	99.81	1	78.6
70.58	8271_8270	LVPVPITNATLDQITGK_7_6_0_1	99.87	1	83.4	LVPVPITNATLDQITGK_7_6_0_1	99.87	1	83.4	LVPVPITNATLDQITGK_7_6_0_1	99.87	1	83.4
70.72	8295_8294	LVPVPITNATLDQITGK_7_6_1_1	99.82	1	84.5	LVPVPITNATLDQITGK_7_6_1_1	99.82	1	84.5	LVPVPITNATLDQITGK_7_6_1_1	99.82	2	84.5
70.89	8324_8323	LVPVPITNATLDQITGK_6_5_1_1	99.87	1	93.5	LVPVPITNATLDQITGK_6_5_1_1	99.87	1	93.5	LVPVPITNATLDQITGK_6_5_1_1	99.87	1	93.5
71.04	8350_8349	LVPVPITNATLDQITGK_6_5_0_1	99.81	1	88.5	LVPVPITNATLDQITGK_6_5_0_1	99.81	1	88.5	LVPVPITNATLDQITGK_6_5_0_1	99.81	1	88.5
71.10	8361_8360	LVPVPITNATLDQITGK_5_4_0_1	99.76	1	85.6	LVPVPITNATLDQITGK_5_4_0_1	99.76	1	85.6	LVPVPITNATLDQITGK_5_4_0_1	99.76	1	85.6
71.13	8366_8365	LVPVPITNATLDQITGK_6_5_0_1	99.98	1	93.7	LVPVPITNATLDQITGK_6_5_0_1	99.98	1	93.7	LVPVPITNATLDQITGK_6_5_0_1	99.98	1	93.7
71.15	8370_8369	LVPVPITNATLDQITGK_6_5_0_0	99.66	1	77.8	LVPVPITNATLDQITGK_6_5_0_0	99.66	1	77.8	LVPVPITNATLDQITGK_6_5_0_0	99.66	1	77.8
71.17	8374_8373	LVPVPITNATLDQITGK_5_4_0_0	99.71	1	83.5	LVPVPITNATLDQITGK_5_4_0_0	99.71	1	83.5	LVPVPITNATLDQITGK_5_4_0_0	99.71	1	83.5
71.21	8381_8380	LVPVPITNATLDQITGK_5_4_0_0	99.60	1	80.1	LVPVPITNATLDQITGK_5_4_0_0	99.60	1	80.1	LVPVPITNATLDQITGK_5_4_0_0	99.60	1	80.1
71.25	8388_8387	LVPVPITNATLDQITGK_5_5_0_1	99.95	1	83.5	LVPVPITNATLDQITGK_5_5_0_1	99.95	1	83.5	LVPVPITNATLDQITGK_5_5_0_1	99.95	1	83.5
71.32	8401_8400	LVPVPITNATLDQITGK_5_4_0_1	99.95	1	86.8	LVPVPITNATLDQITGK_5_4_0_1	99.95	1	86.8	LVPVPITNATLDQITGK_5_4_0_1	99.95	1	86.8
71.38	8412_8411	LVPVPITNATLDQITGK_6_5_0_1	99.95	1	79.4	LVPVPITNATLDQITGK_6_5_0_1	99.95	1	79.4	LVPVPITNATLDQITGK_6_5_0_1	99.95	1	79.4
71.43	8421_8420	LVPVPITNATLDQITGK_5_4_0_1	99.98	1	81.7	LVPVPITNATLDQITGK_5_4_0_1	99.98	1	81.7	LVPVPITNATLDQITGK_5_4_0_1	99.98	1	81.7
71.48	8429_8428	LVPVPITNATLDQITGK_4_3_0_1	99.52	1	87.5	LVPVPITNATLDQITGK_4_3_0_1	99.52	1	87.5	LVPVPITNATLDQITGK_4_3_0_1	99.52	1	87.5
71.54	8440_8439	LVPVPITNATLDQITGK_4_3_0_1	99.71	1	88.0	LVPVPITNATLDQITGK_4_3_0_1	99.71	1	88.0	LVPVPITNATLDQITGK_4_3_0_1	99.71	1	88.0
71.57	8445_8444	LVPVPITNATLDQITGK_4_3_0_0	99.81	1	80.2	LVPVPITNATLDQITGK_4_3_0_0	99.81	1	80.2	LVPVPITNATLDQITGK_4_3_0_0	99.81	1	80.2
71.67	8462_8461	LVPVPITNATLDQITGK_4_4_0_1	99.97	1	72.7	LVPVPITNATLDQITGK_4_4_0_1	99.97	1	72.7	LVPVPITNATLDQITGK_4_4_0_1	99.97	1	72.7
71.70	8467_8466	LVPVPITNATLDQITGK_5_4_0_1	99.98	1	82.0	LVPVPITNATLDQITGK_5_4_0_1	99.98	1	82.0	LVPVPITNATLDQITGK_5_4_0_1	99.98	1	82.0

71.79	8482_8481	LVPVPITNATLDQITGK_8_7_0_2	99.42	1	75.0	LVPVPITNATLDQITGK_8_7_0_2	99.42	1	75.0	LVPVPITNATLDQITGK_8_7_0_2	99.42	1	75.0
71.83	8489_8488	LVPVPITNATLDQITGK_5_4_0_1	99.89	1	72.6	LVPVPITNATLDQITGK_5_4_0_1	99.89	1	72.6	LVPVPITNATLDQITGK_5_4_0_1	99.89	1	72.6
72.00	8517_8516	LVPVPITNATLDQITGK_4_3_0_1	99.91	1	80.0	LVPVPITNATLDQITGK_4_3_0_1	99.91	1	80.0	LVPVPITNATLDQITGK_4_3_0_1	99.91	1	80.0
72.06	8526_8525	LVPVPITNATLDQITGK_7_6_0_2	99.43	1	82.1	LVPVPITNATLDQITGK_7_6_0_2	99.43	1	82.1	LVPVPITNATLDQITGK_7_6_0_2	99.43	3	82.1
72.22	8553_8552	LVPVPITNATLDQITGK_7_6_1_2	99.35	1	80.7	LVPVPITNATLDQITGK_7_6_1_2	99.35	1	80.7	LVPVPITNATLDQITGK_7_6_1_2	99.35	2	80.7
72.25	8559_8558	LVPVPITNATLDQITGK_6_5_0_1	99.10	1	70.0	LVPVPITNATLDQITGK_6_5_0_1	99.10	1	70.0	LVPVPITNATLDQITGK_6_5_0_1	99.10	1	70.0
72.41	8586_8585	LVPVPITNATLDQITGK_7_6_0_2	99.88	1	89.0	LVPVPITNATLDQITGK_7_6_0_2	99.88	1	89.0	LVPVPITNATLDQITGK_7_6_0_2	99.88	4	89.0
72.55	8610_8609	LVPVPITNATLDQITGK_6_5_1_2	99.88	1	85.3	LVPVPITNATLDQITGK_6_5_1_2	99.88	1	85.3	LVPVPITNATLDQITGK_6_5_1_2	99.88	2	85.3
72.64	8627_8626	LVPVPITNATLDQITGK_5_4_0_2	99.56	1	76.0	LVPVPITNATLDQITGK_5_4_0_2	99.56	1	76.0	LVPVPITNATLDQITGK_5_4_0_2	99.56	1	76.0
72.66	8630_8629	LVPVPITNATLDQITGK_6_5_0_2	99.95	1	85.1	LVPVPITNATLDQITGK_6_5_0_2	99.95	1	85.1	LVPVPITNATLDQITGK_6_5_0_2	99.95	2	85.1
72.70	8638_8637	LVPVPITNATLDQITGK_5_4_0_1	99.83	1	89.6	LVPVPITNATLDQITGK_5_4_0_1	99.83	1	89.6	LVPVPITNATLDQITGK_5_4_0_1	99.83	1	89.6
72.72	8641_8640	LVPVPITNATLDQITGK_6_5_0_2	99.96	1	86.3	LVPVPITNATLDQITGK_6_5_0_2	99.96	1	86.3	LVPVPITNATLDQITGK_6_5_0_2	99.96	2	86.3
72.74	8645_8644	LVPVPITNATLDQITGK_3_3_0_0	99.77	1	78.5	LVPVPITNATLDQITGK_3_3_0_0	99.77	1	78.5	LVPVPITNATLDQITGK_3_3_0_0	99.77	1	78.5
72.92	8676_8675	LVPVPITNATLDQITGK_6_5_1_2	99.57	1	77.0	LVPVPITNATLDQITGK_6_5_1_2	99.57	1	77.0	LVPVPITNATLDQITGK_6_5_1_2	99.57	1	77.0
72.95	8682_8681	LVPVPITNATLDQITGK_5_5_0_2	99.91	1	78.0	LVPVPITNATLDQITGK_5_5_0_2	99.91	1	78.0	LVPVPITNATLDQITGK_5_5_0_2	99.91	1	78.0
72.97	8685_8684	LVPVPITNATLDQITGK_5_4_0_2	99.91	1	82.1	LVPVPITNATLDQITGK_5_4_0_2	99.91	1	82.1	LVPVPITNATLDQITGK_5_4_0_2	99.91	1	82.1
72.98	8687_8686	LVPVPITNATLDQITGK_6_5_0_1	99.89	1	70.0	LVPVPITNATLDQITGK_6_5_0_1	99.89	1	70.0	LVPVPITNATLDQITGK_6_5_0_1	99.89	1	70.0
73.03	8696_8695	LVPVPITNATLDQITGK_6_5_0_2	99.94	1	74.8	LVPVPITNATLDQITGK_6_5_0_2	99.94	1	74.8	LVPVPITNATLDQITGK_6_5_0_2	99.94	2	74.8
73.04	8698_8697	LVPVPITNATLDQITGK_5_4_0_2	99.97	1	76.8	LVPVPITNATLDQITGK_5_4_0_2	99.97	1	76.8	LVPVPITNATLDQITGK_5_4_0_2	99.97	1	76.8
73.09	8707_8706	LVPVPITNATLDQITGK_6_5_0_2	99.91	1	81.5	LVPVPITNATLDQITGK_6_5_0_2	99.91	1	81.5	LVPVPITNATLDQITGK_6_5_0_2	99.91	3	81.5

73.12	8713_8712	LVPVPITNATLDQITGK_5_4_0_1	99.94	1	92.9	LVPVPITNATLDQITGK_5_4_0_1	99.94	1	92.9	LVPVPITNATLDQITGK_5_4_0_1	99.94	1	92.9
73.29	8742_8741	LVPVPITNATLDQITGK_8_7_0_3	99.62	1	73.9	LVPVPITNATLDQITGK_8_7_0_3	99.62	1	73.9	LVPVPITNATLDQITGK_8_7_0_3	99.62	1	73.9
73.34	8751_8750	LVPVPITNATLDQITGK_5_4_0_2	99.97	1	70.0	LVPVPITNATLDQITGK_5_4_0_2	99.97	1	70.0	LVPVPITNATLDQITGK_5_4_0_2	99.97	1	70.0
73.54	8786_8785	SVQEIQATFFYFTPNK_6_5_0_0	99.86	1	85.5	SVQEIQATFFYFTPNK_6_5_0_0	99.86	1	85.5	SVQEIQATFFYFTPNK_6_5_0_0	99.86	5	85.5
73.56	8788_8787	LVPVPITNATLDQITGK_7_6_0_3	98.98	1	79.9	LVPVPITNATLDQITGK_7_6_0_3	98.98	1	79.9	LVPVPITNATLDQITGK_7_6_0_3	98.98	1	79.9
73.70	8812_8811	LVPVPITNATLDQITGK_7_6_1_3	99.36	1	82.6	LVPVPITNATLDQITGK_7_6_1_3	99.36	1	82.6	LVPVPITNATLDQITGK_7_6_1_3	99.36	1	82.6
73.75	8821_8820	SVQEIQATFFYFTPNK_6_5_1_0	99.77	1	72.2	SVQEIQATFFYFTPNK_6_5_1_0	99.77	1	72.2	SVQEIQATFFYFTPNK_6_5_1_0	99.77	1	72.2
73.80	8830_8829	LVPVPITNATLDQITGK_6_5_0_2	99.94	1	89.5	LVPVPITNATLDQITGK_6_5_0_2	99.94	1	89.5	LVPVPITNATLDQITGK_6_5_0_2	99.94	2	89.5
73.84	8836_8835	LVPVPITNATLDQITGK_6_5_0_2	99.93	1	72.6	LVPVPITNATLDQITGK_6_5_0_2	99.93	1	72.6	LVPVPITNATLDQITGK_6_5_0_2	99.93	2	72.6
73.92	8850_8849	SVQEIQATFFYFTPNK_6_5_0_0	99.94	1	81.6	SVQEIQATFFYFTPNK_6_5_0_0	99.94	1	81.6	SVQEIQATFFYFTPNK_6_5_0_0	99.94	2	81.6
73.93	8852_8851	LVPVPITNATLDQITGK_7_6_0_3	99.82	1	78.5	LVPVPITNATLDQITGK_7_6_0_3	99.82	1	78.5	LVPVPITNATLDQITGK_7_6_0_3	99.82	5	78.5
74.12	8885_8884	LVPVPITNATLDQITGK_6_5_0_3	99.83	1	84.0	LVPVPITNATLDQITGK_6_5_0_3	99.83	1	84.0	LVPVPITNATLDQITGK_6_5_0_3	99.83	2	84.0
74.13	8887_8886	LVPVPITNATLDQITGK_6_5_1_3	99.89	1	84.4	LVPVPITNATLDQITGK_6_5_1_3	99.89	1	84.4	LVPVPITNATLDQITGK_6_5_1_3	99.89	2	84.4
74.19	8898_8897	LVPVPITNATLDQITGK_5_4_0_2	99.96	1	87.5	LVPVPITNATLDQITGK_5_4_0_2	99.96	1	87.5	LVPVPITNATLDQITGK_5_4_0_2	99.96	1	87.5
74.33	8924_8923	LVPVPITNATLDQITGK_6_5_0_2	99.49	1	78.4	LVPVPITNATLDQITGK_6_5_0_2	99.49	1	78.4	LVPVPITNATLDQITGK_6_5_0_2	99.49	1	78.4
74.47	8949_8948	LVPVPITNATLDQITGK_6_5_0_3	99.97	1	79.9	LVPVPITNATLDQITGK_6_5_0_3	99.97	1	79.9	LVPVPITNATLDQITGK_6_5_0_3	99.97	2	79.9
74.50	8955_8954	LVPVPITNATLDQITGK_6_5_1_3	99.89	1	73.8	LVPVPITNATLDQITGK_6_5_1_3	99.89	1	73.8	LVPVPITNATLDQITGK_6_5_1_3	99.89	1	73.8
74.55	8964_8963	LVPVPITNATLDQITGK_5_4_0_2	99.88	1	74.7	LVPVPITNATLDQITGK_5_4_0_2	99.88	1	74.7	LVPVPITNATLDQITGK_5_4_0_2	99.88	1	74.7
74.56	8966_8965	LVPVPITNATLDQITGK_6_5_1_3	99.81	1	69.6	LVPVPITNATLDQITGK_6_5_1_3	99.81	1	69.6	LVPVPITNATLDQITGK_6_5_1_3	99.81	2	69.6
74.74	8997_8996	SVQEIQATFFYFTPNK_8_7_0_1	99.47	1	75.6	SVQEIQATFFYFTPNK_8_7_0_1	99.47	1	75.6	SVQEIQATFFYFTPNK_8_7_0_1	99.47	2	75.6

74.85	9015_9014	LVPVPITNATLDQITGK_6_5_0_3	99.81	1	82.6	LVPVPITNATLDQITGK_6_5_0_3	99.81	1	82.6	LVPVPITNATLDQITGK_6_5_0_3	99.81	4	82.6
75.02	9043_9042	LVPVPITNATLDQITGK_6_5_0_2	99.86	1	88.7	LVPVPITNATLDQITGK_6_5_0_2	99.86	1	88.7	LVPVPITNATLDQITGK_6_5_0_2	99.86	2	88.7
75.20	9070_9069	SVQEIQATFFYFTPNK_7_6_0_1	99.90	1	77.9	SVQEIQATFFYFTPNK_7_6_0_1	99.90	1	77.9	SVQEIQATFFYFTPNK_7_6_0_1	99.90	2	77.9
75.23	9074_9073	SVQEIQATFFYFTPNK_6_5_0_1	99.86	1	82.4	SVQEIQATFFYFTPNK_6_5_0_1	99.86	1	82.4	SVQEIQATFFYFTPNK_6_5_0_1	99.86	1	82.4
75.37	9098_9097	SVQEIQATFFYFTPNK_6_5_0_0	99.49	1	89.8	SVQEIQATFFYFTPNK_6_5_0_0	99.49	1	89.8	SVQEIQATFFYFTPNK_6_5_0_0	99.49	6	89.8
75.58	9133_9132	SVQEIQATFFYFTPNK_6_5_1_1	99.53	1	74.2	SVQEIQATFFYFTPNK_6_5_1_1	99.53	1	74.2	SVQEIQATFFYFTPNK_6_5_1_1	99.53	1	74.2
75.60	9136_9135	SVQEIQATFFYFTPNK_7_6_0_1	99.98	1	77.9	SVQEIQATFFYFTPNK_7_6_0_1	99.98	1	77.9	SVQEIQATFFYFTPNK_7_6_0_1	99.98	2	77.9
75.61	9138_9137	SVQEIQATFFYFTPNK_6_5_0_1	99.89	1	91.0	SVQEIQATFFYFTPNK_6_5_0_1	99.89	1	91.0	SVQEIQATFFYFTPNK_6_5_0_1	99.89	1	91.0
75.67	9149_9148	SVQEIQATFFYFTPNK_6_5_0_1	99.86	1	91.1	SVQEIQATFFYFTPNK_6_5_0_1	99.86	1	91.1	SVQEIQATFFYFTPNK_6_5_0_1	99.86	3	91.1
75.81	9173_9172	SVQEIQATFFYFTPNK_6_5_1_1	99.86	1	72.3	SVQEIQATFFYFTPNK_6_5_1_1	99.86	2	72.3	SVQEIQATFFYFTPNK_6_5_1_1	99.86	3	72.3
75.83	9177_9176	SVQEIQATFFYFTPNK_5_4_0_1	99.93	1	81.1	SVQEIQATFFYFTPNK_5_4_0_1	99.93	1	81.1	SVQEIQATFFYFTPNK_5_4_0_1	99.93	2	81.1
75.97	9202_9201	SVQEIQATFFYFTPNK_6_5_0_1	99.95	1	80.4	SVQEIQATFFYFTPNK_6_5_0_1	99.95	1	80.4	SVQEIQATFFYFTPNK_6_5_0_1	99.95	1	80.4
76.05	9215_9214	SVQEIQATFFYFTPNK_6_5_0_1	99.47	1	76.5	SVQEIQATFFYFTPNK_6_5_0_1	99.47	1	76.5	SVQEIQATFFYFTPNK_6_5_0_1	99.47	1	76.5
76.08	9221_9220	LVPVPITNATLDQITGK_6_5_2_2	98.45	2	80.8	LVPVPITNATLDQITGK_6_5_2_2	98.45	2	80.8	LVPVPITNATLDQITGK_6_5_2_2	98.45	7	80.8
76.16	9235_9234	SVQEIQATFFYFTPNK_5_4_0_1	99.98	1	91.6	SVQEIQATFFYFTPNK_5_4_0_1	99.98	1	91.6	SVQEIQATFFYFTPNK_5_4_0_1	99.98	1	91.6
76.41	9279_9278	LVPVPITNATLDR_9_8_1_2	98.50	2	70.8	LVPVPITNATLDR_9_8_1_2	98.50	2	70.8	LVPVPITNATLDR_9_8_1_2	98.50	16	70.8
76.49	9292_9291	SVQEIQATFFYFTPNK_4_4_0_1	99.96	1	86.0	SVQEIQATFFYFTPNK_4_4_0_1	99.96	1	86.0	SVQEIQATFFYFTPNK_4_4_0_1	99.96	1	86.0
76.55	9301_9300	SVQEIQATFFYFTPNK_5_4_0_1	99.88	1	83.7	SVQEIQATFFYFTPNK_5_4_0_1	99.88	1	83.7	SVQEIQATFFYFTPNK_5_4_0_1	99.88	1	83.7
77.06	9391_9390	SVQEIQATFFYFTPNK_7_6_1_2	99.62	1	81.1	SVQEIQATFFYFTPNK_7_6_1_2	99.62	1	81.1	SVQEIQATFFYFTPNK_7_6_1_2	99.62	2	81.1
77.42	9455_9454	SVQEIQATFFYFTPNK_7_6_0_2	99.96	1	70.0	SVQEIQATFFYFTPNK_7_6_0_2	99.96	1	70.0	EGYSNISYIVVNHQGISSR_7_5_0_2	99.81	7	77.2

77.51	9472_9471	SVQEIQATFFYFTPNK_6_5_0_2	99.97	1	91.3	SVQEIQATFFYFTPNK_6_5_0_2	99.97	1	91.3	SVQEIQATFFYFTPNK_6_5_0_2	99.97	1	91.3
77.67	9501_9500	SVQEIQATFFYFTPNK_5_4_0_1	99.59	1	84.2	SVQEIQATFFYFTPNK_5_4_0_1	99.59	1	84.2	SVQEIQATFFYFTPNK_5_4_0_1	99.59	3	84.2
77.73	9510_9509	SVQEIQATFFYFTPNK_6_5_0_2	99.94	1	74.8	SVQEIQATFFYFTPNK_6_5_0_2	99.94	1	74.8	SVQEIQATFFYFTPNK_6_5_0_2	99.94	3	74.8
77.74	9512_9511	SVQEIQATFFYFTPNK_6_5_0_1	99.90	1	89.4	SVQEIQATFFYFTPNK_6_5_0_1	99.90	1	89.4	SVQEIQATFFYFTPNK_6_5_0_1	99.90	2	89.4
77.80	9523_9522	SVQEIQATFFYFTPNK_6_5_1_2	99.95	1	74.6	SVQEIQATFFYFTPNK_6_5_1_2	99.95	1	74.6	SVQEIQATFFYFTPNK_6_5_1_2	99.95	1	74.6
77.81	9525_9524	SVQEIQATFFYFTPNK_6_5_1_2	99.66	1	84.7	SVQEIQATFFYFTPNK_6_5_1_2	99.66	1	84.7	SVQEIQATFFYFTPNK_6_5_1_2	99.66	6	84.7
77.86	9534_9533	SVQEIQATFFYFTPNK_6_5_0_2	99.95	1	82.6	SVQEIQATFFYFTPNK_6_5_0_2	99.95	1	82.6	SVQEIQATFFYFTPNK_6_5_0_2	99.95	3	82.6
78.05	9565_9564	SVQEIQATFFYFTPNK_5_4_0_2	99.98	1	84.5	SVQEIQATFFYFTPNK_5_4_0_2	99.98	1	84.5	SVQEIQATFFYFTPNK_5_4_0_2	99.98	1	84.5
78.06	9567_9566	SVQEIQATFFYFTPNK_5_4_0_2	99.63	1	83.0	SVQEIQATFFYFTPNK_5_4_0_2	99.63	1	83.0	SVQEIQATFFYFTPNK_5_4_0_2	99.63	5	83.0
78.08	9571_9570	SVQEIQATFFYFTPNK_5_4_0_1	99.75	1	76.4	SVQEIQATFFYFTPNK_5_4_0_1	99.75	1	76.4	SVQEIQATFFYFTPNK_5_4_0_1	99.75	3	76.4
78.11	9576_9575	SVQEIQATFFYFTPNK_6_5_0_2	99.96	1	78.8	SVQEIQATFFYFTPNK_6_5_0_2	99.96	1	78.8	SVQEIQATFFYFTPNK_6_5_0_2	99.96	4	78.8
78.22	9595_9594	SVQEIQATFFYFTPNK_5_4_1_2	98.98	1	74.3	SVQEIQATFFYFTPNK_5_4_1_2	98.98	1	74.3	SVQEIQATFFYFTPNK_5_4_1_2	98.98	8	74.3
78.25	9600_9599	SVQEIQATFFYFTPNK_6_5_0_2	99.94	1	76.6	SVQEIQATFFYFTPNK_6_5_0_2	99.94	1	76.6	SVQEIQATFFYFTPNK_6_5_0_2	99.94	3	76.6
78.44	9631_9630	SVQEIQATFFYFTPNK_5_4_0_2	99.94	1	80.4	SVQEIQATFFYFTPNK_5_4_0_2	99.94	1	80.4	SVQEIQATFFYFTPNK_5_4_0_2	99.94	3	80.4
78.46	9635_9634	SVQEIQATFFYFTPNK_5_4_0_2	99.84	1	77.5	SVQEIQATFFYFTPNK_5_4_0_2	99.84	1	77.5	SVQEIQATFFYFTPNK_5_4_0_2	99.84	1	77.5
78.52	9644_9643	SVQEIQATFFYFTPNK_6_5_0_2	99.86	1	70.0	SVQEIQATFFYFTPNK_6_5_0_2	99.86	1	70.0	SVQEIQATFFYFTPNK_6_5_0_2	99.86	2	70.0
78.67	9670_9669	SVQEIQATFFYFTPNK_6_5_0_2	99.82	1	87.0	SVQEIQATFFYFTPNK_6_5_0_2	99.82	1	87.0	SVQEIQATFFYFTPNK_6_5_0_2	99.82	4	87.0
78.86	9699_9698	SVQEIQATFFYFTPNK_5_4_0_2	99.72	1	71.5	SVQEIQATFFYFTPNK_5_4_0_2	99.72	1	71.5	SVQEIQATFFYFTPNK_5_4_0_2	99.72	1	71.5
79.09	9734_9733	SVQEIQATFFYFTPNK_6_5_0_2	99.67	1	82.4	SVQEIQATFFYFTPNK_6_5_0_2	99.67	1	82.4	SVQEIQATFFYFTPNK_6_5_0_2	99.67	3	82.4
79.14	9741_9740	SVQEIQATFFYFTPNK_7_6_2_3	99.54	1	78.5	SVQEIQATFFYFTPNK_7_6_2_3	99.54	1	78.5	SVQEIQATFFYFTPNK_7_6_2_3	99.54	1	78.5

79.36	9776_9775	SVQEIQATFFYFTPNK_6_5_0_2	99.59	1	77.7	SVQEIQATFFYFTPNK_6_5_0_2	99.59	1	77.7	SVQEIQATFFYFTPNK_6_5_0_2	99.59	11	77.7
79.42	9787_9786	SVQEIQATFFYFTPNK_6_5_0_3	99.86	1	76.3	SVQEIQATFFYFTPNK_6_5_0_3	99.86	1	76.3	SVQEIQATFFYFTPNK_6_5_0_3	99.86	1	76.3
79.45	9791_9790	SVQEIQATFFYFTPNK_7_6_0_2	99.58	1	75.7	SVQEIQATFFYFTPNK_7_6_0_2	99.58	1	75.7	SVQEIQATFFYFTPNK_7_6_0_2	99.58	1	75.7
79.50	9800_9799	SVQEIQATFFYFTPNK_6_5_1_3	99.89	1	74.8	SVQEIQATFFYFTPNK_6_5_1_3	99.89	1	74.8	SVQEIQATFFYFTPNK_6_5_1_3	99.89	1	74.8
79.68	9831_9830	SVQEIQATFFYFTPNK_5_4_0_2	99.66	1	77.3	SVQEIQATFFYFTPNK_5_4_0_2	99.66	1	77.3	SVQEIQATFFYFTPNK_5_4_0_2	99.66	6	77.3
79.72	9837_9836	SVQEIQATFFYFTPNK_6_5_0_2	99.89	1	82.6	SVQEIQATFFYFTPNK_6_5_0_2	99.89	1	82.6	SVQEIQATFFYFTPNK_6_5_0_2	99.89	4	82.6
79.75	9842_9841	SVQEIQATFFYFTPNK_6_5_0_3	99.91	1	85.6	SVQEIQATFFYFTPNK_6_5_0_3	99.91	1	85.6	SVQEIQATFFYFTPNK_6_5_0_3	99.91	7	85.6
79.76	9844_9843	SVQEIQATFFYFTPNK_6_5_0_2	99.92	1	70.0	SVQEIQATFFYFTPNK_6_5_0_2	99.92	1	70.0	SVQEIQATFFYFTPNK_6_5_0_2	99.92	3	70.0
79.80	9851_9850	SVQEIQATFFYFTPNK_6_5_0_3	99.98	1	73.7	SVQEIQATFFYFTPNK_6_5_0_3	99.98	1	73.7	SVQEIQATFFYFTPNK_6_5_0_3	99.98	1	73.7
79.82	9855_9854	SVQEIQATFFYFTPNK_7_6_1_3	99.84	1	82.4	SVQEIQATFFYFTPNK_7_6_1_3	99.84	1	82.4	SVQEIQATFFYFTPNK_7_6_1_3	99.84	1	82.4
79.87	9862_9861	SVQEIQATFFYFTPNK_6_5_1_3	99.58	1	82.8	SVQEIQATFFYFTPNK_6_5_1_3	99.58	1	82.8	SVQEIQATFFYFTPNK_6_5_1_3	99.58	1	82.8
80.08	9897_9896	SVQEIQATFFYFTPNK_5_4_0_2	99.87	1	75.8	SVQEIQATFFYFTPNK_5_4_0_2	99.87	1	75.8	SVQEIQATFFYFTPNK_5_4_0_2	99.87	5	75.8
80.15	9908_9907	SVQEIQATFFYFTPNK_6_5_0_3	99.80	1	71.8	SVQEIQATFFYFTPNK_6_5_0_3	99.80	1	71.8	SVQEIQATFFYFTPNK_6_5_0_3	99.80	9	71.8
80.16	9910_9909	SVQEIQATFFYFTPNK_6_5_0_2	99.89	1	70.0	SVQEIQATFFYFTPNK_6_5_0_2	99.89	1	70.0	SVQEIQATFFYFTPNK_6_5_0_2	99.89	3	70.0
80.24	9923_9922	SVQEIQATFFYFTPNK_7_6_1_3	99.48	1	76.9	SVQEIQATFFYFTPNK_7_6_1_3	99.48	1	76.9	SVQEIQATFFYFTPNK_7_6_1_3	99.48	1	76.9
80.36	9943_9942	SVQEIQATFFYFTPNK_6_5_1_3	99.81	1	76.1	SVQEIQATFFYFTPNK_6_5_1_3	99.81	1	76.1	SVQEIQATFFYFTPNK_6_5_1_3	99.81	1	76.1
80.51	9967_9966	SVQEIQATFFYFTPNK_5_4_0_2	99.90	1	75.1	SVQEIQATFFYFTPNK_5_4_0_2	99.90	1	75.1	SVQEIQATFFYFTPNK_5_4_0_2	99.90	1	75.1
80.54	9972_9971	SVQEIQATFFYFTPNK_7_6_0_3	99.95	1	77.1	SVQEIQATFFYFTPNK_7_6_0_3	99.95	1	77.1	SVQEIQATFFYFTPNK_7_6_0_3	99.95	1	77.1
80.59	9980_9979	SVQEIQATFFYFTPNK_6_5_0_2	99.11	1	73.1	SVQEIQATFFYFTPNK_6_5_0_2	99.11	1	73.1	SVQEIQATFFYFTPNK_6_5_0_2	99.11	6	73.1
80.61	9983_9982	SVQEIQATFFYFTPNK_6_5_0_3	99.92	1	72.8	SVQEIQATFFYFTPNK_6_5_0_3	99.92	1	72.8	SVQEIQATFFYFTPNK_6_5_0_3	99.92	1	72.8

80.78	10009_10008	SVQEIQATFFYFTPNK_6_5_1_3	99.79	1	80.1	SVQEIQATFFYFTPNK_6_5_1_3	99.79	1	80.1	SVQEIQATFFYFTPNK_6_5_1_3	99.79	3	80.1
81.46	10123_10122	SVQEIQATFFYFTPNK_6_5_0_2	99.53	1	69.9	SVQEIQATFFYFTPNK_6_5_0_2	99.53	1	69.9	SVQEIQATFFYFTPNK_6_5_0_2	99.53	3	69.9
81.63	10148_10147	SVQEIQATFFYFTPNK_7_6_0_3	99.94	1	70.3	SVQEIQATFFYFTPNK_7_6_0_3	99.94	1	70.3	SVQEIQATFFYFTPNK_7_6_0_3	99.94	1	70.3
81.64	10150_10149	SVQEIQATFFYFTPNK_6_5_0_3	99.79	1	70.2	SVQEIQATFFYFTPNK_6_5_0_3	99.79	1	70.2	SVQEIQATFFYFTPNK_6_5_0_3	99.79	7	70.2
81.74	10165_10164	SVQEIQATFFYFTPNK_6_5_0_2	99.72	1	74.6	SVQEIQATFFYFTPNK_6_5_0_2	99.72	1	74.6	SVQEIQATFFYFTPNK_6_5_0_2	99.72	1	74.6
82.08	10214_10213	SVQEIQATFFYFTPNK_7_6_0_3	99.77	1	73.6	SVQEIQATFFYFTPNK_7_6_0_3	99.77	1	73.6	SVQEIQATFFYFTPNK_7_6_0_3	99.77	3	73.6
82.09	10216_10215	SVQEIQATFFYFTPNK_6_5_0_3	99.39	1	78.8	SVQEIQATFFYFTPNK_6_5_0_3	99.39	1	78.8	SVQEIQATFFYFTPNK_6_5_0_3	99.39	10	78.8
82.17	10229_10228	SVQEIQATFFYFTPNK_6_5_0_2	99.03	1	74.6	SVQEIQATFFYFTPNK_6_5_0_2	99.03	1	74.6	SVQEIQATFFYFTPNK_6_5_0_2	99.03	3	74.6
83.02	10348_10347	SVQEIQATFFYFTPNK_7_6_0_3	99.58	1	73.2	SVQEIQATFFYFTPNK_7_6_0_3	99.58	1	73.2	SVQEIQATFFYFTPNK_7_6_0_3	99.58	3	73.2
83.65	10431_10430	SVQEIQATFFYFTPNK_7_6_0_2	99.39	1	72.5	SVQEIQATFFYFTPNK_7_6_0_2	99.39	1	72.5	SVQEIQATFFYFTPNK_7_6_0_2	99.39	3	72.5
87.20	10878_10877	SVQEIQATFFYFTPNK_7_6_0_4	99.54	1	76.1	SVQEIQATFFYFTPNK_7_6_0_4	99.54	1	76.1	SVQEIQATFFYFTPNK_7_6_0_4	99.54	2	76.1

RT; retention time, Scan; scan number from raw file, S.Rank; ranking order of *N*-glycopeptide candidates with S-score; Glycopeptide nomenclature; PEP_#Hex_#HexNac_#Fuc_#NeuAc

Supplementary Table 6. Comparison of numbers of *N*-glycoproteins and *N*-glycopeptides (glycoprotein, filtered spectra, and unique *N*-glycopeptide) identified from a mixture of seven standard glycoproteins (RNase B; IgG 1, 2, 3, and 4; and AGP 1, and 2) using different MS/MS fragmentations (CID, HCD, and CID/HCD) and databases of different sizes for the id-GPA search.

Database	MS/MS mode	Protein	number of unique glycoproteins	number of filtered spectra	number of unique *N*-glycopeptides	Threshold of Y-score (Estimated FDR)
GPA-DB-Mixture*	HCD+CID	RNase B	1	7	3	62.0 (0.72%)
		IgG	3	43	26	
		AGP	2	86	15	
GPA-DB-HumanPlasma** (add RNase B)	HCD+CID	RNase B	1	7	3	63.0 (0.75%)
		IgG	3	43	26	
		AGP	2	83	15	

* 7 glycoproteins(RNaseB, IgG1,2,3,&4, AGP1 &2),   ** 282 glycoproteins + RNase B

Supplementary Table 7. Results of label-free quantitation by 3TIQ for the calibration curve of spiked RNase B.

No.	Concentration (μg)	Replicate	*N*-glycopeptides from RNase B				
			NLTK_5200 (2+)	NLTK_6200 (2+)	NLTK_7200 (2+)	NLTK_8200 (2+)	NLTK_920 0(2+)
1	0.0375	1	175,290	104,826	ND	ND	ND
		2	211832	108366	ND	ND	ND
		3	219644	121640	ND	ND	ND
		average	202255	111610			
		CV%	11.71	7.94			
2	0.0750	1	427673	235753	ND	135969	ND
		2	396238	187738	ND	102280	ND
		3	454039	226078	ND	129216	ND
		average	425983	216523		122488	
		CV%	6.79	11.73		14.55	
3	0.1500	1	697671	456603	ND	271298	131462
		2	898168	511769	ND	334999	166069
		3	708777	356626	ND	165347	82355
		average	768205	441666		257215	126629
		CV%	14.67	17.81		33.32	33.22
4	0.5000	1	2770462	1474932	470657	1001904	451297
		2	2951072	1697265	459994	871672	457228
		3	2632418	1498797	419940	897729	446576
		average	2784650	1556998	450197	923768	451700
		CV%	5.74	7.84	5.94	7.46	1.18
5	1.0000	1	6711896	3718008	940203	1737008	886463
		2	6339925	3906035	1051287	2094245	967159
		3	7035946	4096952	988455	2030821	1008362
		average	6695922	3906998	993315	1954025	953995
		CV%	5.20	4.85	5.61	9.75	6.50
6	2.0000	1	10264766	6294057	1826972	4591736	2235814
		2	10952696	6761400	1855130	4921004	2445628
		3	12898644	7683530	2143566	3018212	1372176
		average	11372035	6912996	1941889	4176984	2017873
		CV%	12.01	10.23	9.02	24.35	28.20

ND : Not detected

Supplementary Table 8. Summary of the numbers of selected spectra, *N*-glycopeptides, estimated FDRs, and manually validated FDRs generated by I-GPA and Byonic for the standard α1-acid glycoprotein (AGP) data.

	I-GPA	Byonic
Number of selected *N*-glycopeptide HCD spectra (Automatic score cut)	1,674 (M-score>1.31)	-
Number of selected *N*-glycopeptide candidate spectra	924 (S-score>98.0)	-
Number of identified *N*-glycopeptide spectra / unique *N*-glycopeptides (Automatic score cut)	456 / 95 (Y-score>69.5)	342 / 111 (Byonic score>0)
Estimated FDR (Automatic calculation)	0.9%[a]	0.0%[b]
Number of manually validated spectra / unique *N*-glycopeptides (True positive N-GSMs[c])	456 / 95	248 / 71
Manually validated FDR	0.0%	27.5%

[a]Estimated FDR values calculated by GPA decoy method.

[b]Sz-Wei Wu et al. [5].

[c]N-Glycopeptide spectrum matches (N-GSMs)

Supplementary Table 9. Comparison of *N*-glycopeptide profiles of IgG1 in the reference vs. our data (non-depleted normal plasma).

Glycopeptides	Huffman et al. 2014 MCP (LC-ESI-MS)	Non-depleted plasma (normal)	
	EEQYNSTYR (IgG1)	EEQYNSTYR (IgG1)	CVs (%)
EEQYNSTYR_3_3_0_0	–	0.6	5.6
EEQYNSTYR_3_3_1_0	–	5.66	1.5
EEQYNSTYR_3_4_0_0	–	0.92	15.3
EEQYNSTYR_3_4_1_0	21.79	17.53 (22.04*)	8.4
EEQYNSTYR_3_5_1_0	7.99	3.37 (4.24*)	6.7
EEQYNSTYR_4_3_0_0	–	0.79	4.2
EEQYNSTYR_4_3_1_0	–	5.98	3.8
EEQYNSTYR_4_3_1_1	–	0.14	18.8
EEQYNSTYR_4_4_0_0	–	2.54	6.6
EEQYNSTYR_4_4_1_0	30.02	27.36 (34.38*)	4.3
EEQYNSTYR_4_4_1_1	2.21	0.59 (0.74*)	60.2
EEQYNSTYR_4_5_0_0	–	0.37	19.9
EEQYNSTYR_4_5_1_0	10.44	5.19 (6.53*)	7.0
EEQYNSTYR_4_5_1_1	–	0.15	18.2
EEQYNSTYR_5_4_0_0	–	2.41	7.9
EEQYNSTYR_5_4_0_1	–	0.73	26.8
EEQYNSTYR_5_4_1_0	13.59	18.99 (23.86*)	5.6
EEQYNSTYR_5_4_1_1	12.24	5.66 (7.11*)	18.6
EEQYNSTYR_5_5_1_0	1.72	0.87 (1.09*)	18.1
EEQYNSTYR_5_5_1_1	–	0.13	19.1
% of agalatosylated compositions	29.78	28.09	4.6
% of monogalactosylated compositions	42.67	43.12	2.4
% of digalactosylated compositions	27.56	28.79	4.3

*The normalized percentage of N-glycopeptides in the case of consideration with only N-glycopeptides shown in reference
